# A Novel Methoxybenzyl 5-Nitroacridone Derivative Effectively Triggers G1 Cell Cycle Arrest in Chronic Myelogenous Leukemia K562 Cells by Inhibiting CDK4/6-Mediated Phosphorylation of Rb

**DOI:** 10.3390/ijms21145077

**Published:** 2020-07-18

**Authors:** Bin Zhang, Ting Zhang, Tian-Yi Zhang, Ning Wang, Shan He, Bin Wu, Hai-Xiao Jin

**Affiliations:** 1 Li Dak Sum Yip Yio Chin Kenneth Li Marine Biopharmaceutical Research Center, Department of Marine Pharmacy, College of Food and Pharmaceutical Sciences, Ningbo University, Ningbo 315800, Zhejiang, China; binzhang86@126.com (B.Z.); zt326311@163.com (T.Z.); zty3215310hdzf@163.com (T.-Y.Z.); heshan@nbu.edu.cn (S.H.); 2 State Key Laboratory of Chemical Oncogenomics, Key Laboratory of Chemical Biology, Tsinghua Shenzhen International Graduate School, Shenzhen 518055, China; 3 Institute of Drug Discovery Technology, Ningbo University, Ningbo 315800, Zhejiang, China; 4 Ocean College, Zhejiang University, Hangzhou 310058, China; wubin@zju.edu.cn

**Keywords:** metabolomics, chronic myeloid leukemia, acridone derivatives, CDK4/6, anti-leukemia mechanism

## Abstract

Chronic myeloid leukemia (CML) is a malignant tumor caused by the abnormal proliferation of hematopoietic stem cells. Among a new series of acridone derivatives previously synthesized, it was found that the methoxybenzyl 5-nitroacridone derivative **8q** has nanomolar cytotoxicity in vitro against human chronic myelogenous leukemia K562 cells. In order to further explore the possible anti-leukemia mechanism of action of **8q** on K562 cells, a metabolomics and molecular biology study was introduced. It was thus found that most of the metabolic pathways of the G1 phase of K562 cells were affected after **8q** treatment. In addition, a concentration-dependent accumulation of cells in the G1 phase was observed by cell cycle analysis. Western blot analysis showed that **8q** significantly down-regulated the phosphorylation level of retinoblastoma-associated protein (Rb) in a concentration-dependent manner, upon 48 h treatment. In addition, **8q** induced K562 cells apoptosis, through both mitochondria-mediated and exogenous apoptotic pathways. Taken together, these results indicate that **8q** effectively triggers G1 cell cycle arrest and induces cell apoptosis in K562 cells, by inhibiting the CDK4/6-mediated phosphorylation of Rb. Furthermore, the possible binding interactions between **8q** and CDK4/6 protein were clarified by homology modeling and molecular docking. In order to verify the inhibitory activity of **8q** against other chronic myeloid leukemia cells, KCL-22 cells and K562 adriamycin-resistant cells (K562/ADR) were selected for the MTT assay. It is worth noting that **8q** showed significant anti-proliferative activity against these cell lines after 48 h/72 h treatment. Therefore, this study provides new mechanistic information and guidance for the development of new acridones for application in the treatment of CML.

## 1. Introduction

Leukemia is a malignant clonal disease of hematopoietic stem cells that can be divided into acute and chronic types. At present, these hematological cancers are the 10th most common cause of death in the world [1]. Chronic myeloid leukemia (CML) is among the chronic leukemia types, and accounts for about 15–20% of all cases of leukemia in diagnosed patients [2]. The survival rate of many CML patients has been greatly improved after treatment with tyrosine kinase inhibitors such as imatinib and nilotinib. Nevertheless, the appearance of resistance can be a serious problem in the treatment of CML [3]. Therefore, it is important to develop new anticancer agents with different molecular targets or mechanisms of action for the treatment of CML. As a class of anticancer compounds, acridines and acridones have generally shown good cytotoxic or anticancer activity against acute and chronic leukemia cells [4,5,6]. For instance, amsacrine (*m*-AMSA) has been used clinically in a number of countries for the treatment of acute leukemia [7,8].

Among a new series of acridones synthesized previously [4], it was discovered that *N*-[2-(dimethylamino)ethyl]-1-[(3-methoxybenzyl)amino]-5-nitro-9-oxo-9,10-dihydro-acridine-4-carboxamide (**8q**) significantly induces mitochondrial-mediated apoptosis and targets the PI3K/AKT/FOXO1 pathway in human acute lymphoblastic leukemia CCRF-CEM cells [9]. Interestingly, it was also found that **8q** has potent nanomolar cytotoxic activity against human chronic myelogenous leukemia K562 cells. At the same time, this molecule showed a relatively low level of toxicity based on other in vitro data. Moreover, in silico calculations showed that **8q** conforms to Lipinski’s rule of five, and is predicted to have good pharmacokinetic properties. However, the anti-chronic leukemia mechanism of action for this compound is still unclear. Metabolomics is a tool that can be used to effectively study the mechanisms of anti-cancer drugs, due to the impact of the cancer on cellular metabolism. Herein, a sensitive and accurate ultra-performance liquid chromatography/time-of-flight mass spectrometry (UPLC/Q-TOF MS) instrument was used to conduct the metabolomics profiling of chronic myelogenous leukemia K562 cells treated with the benzyl acridone analogue **8q**. Furthermore, molecular biology methods, homology modeling and molecular docking strategies were employed in this study to further investigate the possible anti-leukemia mechanism for this molecule.

## 2. Results

### 2.1. Multivariate Statistical Analysis and Changes in Metabolites

**Figure 1:**
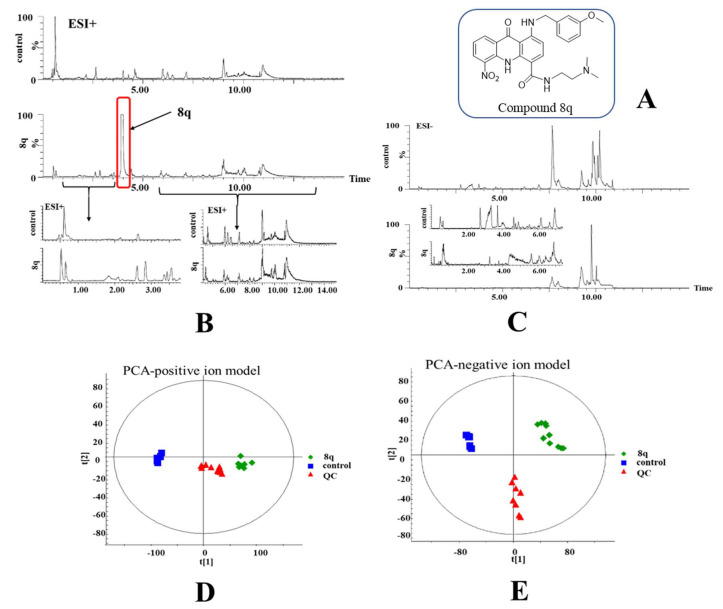
LC-MS based metabolomics study of K562 cells treated with **8q**. (**A**) Chemical structure of **8q**; (**B**) base peak intensity (BPI) chromatograms obtained from control and treated K562 cell extracts in positive ion mode; (**C**) and negative ion mode; (**D**) PCA of positive ion mode data; (**E**) PCA of negative ion mode data.

Metabolomics was used to study the possible mechanism of action for **8q** on K562 cells. The chemical structure of **8q** is shown in Figure 1A. At first, the MTT experiment was conducted to evaluate the optimal drug concentration to use for the metabolomics study. K562 cells were treated with different concentrations of **8q** (0–2.5 μM) for 24 h (Figure S1). When the concentration of **8q** was 0.5 μM, the cell viability was about 80%, and was deemed suitable for metabolomics research. UPLC/Q-TOF MS was used to study the changes in metabolic profiles between treated and untreated cells. As shown in Figure 1B,C, the respective base peak intensity (BPI) chromatograms had significant differences after **8q** treatment in both positive and negative MS detection modes. It is noteworthy that a high concentration of **8q** was detected in **8q**-treated cells, which indicated that **8q** can enter the cell membrane and is not immediately metabolized. By principal component analysis (PCA, Figure 1D,E), it was shown that the metabolites of the control (DMSO) and **8q**-treated group were obviously separated along the first principal component. In addition, the QC samples were tightly clustered in the middle of PCA scores, indicating the stability of the system throughout the experimental study. Therefore, these results show that the cellular metabolic phenotypes were significantly changed after treatment with **8q**. Next, the different metabolites between control and **8q**-treated group were assessed by independent t-test and false discovery rate. The q value was found to be less than 0.05, which indicated that there were significant differences between the two groups, and the corresponding metabolites were selected as potential biomarkers. A total of 119 significantly changed metabolites were identified in different ion modes, including 33 metabolites in the positive ion mode and 86 metabolites in the negative ion mode. Potential biomarkers were identified based on HR-MS spectra and MS/MS fragments, and compared with the corresponding standards in the database (HMDB, METLIN and lipid map). Metabolites in positive and negative ion modes are summarized in Table 1 and Table 2, respectively. The results of matched MS/MS spectra metabolites have also been appended in Tables S1 and S2.

### 2.2. Metabolic Pathway Analysis

**Figure 2:**
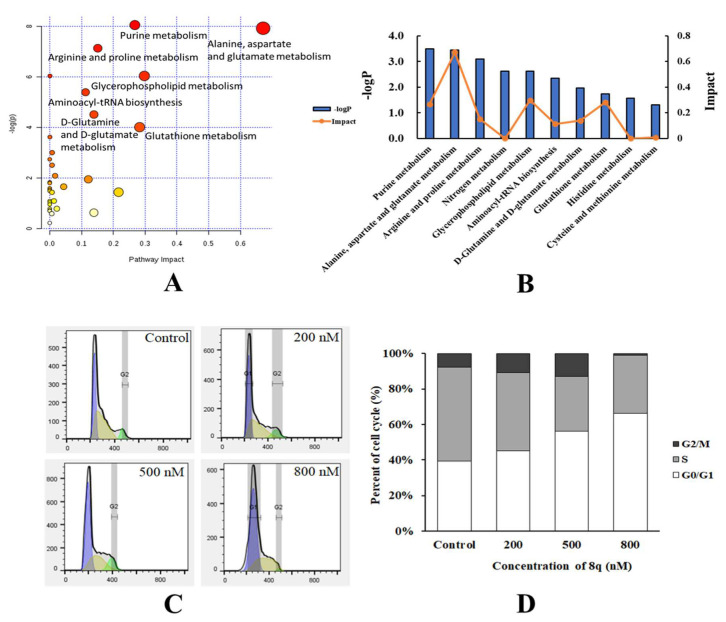
(**A**) Metabolic pathways altered by cell treatment with **8q**. (**B**) Quantitative analysis of the impact of **8q** on metabolic pathways. (**C**) Flow cytometric analysis of **8q** induction of G0/G1 phase cell cycle arrest in K562 cells. K562 cells were treated with **8q** at the indicated concentrations for 48 h. (**D**) Cell cycle phase analysis of K562 cells treated with **8q**.

Changes in metabolic pathways caused by **8q**-treated cells were analyzed by the available online tools Biochemical Pathways (http://biochemical-pathways.com) and MetaboAnalyst 3.0 (http://www.metaboanalyst.ca). As shown in Figure 2A,B, the metabolic pathways disturbed by **8q** mainly included purine metabolism, alanine, aspartate and glutamate metabolism, arginine and proline metabolism, glycerophospholipid metabolism, d-glutamine and d-glutamate, aminoacyl-tRNA biosynthesis and glutathione metabolism. Purines supply the essential synthesis substrates for DNA and RNA [10], and purine metabolism is often abnormally increased in tumor cells for rapid growth and proliferation that mainly affects the G1 phase of the cell cycle [11]. Amino acids are essential for the synthesis of proteins and aminoacyl tRNA that are also especially important in the G1 phase [12,13]. The metabolites here detected in five amino acid (alanine, aspartate, glutamate, arginine, and proline) metabolic pathways are also involved in aminoacyl-tRNA biosynthesis [14]. As shown in Tables S1 and S2, it was observed that the concentrations of l-glutamine, l-aspartate, l-leucine, l-proline and l-glutamate were all decreased after **8q** treatment. Hence, it was speculated that aminoacyl-tRNA biosynthesis was inhibited. Aminoacyl-tRNA biosynthesis is occurring at the G1 phase, as catalyzed by tRNA synthase [15]. When tRNA synthase is inhibited, the aminoacyl-tRNA biosynthesis is blocked and cells will arrest in the G1 phase. Therefore, flow cytometry analysis can be used for further investigating the effect of **8q** on the cell cycle based on the above results.

### 2.3. **8q** Effectively Triggered G1 Cell Cycle Arrest in K562 Cells

After the treatment of K562 cells with **8q** at 200 nM, 500 nM and 800 nM for 48 h, the cells were found to be arrested in the G1 phase of the cell cycle in a concentration-dependent manner. As shown in Figure 2C,D, the cell population in G0/G1 increased from 37.7% in untreated cells to 45.4%, 56.6% and 65.8% in the cells treated with **8q** at 200 nM, 500 nM and 800 nM, respectively. That is, the data show that treatment with **8q** results in G1 cell cycle arrest in K562 cells.

### 2.4. **8q** Showed Concentration-Dependent Inhibition of the Phosphorylation of the CDK4/6 Substrate Rb

**Figure 3:**
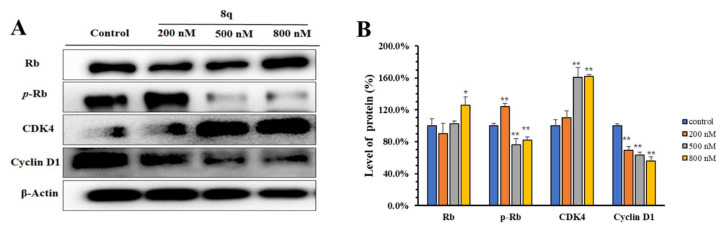
(**A**) **8q** inhibited CDK 4/6-mediated phosphorylation of Rb; (**B**) The densitometry of proteins performed on the Western blotting of A, * *p* < 0.05; ** *p* < 0.01.

CDK4/6 is a specific protein of the G1 phase that binds with cyclin D to form a complex, promotes phosphorylation of target retinoblastoma-associated protein (Rb), and then allows the cell cycle to enter the S phase [16]. The phosphorylation of Rb is required for the G1-S transition of the cell cycle. Therefore, selective inhibition of Rb phosphorylation can cause the arrest of the cell cycle in G1 phase. As shown in Western blots (Figure 3A,B), **8q** (200–800 nM) significantly inhibits CDK 4/6-mediated phosphorylation of Rb, and the expression of Rb protein increased slightly, in a concentration-dependent manner. In addition, the level of cyclin D1 in K562 cells also decreased in a concentration-dependent manner, upon 48 h treatment with **8q**. Meanwhile, the level of CDK4 was found to increase dramatically. It may be that **8q** affects the formation of CDK4 and cyclin D1 complexes, which is the key step to regulate Rb [17,18]. Accordingly, based on the results of the Western blotting and cell cycle analysis, it is suggested that **8q** effectively triggers G1 cell cycle arrest in K562 cells by inhibiting CDK4/6-mediated phosphorylation of Rb.

### 2.5. Homology Modeling of CDK4/6 Protein and Molecular Docking

**Figure 4:**
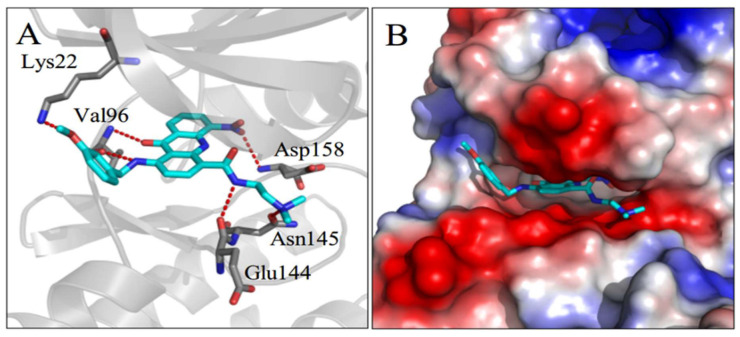
(**A**) CDK4 is depicted in grey cartoon form with grey stick residues involved in hydrogen bonding, and **8q** is shown as a cyan stick model. Oxygen atoms and nitrogen atoms are depicted in red and blue, respectively. Hydrogen bonds are shown as red dashed lines. (**B**) CDK4 is represented in electrostatic surface form, with blue and red indicating positive and negative charges respectively.

CDK4/6 inhibitors inhibit the progression of cancer cells from G1 to S phase and trigger G1 cell cycle arrest by selectively inhibiting the function of CDK4/6 [19]. After metabolomics and molecular biology studies performed here revealed that the benzyl acridone **8q** might be a CDK4/6 inhibitor that down-regulates the phosphorylation level of downstream protein Rb, further studies were designed to evaluate this hypothesis. Therefore, the binding interactions between **8q** and CDK4/6 were analyzed in silico by homology modeling and molecular docking. Some CDK4 X-ray crystal structures have been obtained and reported, but all of them are in an inactive state in which the activation loop flaps over and partially closes the active site. CDK6 is a homologous protein of CDK4 with a high percentage of residue identity (71.3%) and similar physiological function. Homology modeling was used to build an active CDK4 structure (Figure S2) according to a reported active CDK6 crystal structure (PDB ID: 2EUF). When docked in the model system, **8q** formed several hydrogen (H) bonds with CDK4. It had two H bonds with Val96 in the hinge region, which are conserved H bonds among known CDK4 inhibitors. The nitro group also formed one H bond with Asp158, the methoxy oxygen atom formed one H bond with Lys22, and the *N*,*N*-dimethylethylenediamine group formed another two H bonds with Glu144 and Asn145 (Figure 4A). The planar acridone ring was inserted into the active site, with its *N*,*N*-dimethylethylenediamine group extending to the negatively charged surface of CDK4 (Figure 4B). The docking result showed that **8q** is predicted to have a good complementary interaction with CDK4.

### 2.6. **8q** Significantly Induced Apoptosis in K562 Cells

**Figure 5:**
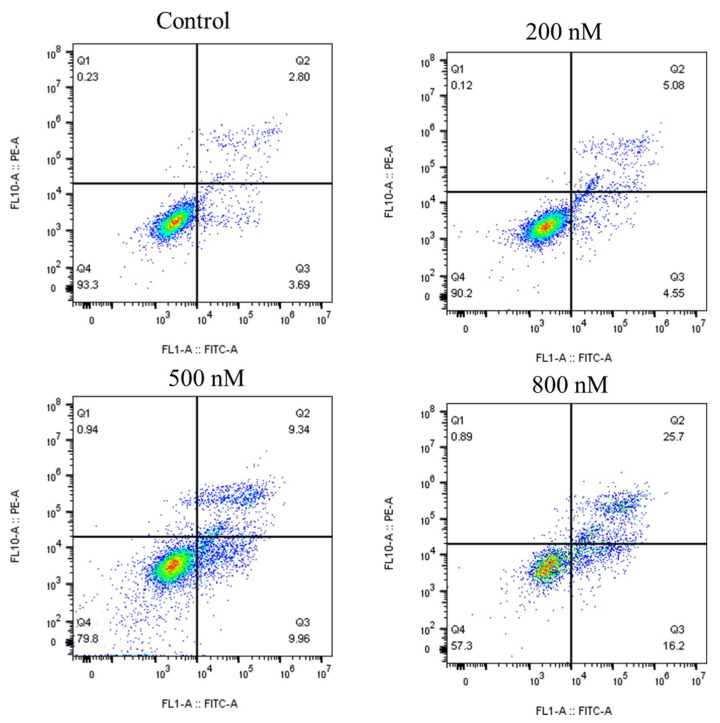
**8q** induced apoptosis in K562 cells (48 h) at different concentrations (0, 200, 500, 800 nM). DMSO as a negative control.

The work previously reported showed that **8q** has a strong antiproliferative activity that was expected to lead to apoptosis, as shown in vitro using K562 cells [9]. In order to evaluate this hypothesis, a flow cytometry assay (Annexin V-FITC/PI) was conducted in K562 cells, as shown in Figure 5. The upper right-hand quadrants (Q2) showed the late stage of apoptotic or necrotic cells. The lower right-hand quadrants (Q3) showed the early stage of apoptotic cells. K562 cells were treated with **8q** at the concentrations of 0, 200, 500 and 800 nM for 48 h. As the concentration of **8q** increased, the percentage of apoptotic cells increased from 6.49% to 41.90%. In addition, an Annexin V-FITC/PI kit assay was conducted in K562 cells, as shown in Figure S3. Annexin V-FITC can recognize early apoptotic cells and show green fluorescence. Propidium iodide (PI) was used to identify late apoptotic and necrotic cells, showing red fluorescence. The K562 cells were treated with **8q**, at concentrations of 0, 200, 500 and 800 nM for 48 h. Dimethyl sulfoxide (DMSO) was used as the negative treatment control. As the concentration of **8q** was incremented, the number of early apoptotic cells increased significantly. The early apoptosis of K562 cells was detected, starting at 200 nM of **8q**, the lowest concentration tested. When the concentration of **8q** was 800 nM, the number of late apoptotic cells also increased significantly. Additionally, the number of late apoptotic cells and necrotic cells also increased in a concentration-dependent manner. However, the trend of late apoptotic and necrotic cells was not as obvious as compared with that of early apoptotic cells. Therefore, it was concluded that **8q** effectively induces K562 cells apoptosis in a concentration-dependent manner. However, the late apoptosis and necrosis induced by **8q** could not be well distinguished in this test. Therefore, apoptosis and necrosis analyses were carried out using Hoechst 33342/PI kit. Similarly, it was confirmed that **8q** significantly induces apoptosis in K562 cells from the results shown in Figure S4. Hoechst 33342 (purchased from Beyotime Biotechnology, Shanghai, China) can be used to recognize apoptotic cells (blue fluorescence), and PI is used to identify necrotic cells (red fluorescence). Taking the above into consideration, the results suggested that **8q** induces apoptosis and inhibits the proliferation of K562 cells.

### 2.7. **8q** Induced Apoptosis through the Caspase Pathway

**Figure 6:**
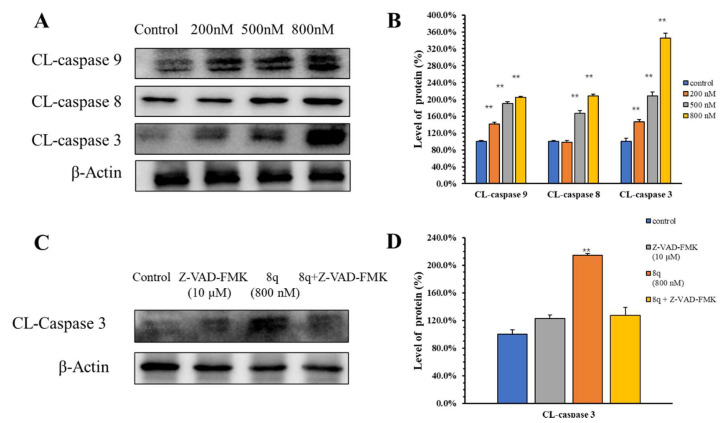
**8q** induced both mitochondria-mediated and exogenous apoptosis. (**A**) The expressions of caspase family proteins were determined after **8q** treatment; (**B**) The densitometry of caspase family proteins performed on the Western blotting of A, ** *p* < 0.01; (**C**) The expressions of caspase-3 were determined after **8q** (800 nM) or Z-VAD-FMK (10 μM) treatment; (**D**) The densitometry of caspase-3 performed on the western blotting of C, ** *p* < 0.01.

Apoptotic pathways are divided into mitochondria-mediated (intrinsic) and exogenous apoptotic pathways [20]. Caspase family proteins, such as Caspase-9, 8 and 3, are the main executors of apoptosis, and the activity changes of different caspase isoforms can distinguish the apoptosis pathways [21,22]. From the results (Figure 6A,B), we can see that **8q** activated Caspase-9, 8 and 3 proteins in a concentration-dependent manner. These results suggested that **8q** induced K562 cells apoptosis through both mitochondria-mediated and exogenous apoptotic pathways. Z-VAD-FMK is a pan-caspase inhibitor. When K562 cells were pretreated with Z-VAD-FMK (10 μM), **8q** (800 nM) could not induce the up-regulation of cleaved Caspase-3 when compared with those without Z-VAD-FMK pretreatment (Figure 6C,D). Therefore, these results demonstrate that apoptosis is the main cell death mechanism upon **8q** treatment in K562 cells.

### 2.8. In Vitro Anti-Proliferation Activity of KCL-22 and K562/ADR Cells

**Figure 7:**
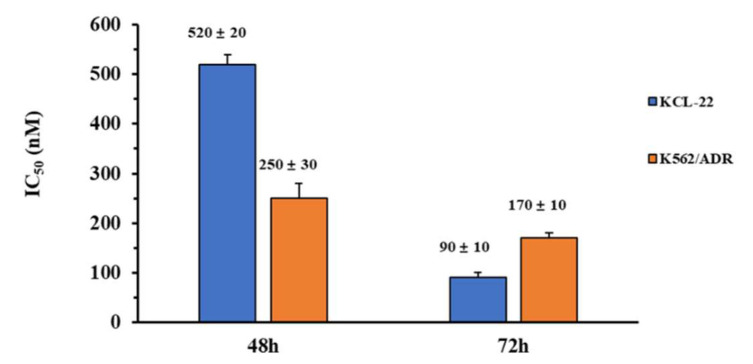
Antiproliferative activity of **8q** against KCL-22 and K562-ADR cells.

In order to verify the inhibitory activity of compound **8q** on other chronic myeloid leukemia cells, we selected KCL-22 cells for the MTT assay. In addition, human leukemia K562 adriamycin-resistant cells (K562/ADR) were also selected in the assay, to investigate whether **8q** had in vitro anti-proliferative activity against drug-resistant cell line. The results were shown in Figure 7. It is worth noting that **8q** showed obvious anti-proliferative activity against KCL-22 cells and K562/ADR cells, with IC_50_ values of 520 nM and 250 nM after 48 h treatment. In addition, when these cell lines were treated with **8q** for 72 h, we found that the inhibitory activity was significantly increased, and the IC_50_ values were 90 nM and 170 nM, respectively. Therefore, it is suggested that compound **8q** not only had good in vitro anti-proliferative activity against other CML cells, but also had significant inhibitory activity against drug-resistant leukemia cells. Thus, **8q** may be a promising hit compound in the treatment of CML.

## 3. Discussion

Our previous work reported that the new methoxybenzyl 5-nitroacridone **8q** exhibits strong anti-proliferation activity against human chronic myelogenous leukemia K562 cells and had relatively low toxicity in vitro. In this study, through the analysis of metabolic principal components and metabolic pathways, it was found that **8q** induced significant changes in the metabolites of K562 cells, and that most of the metabolic pathways affected the G1 phase of cancer cells, such as purine metabolism, aminoacyl-tRNA biosynthesis, and so on. In addition, a concentration-dependent accumulation of cells in G1 phase was observed by cell cycle analysis after treatment with **8q**. CDK4/6 is a specific protein of the G1 phase that binds with cyclin D to form a complex, promotes the phosphorylation of target Rb protein, and then allows the cell cycle to enter the S phase. Therefore, the phosphorylation of Rb is required for the G1-S transition of the cell cycle. Western blot analysis showed that the expression levels of phosphorylated Rb and cyclin D1 significantly decreased in a concentration dependent manner after **8q** treatment for 48 h, in addition, **8q** obviously increased the expression level of CDK4 in K562 cells with the increase of the drug’s concentration. Furthermore, the likely binding interactions between **8q** and CDK4/6 were clarified by in silico molecular docking studies. Therefore, these results suggest that **8q** can effectively trigger G1 cell cycle arrest in K562 cells by inhibiting CDK4/6-mediated phosphorylation of Rb.

Cell cycle arrest is often associated with cell apoptosis. This study continued to investigate whether **8q** can induce K562 cells apoptosis and further verify which apoptotic pathway it followed. Apoptosis and necrosis detection assay showed that the percentage of apoptotic cells increased from 6.49% to 41.90% as the concentration of **8q** increased. It is suggested that **8q** significantly induces cell apoptosis and inhibits the proliferation of K562 cells. Western blotting results further displayed that **8q** induced K562 cells apoptosis through both mitochondria-mediated and exogenous apoptotic pathways. In addition, we observed that, after pretreating K562 cells with Z-VAD-FMK (a pan-caspase inhibitor), the effect of **8q** on the cleavage of Caspase-3 disappeared, suggesting that **8q** induced apoptosis through the caspase pathway. Furthermore, the MTT assay showed that **8q** not only had good in vitro anti-proliferative activity against other CML cells (KCL-22 cells), but also had significant inhibitory activity against drug-resistant leukemia cells (K562/ADR cells).

In conclusion, the reported results suggest that **8q** can effectively trigger G1 cell cycle arrest and induce cell apoptosis in K562 cells by inhibiting CDK4/6-mediated phosphorylation of Rb. This study accordingly provides new information and guidance for the application of new acridone derivatives to be developed for the potential treatment of CML.

## 4. Materials and Methods

### 4.1. Reagents and Materials

*N*-(2-(dimethylamino)ethyl)-1-((3-methoxybenzyl)amino)-5-nitro-9-oxo-9,10-dihydro-acridine-4-carboxamide (**8q**) was synthesized by Zhang [4]. Z-VAD-FMK (a pan-caspase inhibitor) was purchased from Selleck (Shanghai, China). Human chronic myelogenous leukemia K562 cells were purchased from the Chinese Academy of Sciences Cell Bank. Human chronic myelogenous leukemia KCL-22 cells and K562 adriamycin-resistant cells (K562/ADR) were purchased from Zhen Shanghai and Shanghai Industrial Co., Ltd. (China). Iscove’s Modified Dubecco’s Medium (IMDM) and Fetal bovine serum (FBS) was purchased from Hyclone (Logan, Utah, USA). Cleaved Caspase-3 antibodies were purchased from Cell Signaling Technology, Inc. (Boston, Massachusetts, USA), Cleaved Caspase-9, 8 antibodies were purchased from Wuhan SanYing Co., Ltd. (Wuhan, China); other antibodies we used in this study were purchased from Beyotime Biotechnology (Shanghai, China). Annexin V-FITC/PI apoptosis detection kit, apoptosis and necrosis assay kit and cell cycle and apoptosis analysis kit were purchased from Beyotime Biotechnology (Shanghai, China). Formic acid (HPLC) was purchased from Tedia (Ohio, USA). Acetonitrile (HPLC) and methanol (HPLC) were purchased from Fisher (Waltham, Massachusetts, USA).

### 4.2. Apoptosis and Necrosis Detection Assay

Cell early and late apoptosis were detected using the Annexin V-FITC/propidium iodide (PI) apoptosis detection kit. Cell apoptosis and necrosis were detected using an apoptosis and necrosis assay kit (Hoechst 33342 and PI). Both two assays were detected after 0–800 nM of **8q** treatment for 48 h. The experimental procedure was followed according to the instructions of the manufacturer. Axio Observer 5 with an Apotome fluorescence microscope (Carl Zeiss AG, Oberkochen, Germany) was used to observe the effect of **8q** on the apoptosis and necrosis of K562 cells.

### 4.3. Metabolomics Analysis Conditions

The extraction of samples and the detection of metabolites using a UPLC/Q-TOF MS method were completed as previously described [9]. In this study, K562 cells were exposed to 500 nM of **8q**.

### 4.4. Flow Cytometric Analysis for Cell Cycle

For cell cycle analysis, K562 cells were seeded in a six-well plate and incubated for 12 h following treated with graded concentrations of **8q** for 48 h. Cells were collected and washed twice by phosphate buffered saline (PBS), then fixed in ice cold 70% ethanol. Cells were stained with 4 mg/mL PI and 0.1 mg/mL RNaseA in PBS. After incubation in the dark at room temperature for 30 min, samples were subjected to flow cytometric analysis.

### 4.5. Western Blotting

K562 cells were cultured in 6 cm dishes, followed by treatment with **8q** for different concentration periods for 48 h. Proteins were separated by electrophoresis on an 8–12% Sodium dodecyl sulfate (SDS) polyacrylamide gel and transferred to polyvinylidene difluoride membranes. The membranes were blocked with 3% bovine serum albumin and then incubated with primary antibodies, followed by a horseradish peroxidase conjugated secondary antibody and detected by the Luminescence Image Analyzer Tanon 5200.

### 4.6. In Vitro Anti-Proliferative Assay

KCL-22 cells and K562/ADR cells were seeded into 96-well plates at 0.8–1.6 × 10^4^ cells/well, treated with compound **8q**. After 48 or 72 h treatment, the cells were incubated with 15 mL MTT (3-(4, 5-dimethyl-thiazol-2-yl)-2, 5-diphenyl-tetrazolium bromide from Sigma) solution (5 mg/mL) for 4 h at 37 °C with 5% CO_2_. The formazan precipitates were dissolved in 100 mL DMSO. At 490 nm, the absorbance was measured by InfiniteM1000 PRO (TECAN).

### 4.7. Homology Modeling

The amino acid sequence of homo sapiens CDK4 (GI: 49457488) was retrieved from the NCBI website. The crystal structure of homo sapiens CDK6 [23] in active form was retrieved in protein data bank serve (PDB ID: 2EUF), and employed as the template to build the 3D structure of the active form of CDK4. The sequence of the template and CDK4 were aligned, having 71.3% sequence identity. A reliable homology model of CDK4 was predicted using Molecular Operating Environment (MOE, Chemical Computing Group, Montreal, QC, Canada). Protein geometry was checked by Ramachandran plot.

### 4.8. Molecular Docking

The molecular docking was performed on Gold suite v5.2.2 (Cambridge Crystallographic Data Centre Software Ltd., Cambridge, UK), with default genetic algorithm settings [24,25]. Docking was performed without a reference ligand. The active site residues, Ile12, Gly13, Val20, Lys22, Ala33, Val72, Arg73, Leu74, Phe93~Thr102, Glu144, Asn145, Leu147, Ala157 and Asp158, were selected as a binding pocket. Gold Score was used as the fitness function for selecting the best docked conformation of the ligand.

## Figures and Tables

**Table 1:** Summary of metabolites in positive ion mode.

Ion Model	Name	Ret. Time	*m*/*z*	∆ppm	MS/MS	*q* Value	FC ^a^
Positive ion mode	Spermine	0.4484	203.2231	0	84.0; 112.1; 129.1	0.000	0.027
	Neurine	0.5467	104.105	19	104.1	0.000	0.225
	l-Proline	0.5739	116.0691	12	70.0; 116.0	0.000	0.021
	Adenosine 3′-monophosphate	0.5884	348.0696	2	136.0; 348.0	0.000	0.034
	Glutathione	0.6219	308.09	3	58.9; 76.0; 84.0; 116.0; 130.0; 140.0; 144.0; 162.0; 179.0; 187.0; 215.0; 233.0; 245.0; 291.0; 308.0	0.000	con ^b^
	3′-Keto-3′-deoxy-AMP	0.6226	346.0473	21	136.0; 346.0	0.000	0.001
	Piperidine	0.6272	86.0936	32	69.0; 86.1	0.000	0.028
	l-Leucine	0.8145	132.1013	4	69.0; 86.0	0.000	0.026
	Piperidine	0.8172	86.094	28	69.0; 86.0	0.000	0.007
	Xestoaminol C	4.5328	230.2476	1	212.2; 230.2	0.001	0.815
	C16 Sphinganine	4.6369	274.2741	0	256.2; 274.2	0.003	con
	PC(16:0/0:0)	5.7318	496.3372	5	86.0; 184.0; 478.3; 496.3	0.000	0.097
	PC(22:2/0:0)	5.8967	576.4092	11	277.2; 559.3; 576.4	0.000	0.239
	PC(16:0/0:0)	5.9447	496.3376	4	104.1; 184.0; 258.1; 313.2; 419.2; 478.3; 496.3	0.000	0.487
	PE(18:1/0:0)	6.1807	480.3233	30	155.0; 339.2; 462.3; 480.3	0.000	0.668
	PE(18:0/0:0)	7.0864	482.3245	0	341.3; 462.2; 482.3	0.000	0.214
	PC(0:0/18:0)	7.1339	524.3699	2	104.1; 184.0; 341.3; 506.3; 524.3	0.000	0.578
	PC(20:1/0:0)	7.3169	550.3867	0	104.1; 184.0; 532.3; 553.3	0.000	0.197
	PC(18:0/18:2)	8.8141	786.6016	1	184.0; 605.5; 786.6	0.249	0.548
	PC(18:1/18:1)	9.2222	786.601	0	184.0; 522.3; 603.5; 786.6	0.024	0.297
	PC(18:3/18:1)	9.3872	782.571	2	184.0; 603.5; 782.5	0.000	0.375
	PC(18:1/18:1)	9.4376	786.6019	1	184.0; 339.2; 504.3; 522.3; 786.6	0.035	0.172
	13*E*-Docosenamide/13*Z*-Docosenamide	9.9366	338.3416	0	303.3; 321.3; 338.3	0.025	1.613
	PC(18:1/18:2)	10.7816	784.5947	12	86.0; 184.0; 504.3; 784.5	0.000	0.381
	PC(18:1/16:1)	10.7869	758.5774	10	184.0; 504.3; 758.5	0.013	0.279
	PC(18:1/18:1)	10.7904	786.6056	6	184.0; 603.5; 786.6	0.035	0.440
	PC(16:1/18:0)	10.7926	760.5899	6	86.0; 184.0; 577.5; 760.5	0.231	0.617
	PC(14:1/17:0)	10.7938	718.5497	16	184.0; 577.5; 718.5	0.010	0.338
	PC(20:3/18:0)	10.7944	812.64	29	184.0; 504.3; 812.6	0.122	0.402
	PC(P-16:0/15:1)	10.7954	702.5472	5	184.0; 702.5	0.100	0.559
	SM(d18:1/16:0)	10.7964	703.5721	3	86.0; 184.0;7 03.5	0.023	0.181
	PE(18:1/18:1)	10.7979	744.5627	11	265.2; 603.5; 744.5	0.003	0.365
	PE(18:1/0:0)	10.8012	480.3095	2	339.2; 480.3	0.000	0.614

Note: ^a^ FC means Fold change (**8q**/control); ^b^ con means control group.

**Table 2:** Summary of metabolites in negative ion mode.

Ion Model	Name	Ret. Time	*m*/*z*	∆ppm	MS/MS	*q* Value	FC ^a^
Negative ion mode	UDP-glucose/UDP-D-galactose	0.5493	565.0425	9	78.9; 96.9; 241.0; 323.0; 385.0; 565.0	0.000	0.002
	Uridine diphosphate-*N*-acetylglucosamine	0.5502	606.0683	9	78.9; 158.9; 272.9; 282.0; 384.9; 403.0; 606.0	0.000	0.002
	ADP	0.5611	426.0179	9	78.9; 134.0; 158.9; 272.9; 328.0; 408.0; 426.0	0.000	0.004
	Inosine 5′-monophosphate (IMP)	0.5714	347.0376	6	78.9; 96.9; 347.0	0.000	0.011
	5-Aminoimidazole-4-carboxamide-1-*β*-d-ribofuranosyl 5′-monophosphate	0.5799	337.0528	7	78.9; 96.9; 337.0	0.000	0.007
	l-Aspartic Acid	0.5844	132.0255	35	88.0; 114.0; 132.0	0.000	0.009
	l-Glutamate	0.5857	146.0426	22	102.0; 128.0; 146.0	0.000	0.049
	d-Glycerol 1-phosphate	0.5858	171.0083	11	78.9; 89.0; 171.0	0.000	0.012
	Sulfuric acid	0.591	96.9602	0	78.9; 96.9	0.000	0.235
	Adenosine monophosphate	0.6116	346.0515	12	78.9; 96.9; 211.0; 346.0	0.000	0.026
	l-Glutamine	0.6118	145.0599	13	102.0; 128.0; 146.9	0.000	0.003
	l-Leucine	0.7127	130.0842	24	130.0	0.000	0.077
	Cumanin	4.6125	265.1467	8	96.9; 265.1	0.007	0.143
	Dehydroabietic acid	5.6388	299.2017	0	299.2	0.000	2.076
	PE(P-16:0/0:0)	6.3388	436.2828	1	153.0; 196.0; 239.2; 436.2	0.002	1.535
	Arginyl-Glutamine	6.4253	301.1607	7	217.0; 286.1; 301.1	0.000	2.691
	Δ2-trans-Hexadecenoic Acid	6.4529	253.2172	0	84.5; 253.2	0.012	2.286
	Arachidonic Acid (peroxide free)	6.7046	303.2337	2	205.1; 259.2; 303.2	0.000	71.665
	Arginyl-Gamma-glutamate	6.8151	301.1606	7	301.1	0.000	2.560
	PE(18:0/0:0)	7.2382	480.3079	3	196.0; 283.2; 480.3	0.000	0.416
	Petroselinic acid/Oleic Acid	7.9889	281.2483	1	281.2	0.000	3.103
	11*Z*,14*Z*-Eicosadienoic Acid	8.4771	307.2647	1	307.2	0.000	7.295
	Stearic acid	9.222	283.2654	4	265.3; 283.2	0.000	2.336
	PI(20:5/18:1)	9.3598	881.5158	3	241.0; 281.2; 881.5	0.000	2.836
	cis-gondoic acid/ 2*E*-Eicosenoic acid	9.3917	309.2805	1	309.2	0.000	5.116
	PS(18:0/19:1)	9.4136	802.5604	0	283.2; 419.2; 701.5; 802.5	0.005	0.751
	PS(18:0/18:1)	9.4246	788.5407	5	152.9; 283.2; 419.2; 701.5; 788.5	0.001	0.545
	PG(18:1/22:6)	9.4363	819.525	8	281.2; 327.2; 419.2; 819.5	0.000	0.516
	PI(20:4/16:0)	9.5244	857.5121	7	241.0; 303.2; 391.2; 553.2; 857.5	0.000	3.782
	PI(20:4/18:1)	9.5572	883.5157	20	152.9; 222.9; 241.0; 303.2; 417.2; 579.2; 883.5	0.001	1.449
	PG(18:3/18:1)	9.5579	769.5007	2	152.9; 277.2; 281.2; 769.5	0.000	11.712
	PI(16:1/18:1)	9.5732	833.5135	6	152.9; 241.0; 253.2; 281.2; 389.2; 417.2; 579.2; 833.5	0.000	0.752
	PG(20:3/18:1)	9.6053	797.53	4	152.9; 281.2; 305.2; 765.6; 797.5	0.000	0.330
	PS(18:0/18:1)	9.6188	788.5404	5	152.9; 281.2; 283.2; 417.2; 419.2; 701.5; 788.5	0.000	0.086
	PG(18:1/20:4)	9.6202	795.5092	11	281.2; 303.2; 417.2; 795.5	0.000	16.215
	PI(18:1/18:2)	9.6278	859.5297	5	152.9; 241.0; 279/2; 281.2; 415.2; 417.2; 577.2; 579.2;859.5	0.007	1.146
	7,7-dimethyl-5,8-Eicosadienoic Acid	9.6238	335.2971	4	335.2	0.000	5.874
	PG(16:1/18:1)	9.6489	745.4967	7	152.9; 253.2; 281.2; 389.2; 491.2; 673.5; 745.4	0.000	17.477
	PI(20:2/20:4)	9.659	909.5474	2	241.0; 303.2; 307.2; 439.2; 443.2; 909.5	0.000	1.953
	Nervonic acid	9.6611	365.3426	0	365.3	0.000	3.921
	PG(18:2/18:1)	9.6761	771.5117	8	152.9; 279.2; 281.2; 771.5	0.000	4.204
	Docosanoic acid	9.7185	339.3293	7	339.3	0.000	3.146
	PG(18:1/17:1)	9.7688	759.5153	3	152.9; 267.2; 281.2; 759.5	0.000	8.753
	PI(20:4/18:0)	9.7744	885.546	4	223.0; 241.0; 283.2; 303.2; 419.2; 581.3; 885.5	0.000	2.928
	PI(18:0/22:5)	9.7767	911.5626	3	152.9; 241.0; 329.2; 419.2; 581.3; 607.3; 911.5	0.000	1.520
	PG(18:1/15:0)	9.7803	733.4986	5	281.2; 733.4	0.000	3.943
	PI(O-16:0/18:1)	9.8087	821.5381	20	255.2; 281.2; 437.2; 745.5; 821.5	0.018	1.521
	PA(17:0/16:1)	9.8117	659.4584	11	253.2; 659.4	0.000	6.842
	PI(18:1/18:1)	9.8177	861.5442	6	78.9; 223.0; 241.0; 281.2; 417.2; 579.2; 792.5; 861.5	0.007	0.677
	PG(18:1/18:1)	9.833	773.5242	12	152.9; 241.2; 417.2; 509.2; 773.5	0.031	1.315
	PG(20:2/18:1)	9.84	799.5471	2	152.9; 281.2; 307.2; 799.5	0.000	0.177
	PG(20:4/18:0)	9.8432	797.5334	0	152.9; 260.2; 283.2; 303.2; 419.2; 511.3; 797.5	0.000	51.407
	5,9-hexacosadienoic acid	9.8475	391.3566	3	391.3	0.001	2.517
	PI(18:1/20:2)	9.8834	887.5579	8	152.9; 223.0; 241.0; 307.2; 417.2; 443.2; 579.2; 887.5	0.000	0.308
	PG(16:0/18:1)	9.8925	747.5105	10	255.2; 281.2; 465.2; 747.5	0.000	0.437
	PI(18:1/17:0)	9.9228	849.5507	0	241.0; 281.2; 419.2; 567.3; 849.5	0.001	1.593
	PI(22:4/18:0)	9.9428	913.5793	2	152.9; 283.2; 331.2; 419.2; 443.2; 581.3; 605.3; 913.5	0.013	1.158
	PG(18:1/20:1)	9.9942	801.5621	3	152.9; 281.2; 309.2; 728.5; 801.5	0.000	0.162
	PG(22:2/18:1)	9.9951	827.5801	0	281.2; 335.2; 419.2;827.5	0.000	0.057
	PI(18:0/18:1)	10.0446	863.5568	10	152.9; 241.0; 281.2; 283.2; 417.2; 419.2; 581.3; 863.5	0.027	0.733
	Lignoceric acid	10.0555	367.3588	1	367.3	0.000	7.465
	PA(18:1/17:0)	10.0864	687.4864	15	152.9; 281.2; 423.2; 687.4	0.000	10.392
	PA(19:1/18:1)	10.0979	713.4849	38	152.9; 253.2; 281.2; 417.2; 713.4	0.000	4.158
	PI(22:2/18:1)	10.1148	915.599	2	152.9; 241.0; 417.2; 579.2; 915.5	0.000	0.475
	PI(20:2/18:0)	10.1387	889.5771	4	223.0; 241.0; 283.2; 307.2; 419.2; 443.2; 581.3; 599.3	0.000	0.380
	PG(18:0/18:1)	10.1847	775.5441	6	152.9; 281.2; 283.2; 419.2; 493.2; 511.3; 775.5	0.001	0.789
	PE(P-18:1/18:3)	10.2445	722.5086	6	152.9; 281.2; 413.1; 417.2; 722.5	0.002	0.404
	PI(22:2/20:1)	10.4522	943.6288	0	241.0; 445.2; 607.3	0.000	0.303
	PS(18:0/18:1)	10.5039	788.5415	4	152.9; 281.2; 283.2; 417.2; 419.2; 701.5; 788.5	0.020	0.212
	PI(18:0/20:1)	10.5194	891.5939	3	153.0; 223.0; 241.0; 283.2; 309.2; 419.2; 581.3; 891.5	0.003	0.020
	PI(22:2/18:0)	10.5319	917.6085	4	153.0; 223.0; 241.0; 335.2; 419.2; 581.3; 917.6	0.000	0.613
	Arachidic Acid/Phytanic Acid	10.5858	311.2971	4	311.2	0.000	2.173
	13*Z*-Docosenoic Acid	10.6089	337.3124	3	337.3	0.000	2.192
	Oleic Acid	10.8021	281.2501	5	281.2	0.001	1.746
	PA(20:2/18:1)	10.8195	725.4859	36	152.9; 281.2; 417.2; 462.3	0.011	0.607
	PE(18:1/18:1)	10.8206	742.5396	0	196.0; 281.2; 460.2; 478.2; 742.5	0.003	0.334
	PI(20:2/18:0)	10.8474	889.5809	2	241.0; 283.2; 307.2; 889.5	0.008	0.570
	PS(22:1/18:1	10.8657	842.5978	7	281.2; 755.5; 842.5	0.000	0.147
	PS(18:0/19:1	10.8682	802.5587	2	152.9; 281.2; 419.2; 710.5; 715.2; 802.5	0.000	0.420
	PS(18:0/18:1)	10.8685	788.5394	6	152.9; 283.2; 419.2; 701.5; 788.5	0.000	0.365
	PS(18:1/18:1)	10.8723	786.5269	2	152.9; 281.2; 417.2; 699.4; 701.5; 786.5	0.000	0.240
	PS(18:0/22:6)	10.8727	834.5257	3	283.2; 419.2; 463.2; 747.4; 834.5	0.000	0.350
	PS(18:1/16:0)	10.8735	760.5147	1	152.9; 255.2; 281.2; 391.2; 673.5	0.000	0.109
	PI(20:4/18:0)	10.8741	885.55	0	241.0; 419.2; 581.3; 885.5	0.000	2.550
	PG(18:0/17:1)	10.8803	761.5346	1	152.9; 283.2; 391.2; 419.2; 687.5; 761.5	0.001	0.086

Note: ^a^ FC means Fold change (**8q**/control).

## Supplementary Materials

Supplementary materials can be found online at https://www.mdpi.com/1422-0067/21/14/5077/s1.

## References

[1] Miranda-Filho A., Piñeros M., Ferlay J., Soerjomataram I., Monnereau A., Bray F. (2018). Epidemiological patterns of leukaemia in 184 countries: A population-based study. Lancet Haematol..

[2] Soverini S., De Benedittis C., Mancini M., Martinelli G. (2016). Best Practices in Chronic Myeloid Leukemia Monitoring and Management. Oncologist.

[3] Kaehler M., Nagel I., Bruckmüller H., Boehm R., Ammerpohl O., Cascorbi I. (2018). Abstract 5846: Drug resistance in chronic myeloid leukemia: Impact of methylation on gene expression in imatinib and nilotinib resistance. Cancer Res..

[4] Zhang B., Wang N., Zhang C., Gao C., Zhang W., Chen K., Wu W., Chen Y.Z., Tan C., Liu F. (2017). Novel multi-substituted benzyl acridone derivatives as survivin inhibitors for hepatocellular carcinoma treatment. Eur. J. Med. Chem..

[5] Zhang B., Chen K., Wang N., Gao C., Sun Q., Li L., Chen Y.Z., Tan C., Liu H., Jiang Y. (2015). Molecular design, synthesis and biological research of novel pyridyl acridones as potent DNA-binding and apoptosis-inducing agents. Eur. J. Med. Chem..

[6] Cui Z., Li X., Li L., Zhang B., Gao C., Chen Y., Tan C., Liu H., Xie W., Yang T. (2016). Design, synthesis and evaluation of acridine derivatives as multi-target Src and MEK kinase inhibitors for anti-tumor treatment. Bioorganic Med. Chem..

[7] Arlin Z.A. (1989). A Special Role for Amsacrine in the Treatment of Acute Leukemia. Cancer Investig..

[8] Zhang B., Li X., Li B., Gao C., Jiang Y. (2014). Acridine and its derivatives: A patent review (2009–2013). Expert Opin. Ther. Pat..

[9] Wang N., Zhang B., Jin F., Gao D., Liu F., Liu H., Jiang Y. (2018). Combing metabolomics with bioanalysis methods to study the antitumor mechanism of the new acridone derivative 8q on CCRF-CEM cells: 8q induced mitochondrial-mediated apoptosis and targeted the PI3K/AKT/FOXO1 pathway. J. Pharm. Biomed. Anal..

[10] Chan C.Y., Zhao H., Pugh R.J., Pedley A.M., French J., Jones S.A., Zhuang X., Jinnah H., Huang T.J., Benkovic S.J. (2015). Purinosome formation as a function of the cell cycle. Proc. Natl. Acad. Sci. USA.

[11] Yin J., Ren W., Huang X., Deng J., Li T., Yin J. (2018). Potential Mechanisms Connecting Purine Metabolism and Cancer Therapy. Front. Immunol..

[12] Han J.M., Kim J.Y., Kim S. (2003). Molecular network and functional implications of macromolecular tRNA synthetase complex. Biochem. Biophys. Res. Commun..

[13] Gomez M.A.R., Ibba M. (2020). Aminoacyl-tRNA Synthetases. RNA.

[14] Liang F., Kelly S., Suk N., Alexandre A., Carla P., Lennart R., Debra T.H., Dieter S.L. (2004). Aminoacyl-tRNA synthesis by pre-translational amino acid modification. RNA Biol..

[15] Liu W., Jin F., Gao D., Song L., Ding C., Liu H. (2017). Metabolomics analysis reveals aminoquinazolin derivative 9d-induced oxidative stress and cell cycle arrest in A549 cells. RSC Adv..

[16] Zha C., Deng W., Fu Y., Tang S., Lan X., Ye Y., Su Y., Jiang L., Chen Y., Huang Y. (2018). Design, synthesis and biological evaluation of tetrahydronaphthyridine derivatives as bioavailable CDK4/6 inhibitors for cancer therapy. Eur. J. Med. Chem..

[17] Harper J.W., Adami G.R., Wei N., Keyomarsi K., Elledge S.J. (1993). The p21 Cdk-interacting protein Cip1 is a potent inhibitor of G1 cyclin-dependent kinases. Cell.

[18] Jansen V.M., Bhola N.E., Bauer J.A., Formisano L., Lee K.M., Hutchinson K.E., Witkiewicz A.K., Moore P.D., Estrada M.V., Sánchez V. (2017). Kinome-wide RNA interference screen reveals a role for PDK1 in acquired resistance to CDK4/6 inhibition in ER-positive breast cancer. Cancer Res..

[19] Goel S., DeCristo M.J., McAllister S.S., Zhao J.J. (2018). CDK4/6 Inhibition in Cancer: Beyond Cell Cycle Arrest. Trends Cell Biol..

[20] Tian Y.-Z., Liu Y.-P., Tian S.-C., Ge S.-Y., Wu Y.-J., Zhang B.-L. (2020). Antitumor activity of ginsenoside Rd in gastric cancer via up-regulation of Caspase-3 and Caspase-9. Pharmazie.

[21] Indran I.R., Tufo G., Pervaiz S., Brenner C. (2011). Recent advances in apoptosis, mitochondria and drug resistance in cancer cells. Biochim. Biophys. Acta (BBA) Bioenerg..

[22] Médoc M., Dhilly M., Matesic L., Toutain J., Krauseheuer A.M., Delamare J., Fraser B.H., Touzani O., Barré L., Greguric I. (2016). In vivo evaluation of radio fluorinated caspase-3/7 inhibitors as radiotracers for apoptosis imaging and comparison with [18F] ML-10 in a stroke model in the rat. Mol. Imaging Biol..

[23] Lu H., Schulze-Gahmen U. (2006). Toward Understanding the Structural Basis of Cyclin-Dependent Kinase 6 Specific Inhibition. J. Med. Chem..

[24] Verdonk M.L., Cole J.C., Hartshorn M.J., Murray C.W., Taylor R.D. (2003). Improved protein—Ligand docking using GOLD. Proteins Struct. Funct. Bioinform..

[25] Tan K.-L., Bin Ali A., Du Y., Fu H., Jin H.-X., Chin T.-M., Khan M., Go M.-L. (2014). Synthesis and Evaluation of Bisbenzylidenedioxotetrahydrothiopranones as Activators of Endoplasmic Reticulum (ER) Stress Signaling Pathways and Apoptotic Cell Death in Acute Promyelocytic Leukemic Cells. J. Med. Chem..

