# Supplementary material for: A Novel Methoxybenzyl 5-Nitroacridone Derivative Effectively Triggers G1 Cell Cycle Arrest in Chronic Myelogenous Leukemia K562 Cells by Inhibiting CDK4/6-Mediated Phosphorylation of Rb
Source: Int J Mol Sci. 2020 Jul 18;21(14):5077. doi: 10.3390/ijms21145077 (PMC7403985; doi:10.3390/ijms21145077)
Supplement: Supplementary file 1 [file ijms-21-05077-s001.zip › Supplementary Files/Supplementary Materials-revised.docx]

***Supplementary Materials***

A novel methoxybenzyl 5-nitroacridone derivative, eﬀectively triggers G1 cell cycle arrest in chronic myelogenous leukemia K562 cells by inhibiting CDK4/6-mediated phosphorylation of Rb

Bin Zhang ^1,2^, Ting Zhang ^1^, Tian-Yi Zhang ^1^, Ning Wang ^2,3,*^, Shan He ^1^, Bin Wu ^4^ and Hai-Xiao Jin ^1,*^

1 Li Dak Sum Yip Yio Chin Kenneth Li Marine Biopharmaceutical Research Center, Department of Marine Pharmacy, College of Food and Pharmaceutical Sciences, Ningbo University, Ningbo, Zhejiang, 315800, People’s Republic of China; binzhang86@126.com(B.Z.); zt326311@163.com(T.Z.); heshan@nbu.edu.cn(S.H); 1251331039@qq.com(T.-Y.Z.); jinhaixiao@nbu.edu.cn (H.-X.J.)

2 State Key Laboratory of Chemical Oncogenomics, Key Laboratory of Chemical Biology, Tsinghua Shenzhen International Graduate School, Shenzhen, 518055, People’s Republic of China; binzhang86@126.com(B.Z.); wangning2@nbu.edu.cn (N.W.)

3 Institute of Drug Discovery Technology, Ningbo University, Ningbo, Zhejiang, 315211, People’s Republic of China; wangning2@nbu.edu.cn (N.W.)

4 Ocean College, Zhejiang University, Hangzhou 310058, China; wubin@zju.edu.cn (B.W.)

***** Correspondence: wangning2@nbu.edu.cn (N.W.); jinhaixiao@nbu.edu.cn (H.-X. J.)


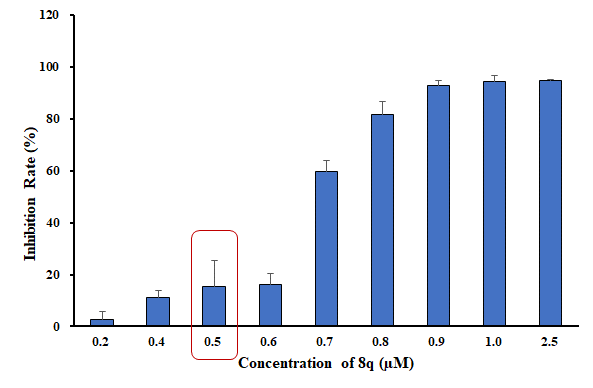


**Figure S1**. Evaluating the optimal drug concentration of **8q** in metabolomics.


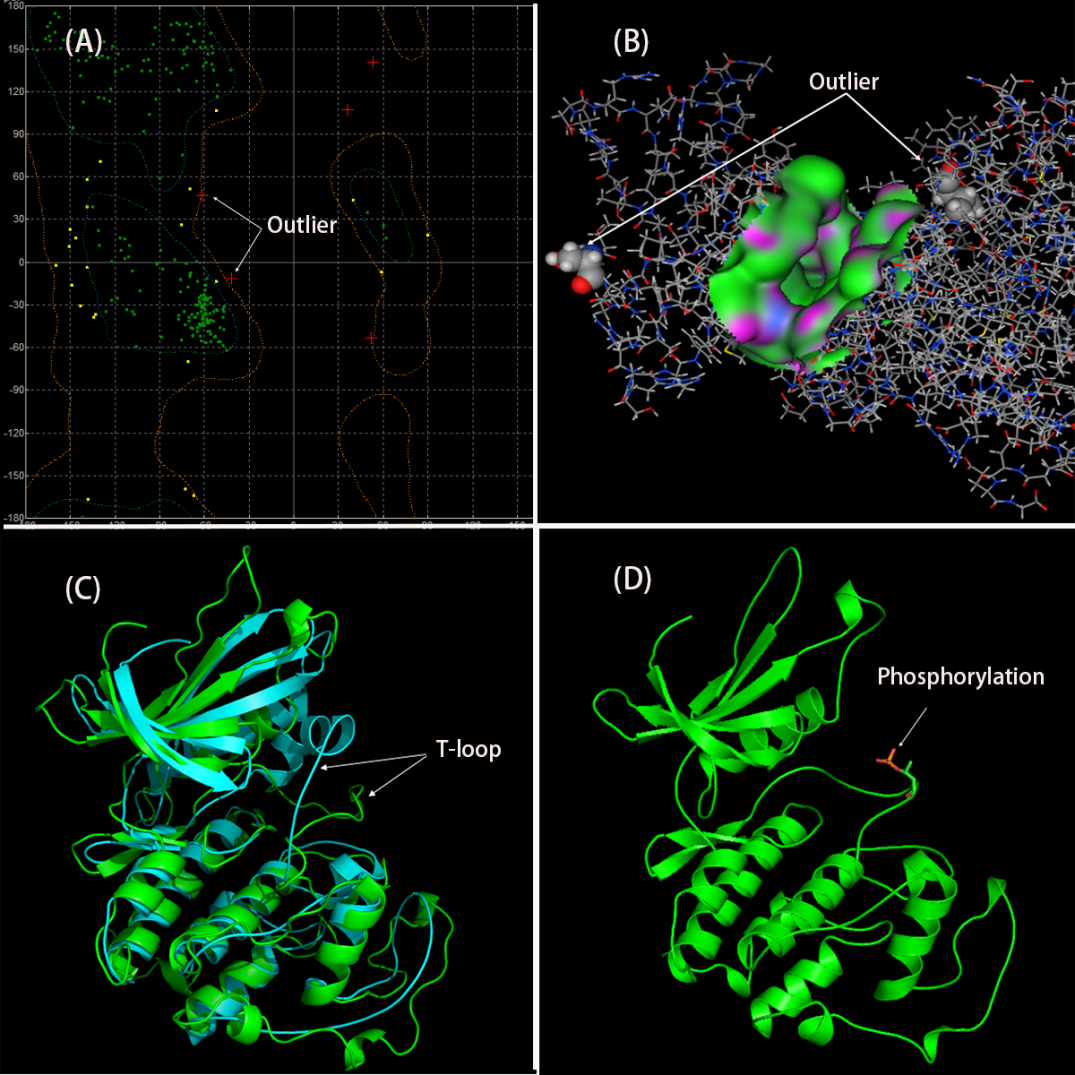


**Figure S2**. Reliable homology model of CDK4. (A) The red dots in the region are conformational aberrant amino acids (Outlier), indicating the wrong area of the modeling. (B) Outlier was displayed by space filling pattern and CDK4 active sites were displayed by surface pattern. All Outliers were not in active sites. (C) The three-dimensional conformation of CDK4 obtained by homologous modeling was superimposed and compared with CDK4 inactive conformation. It can be seen that the structure of T-loop in the three-dimensional conformation of CDK4 has changed significantly. (D) Thr172 of CDK4 protein was phosphorylated. Thr172 is a phosphorylated modification site that plays an important role in regulating the activity of CDK4 kinase.


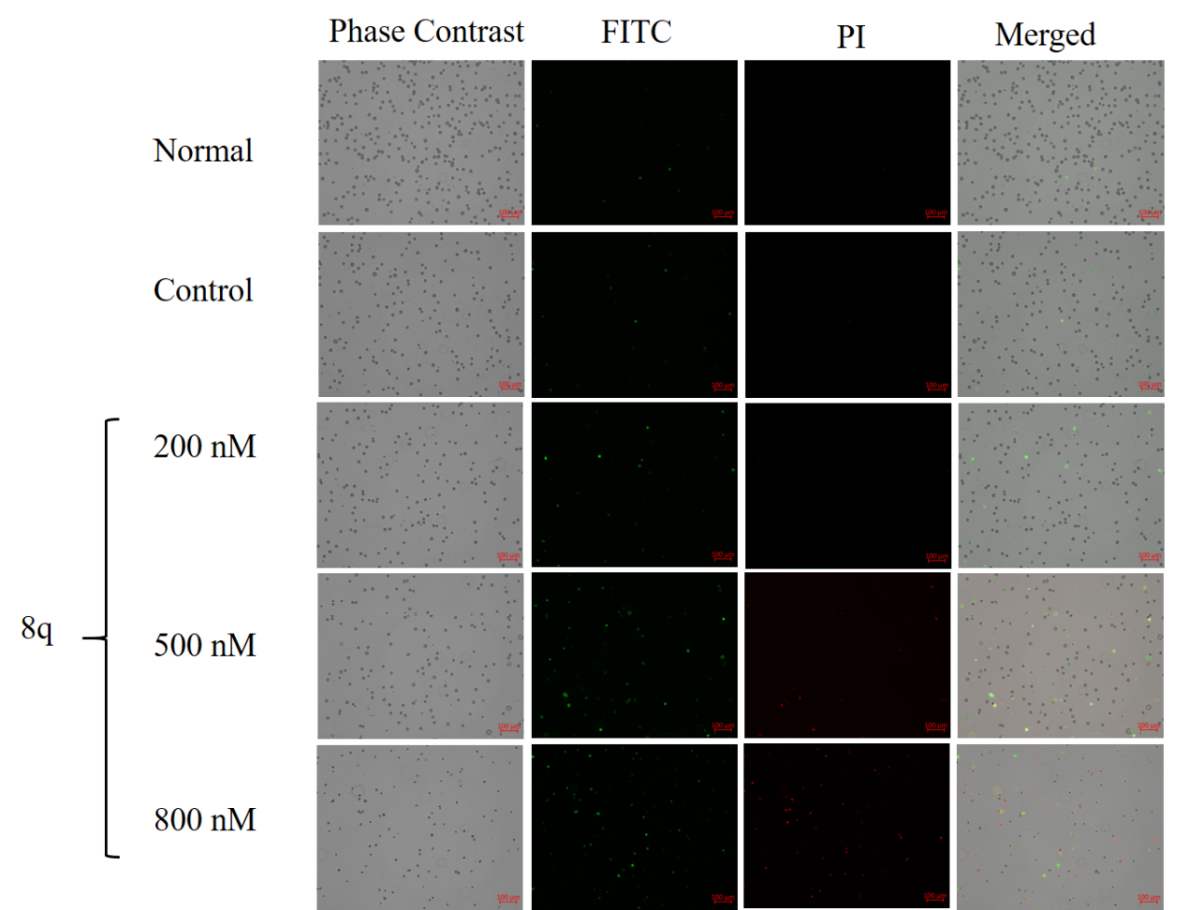


**Figure S3**. **8q** induced apoptosis in K562 cells at different concentrations (48h). Annexin V-FITC shows early apoptotic cells with green fluorescence. PI shows late apoptotic and necrotic cells with red fluorescence.

**
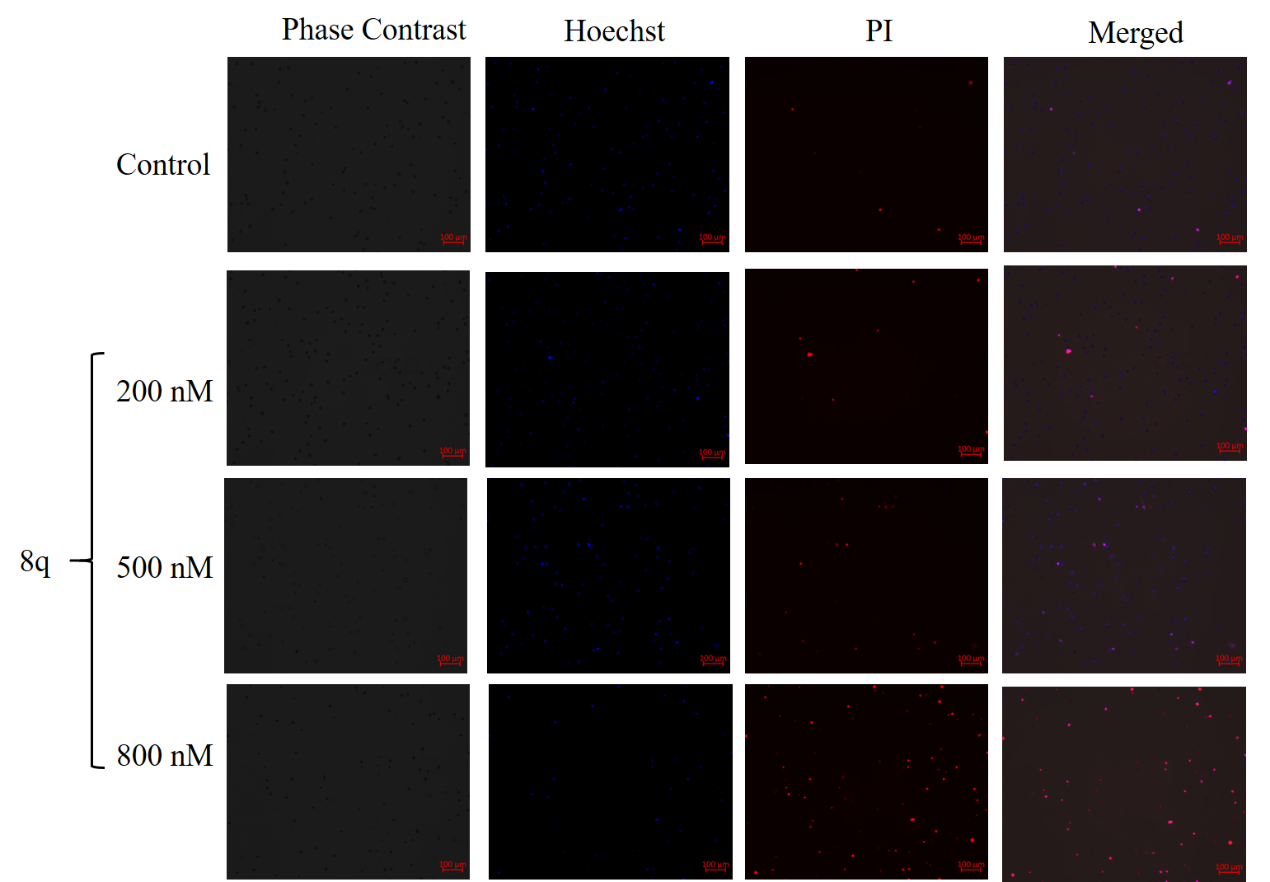
**

**Figure S4**. **8q** induced apoptosis and necrosis in K562 cells with different concentrations (48h). Hoechst 33342 can recognize apoptotic cells and show blue fluorescence. PI is used to identify necrotic cells, showing red fluorescence.

**Table S1. MS/MS spectrogram matched of positive ion mode**

| **Detected MS/MS** | **Reference MS/MS** |
| --- | --- |
|  | 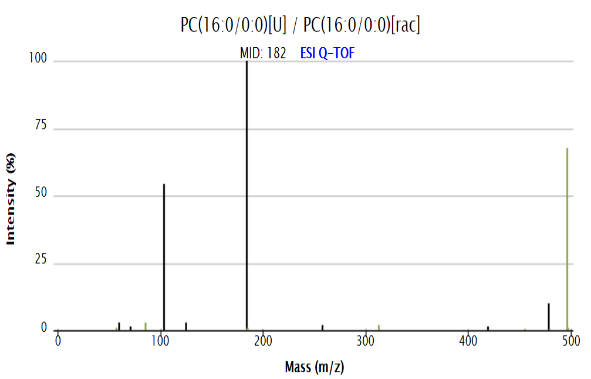 |
|  | 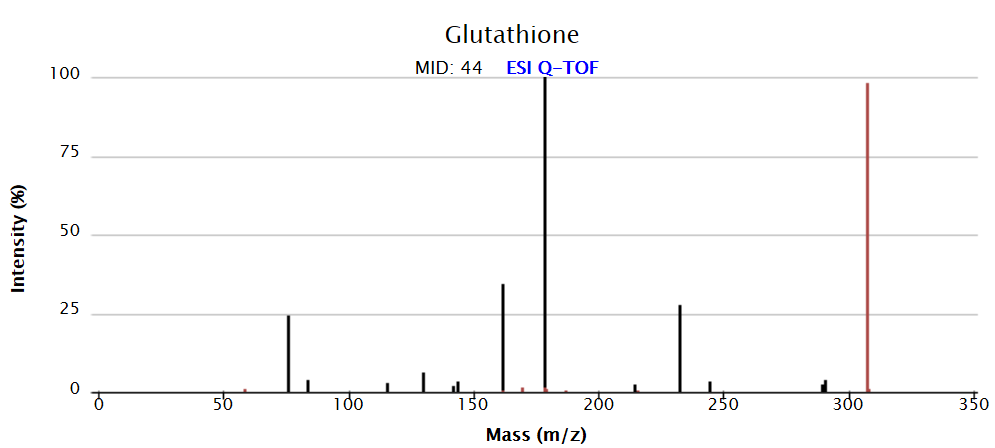 |
|  | 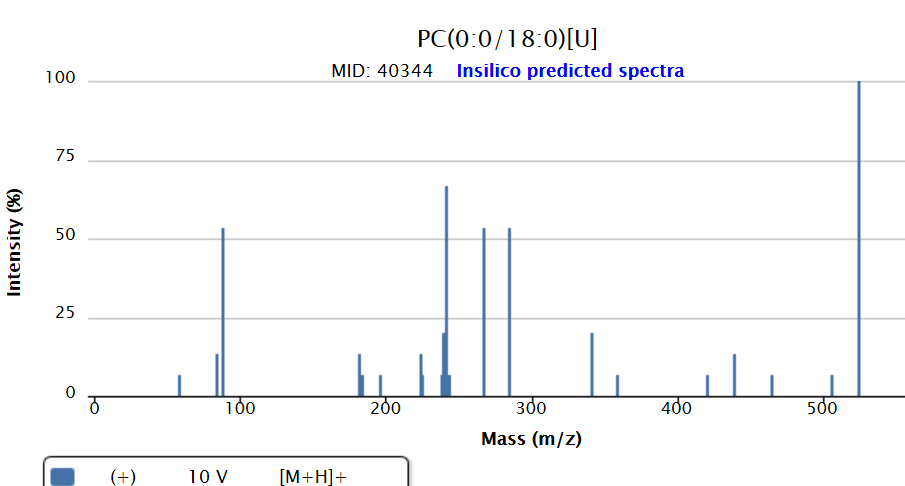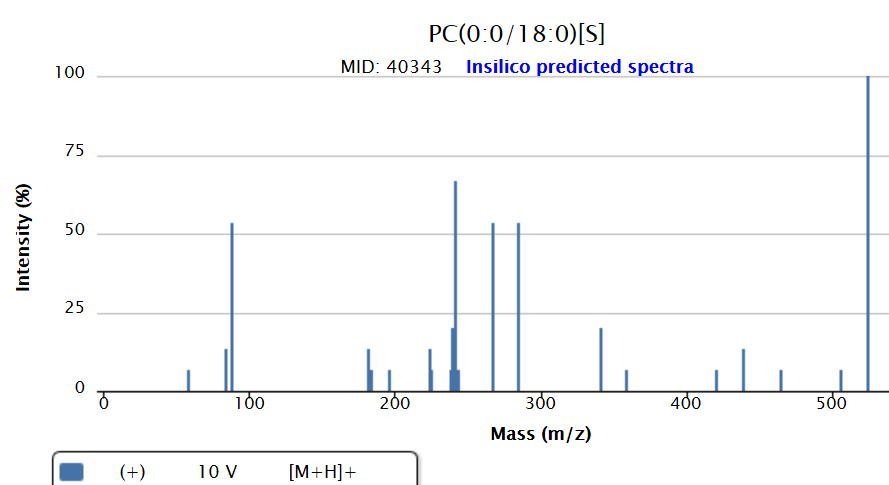 |
|  | 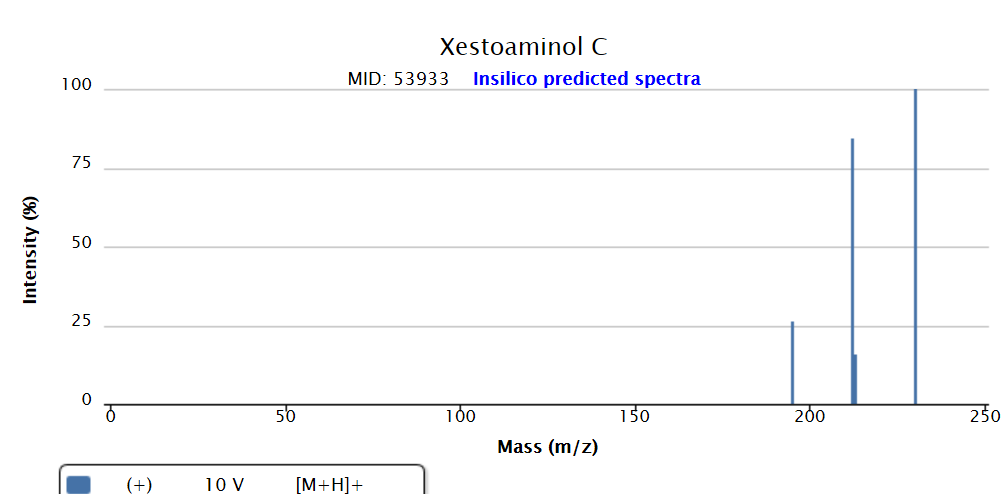 |
|  | 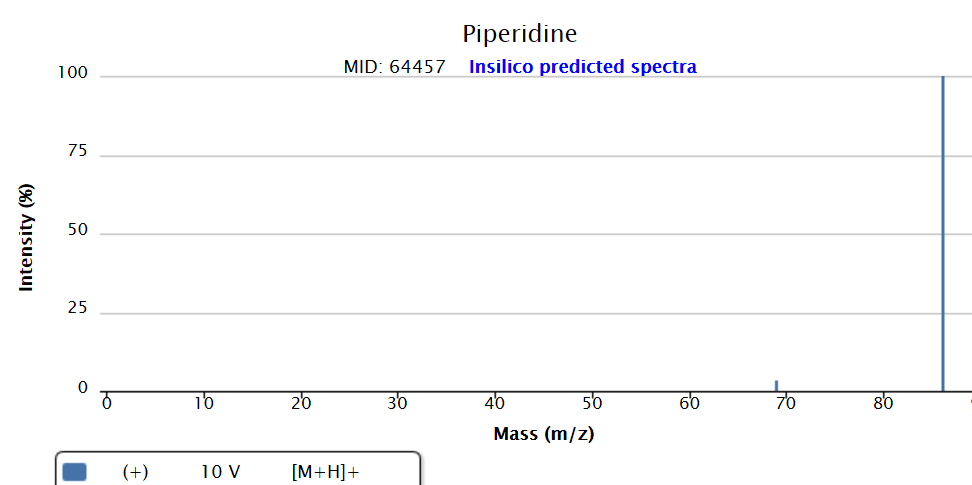 |
|  | 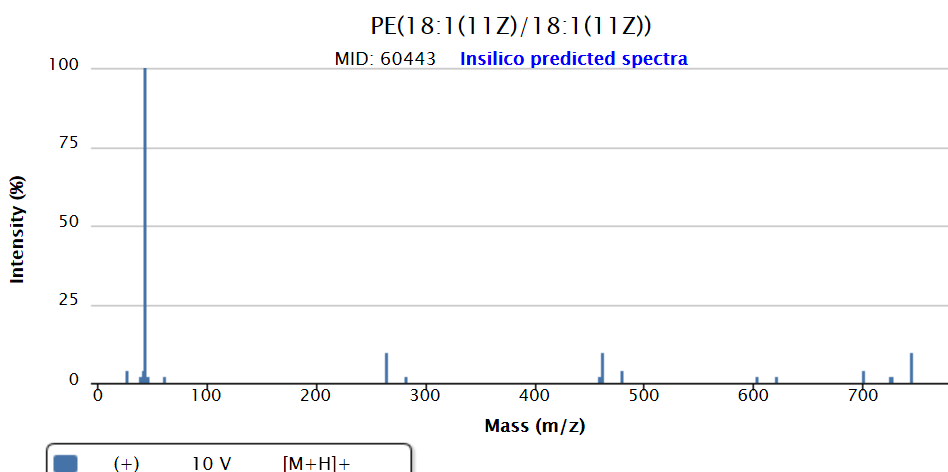 |
|  | 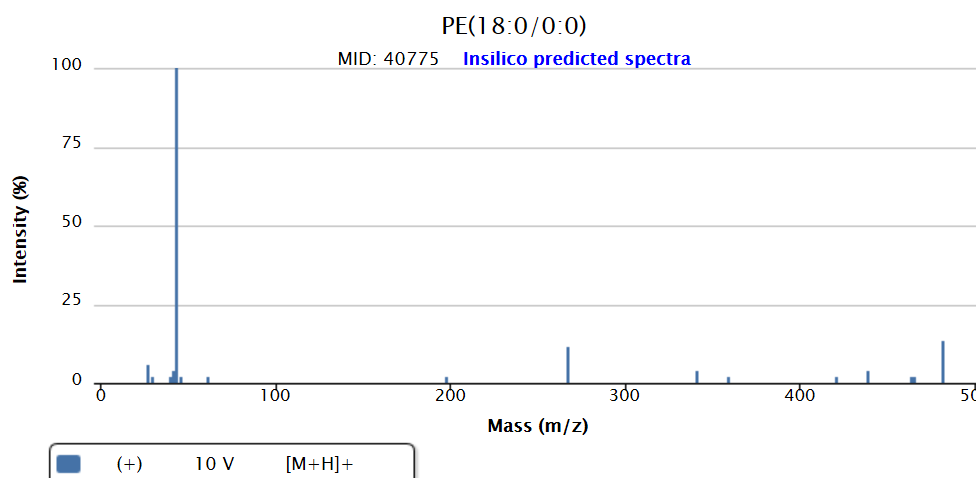 |
|  | 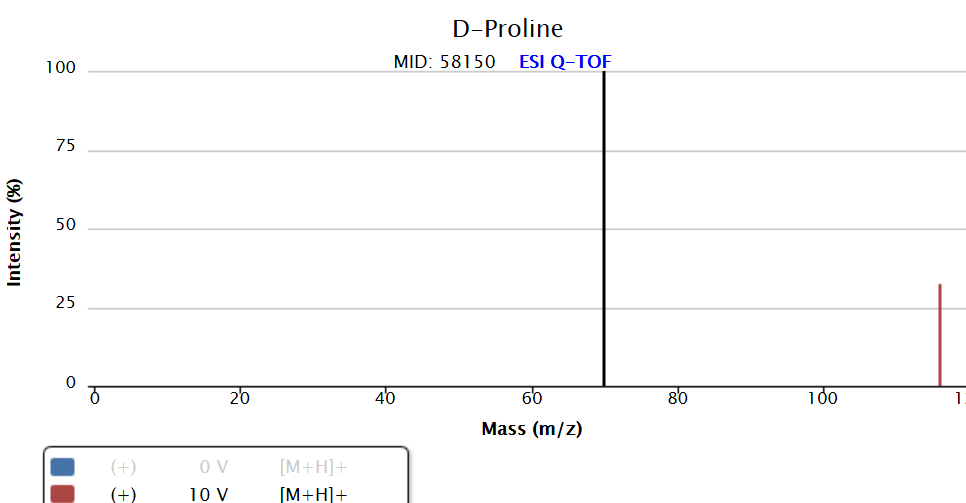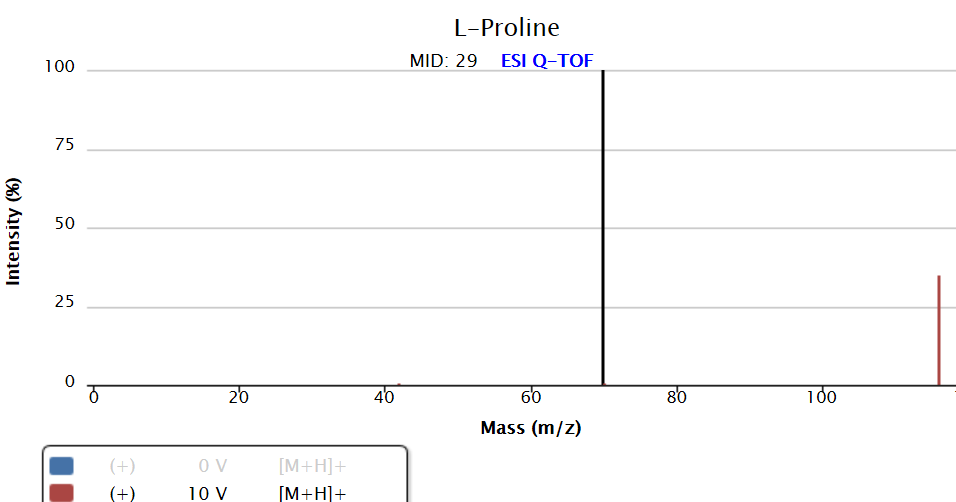 |
|  | 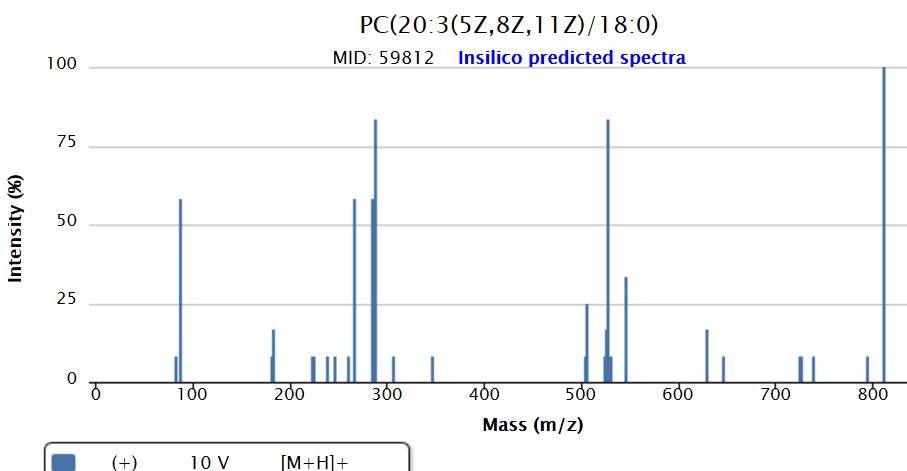 |
|  | 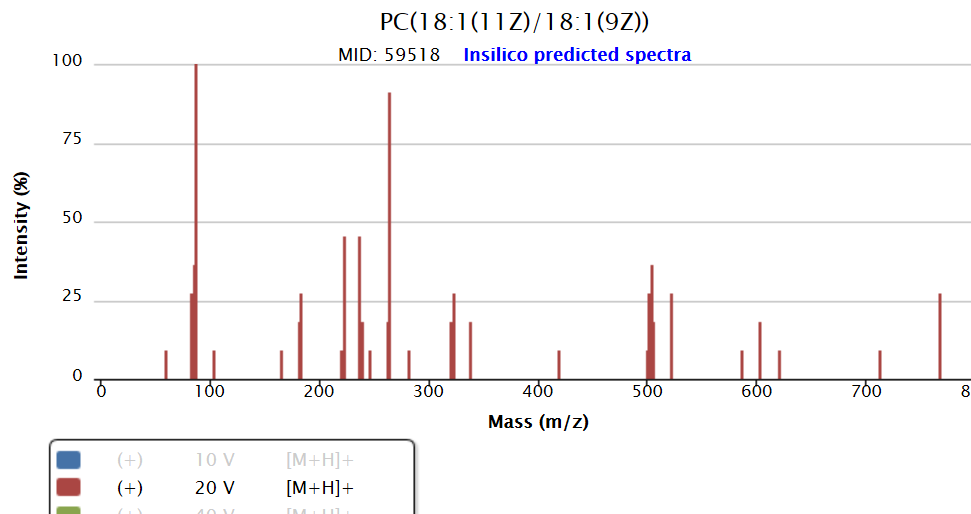 |
|  | 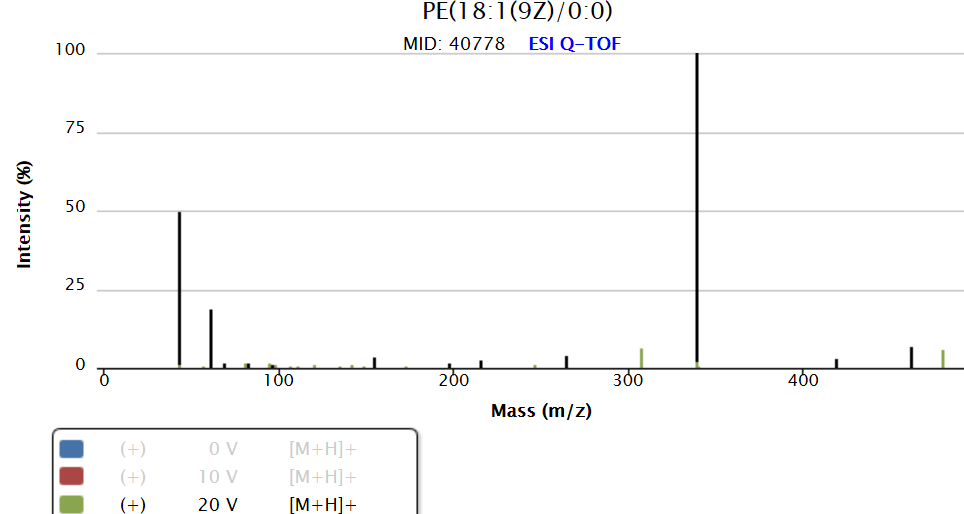 |
|  | 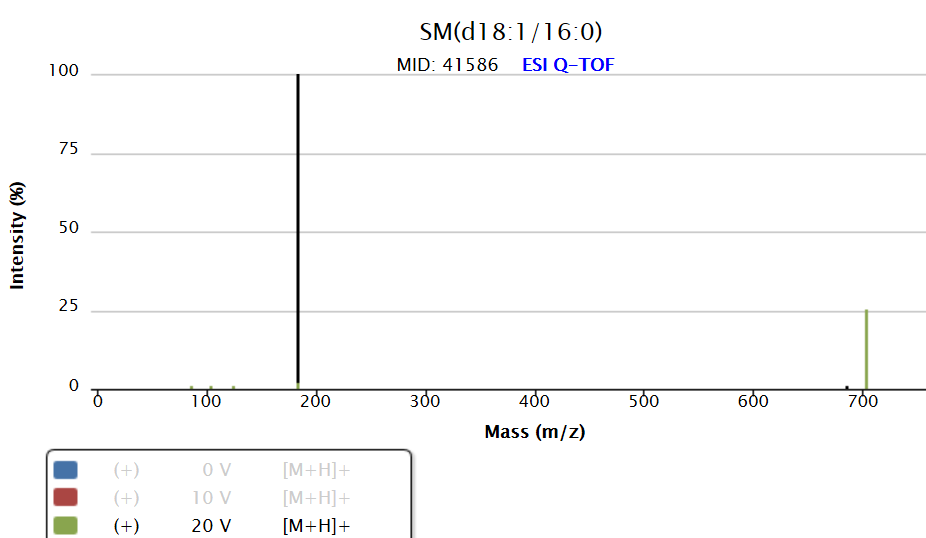 |
|  | 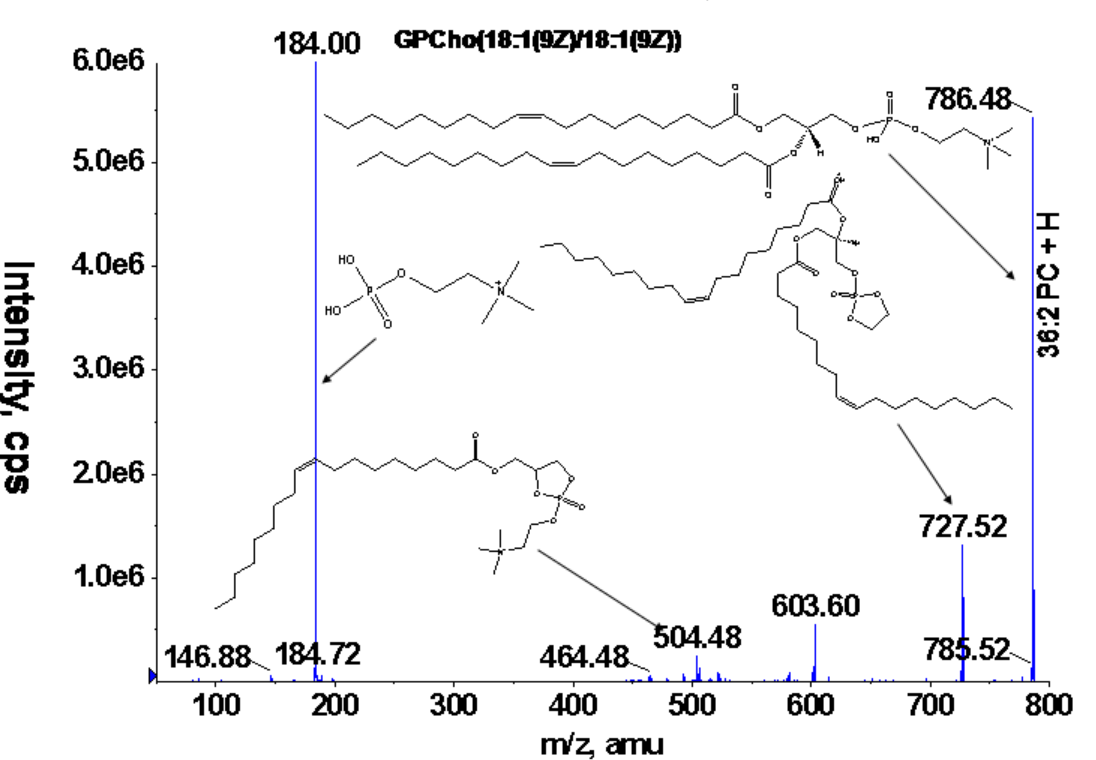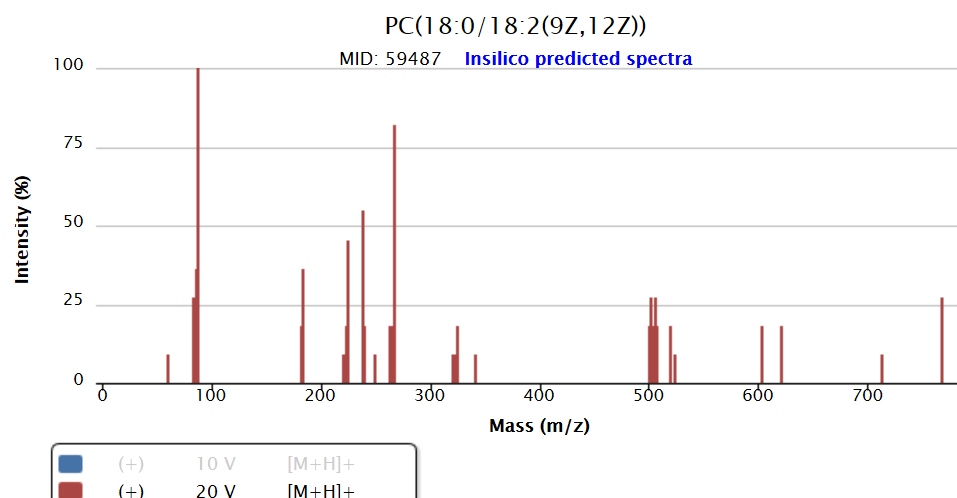 |
|  | 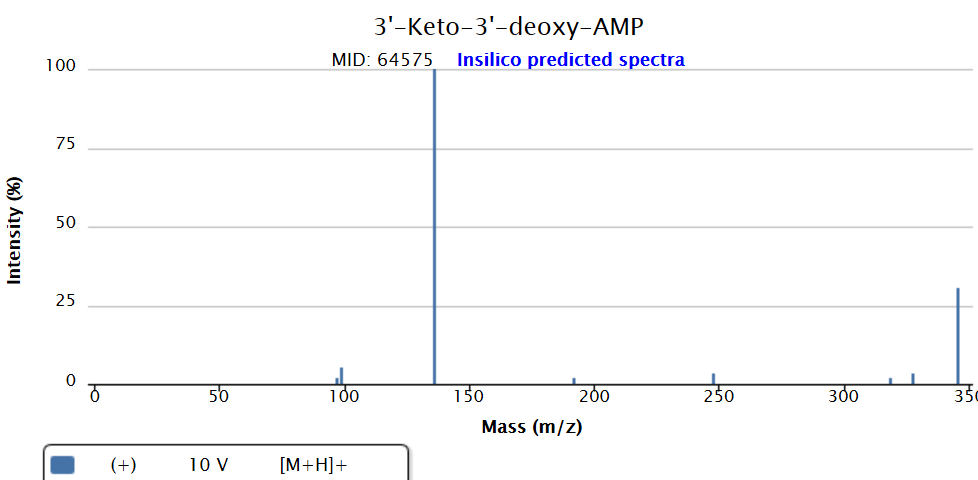 |
|  | 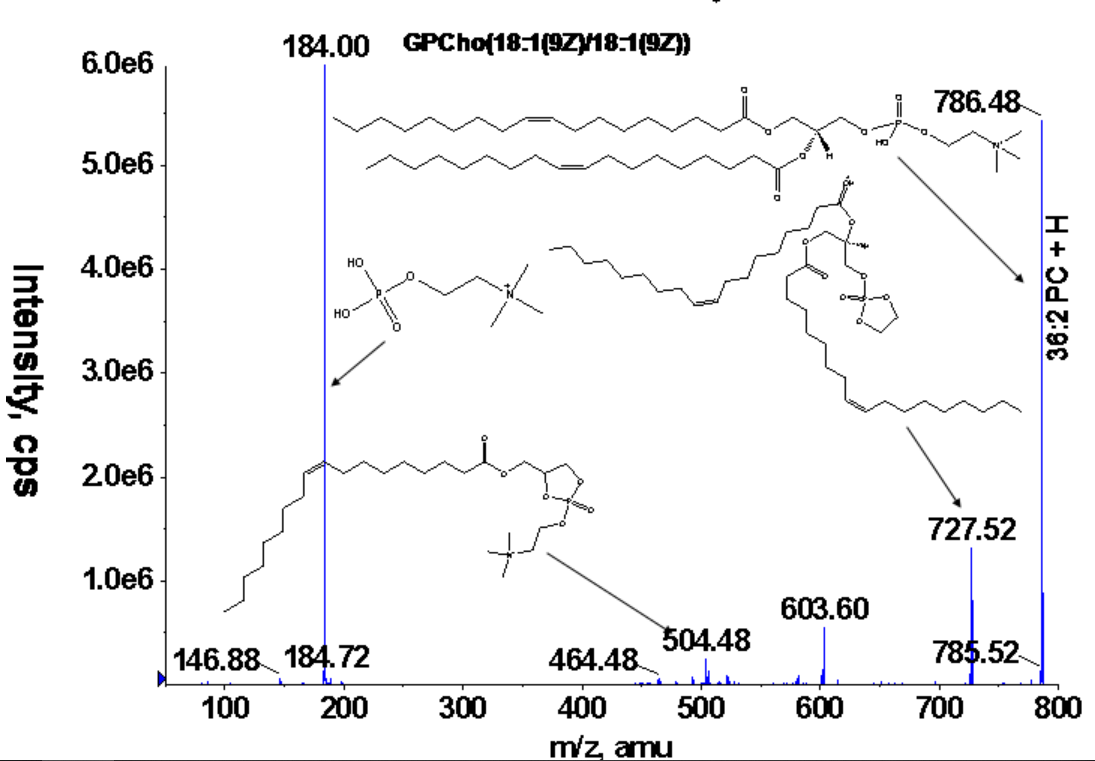 |
|  | 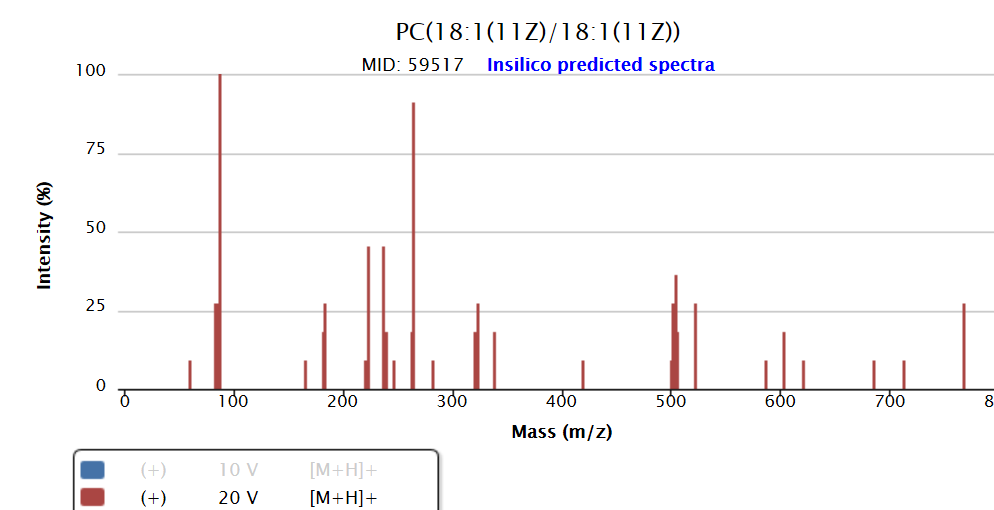 |
|  | 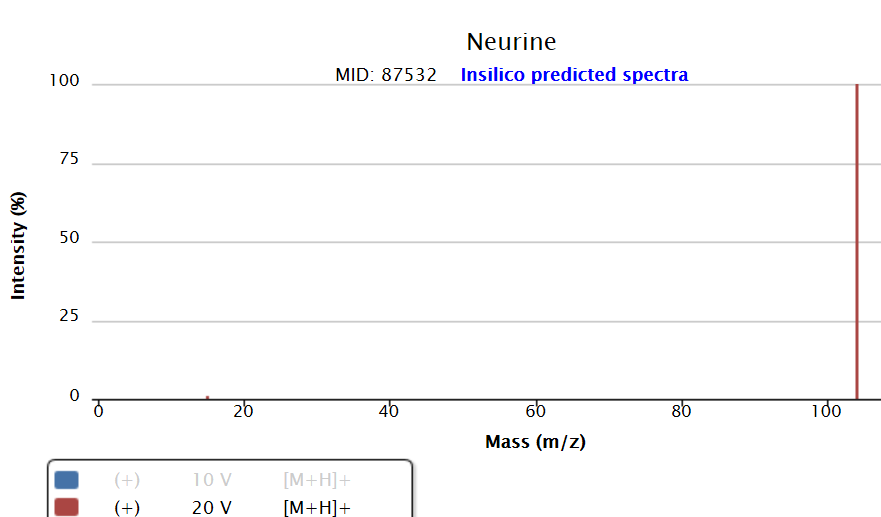 |
|  | 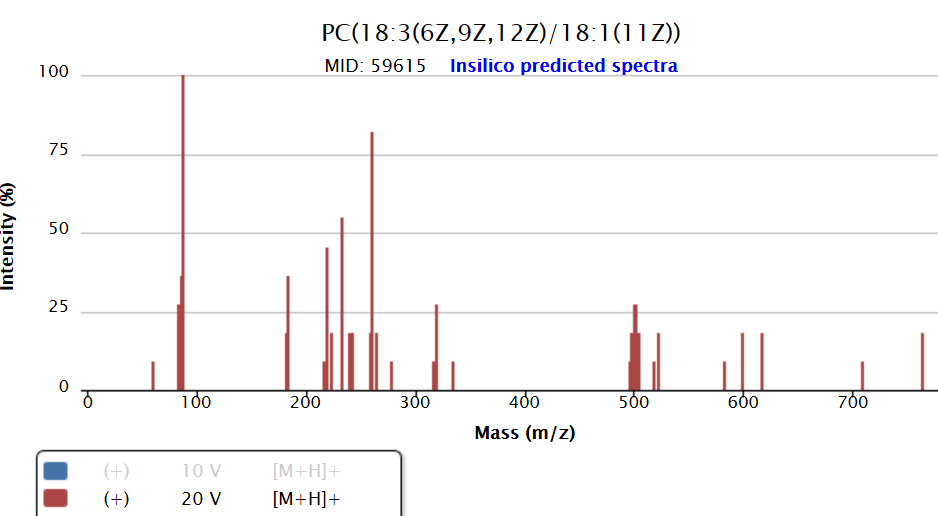 |
|  | 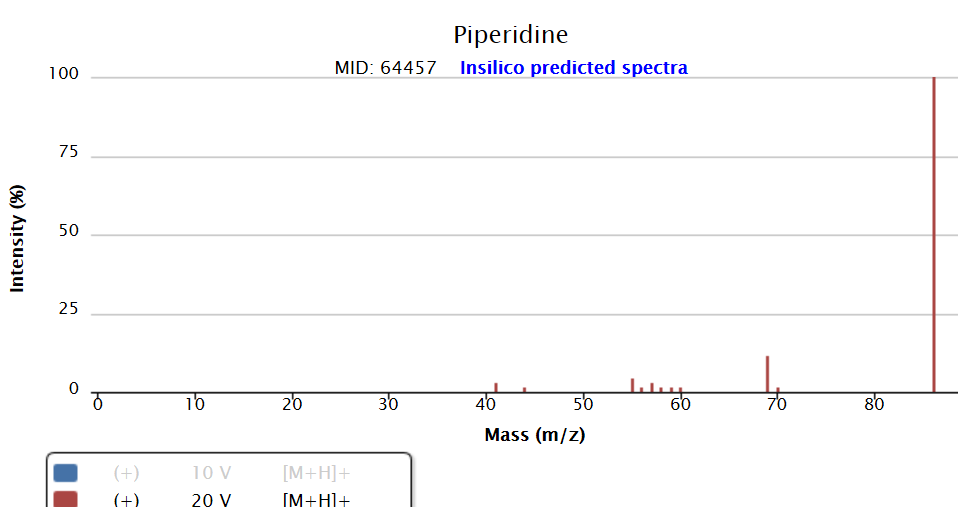 |
|  | 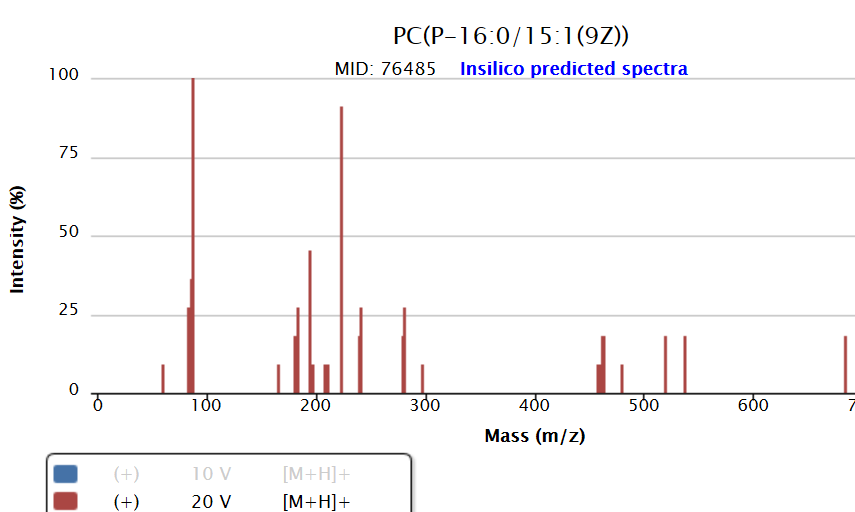 |
|  | 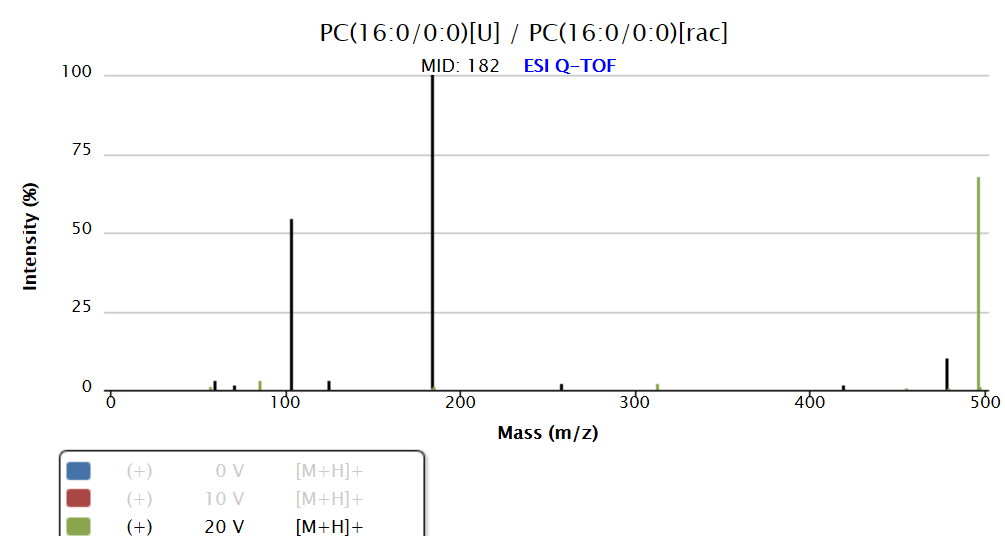 |
|  | 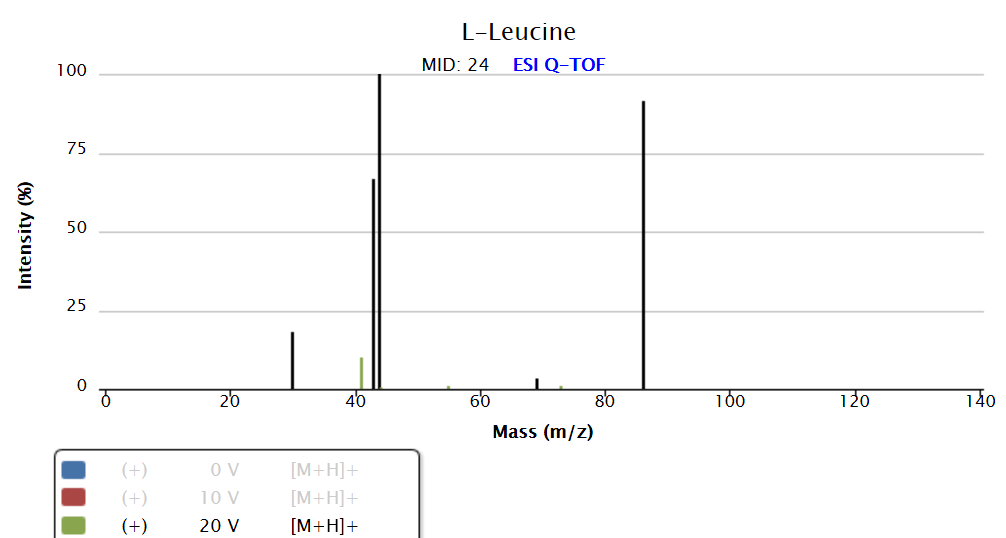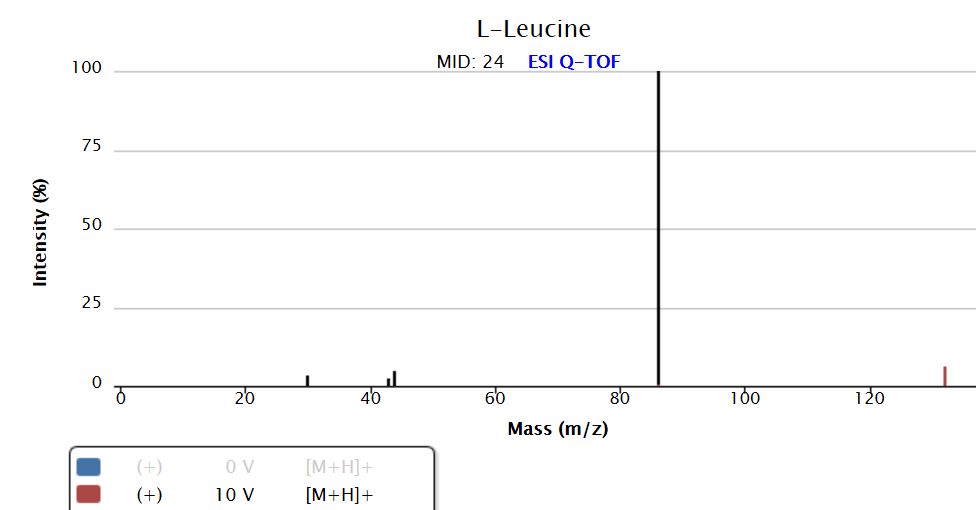 |
|  | 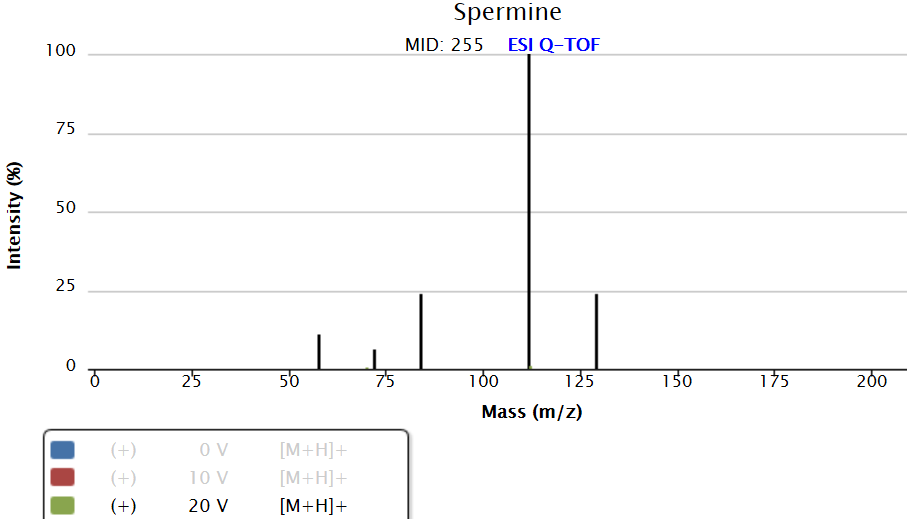 |
|  | 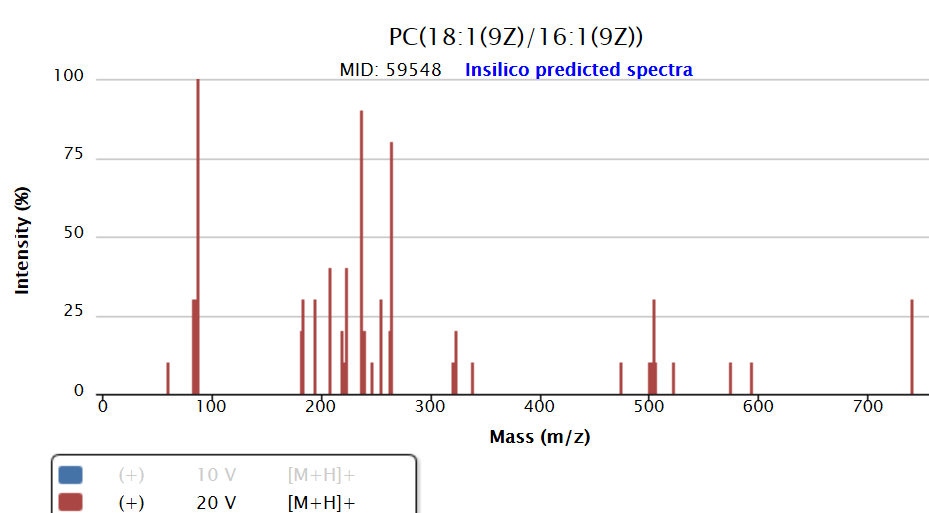 |
|  | 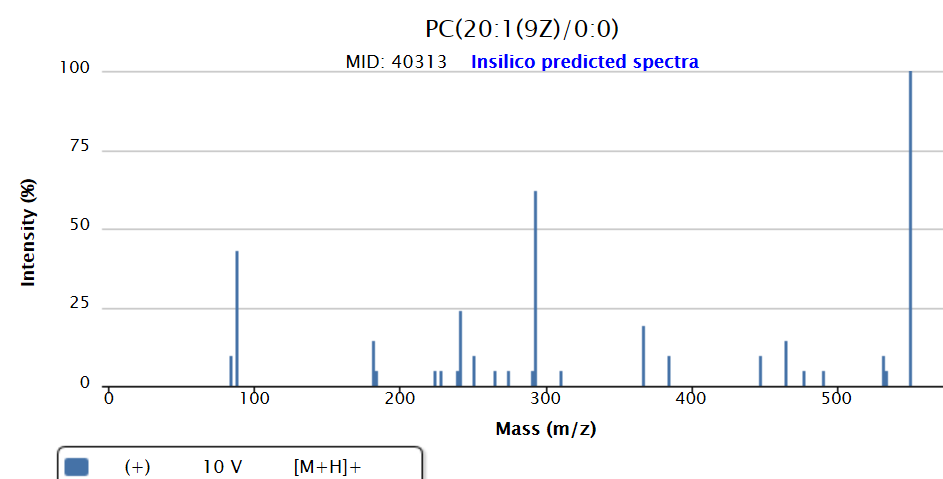 |
|  | 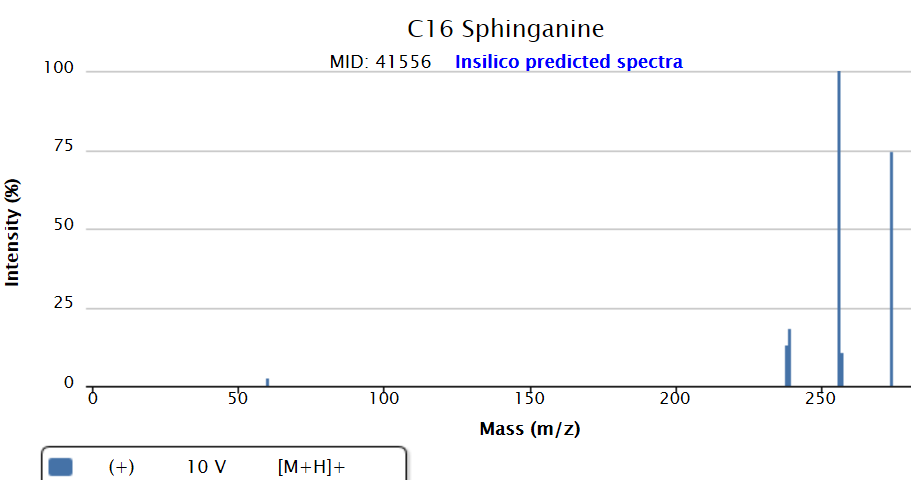 |
|  | 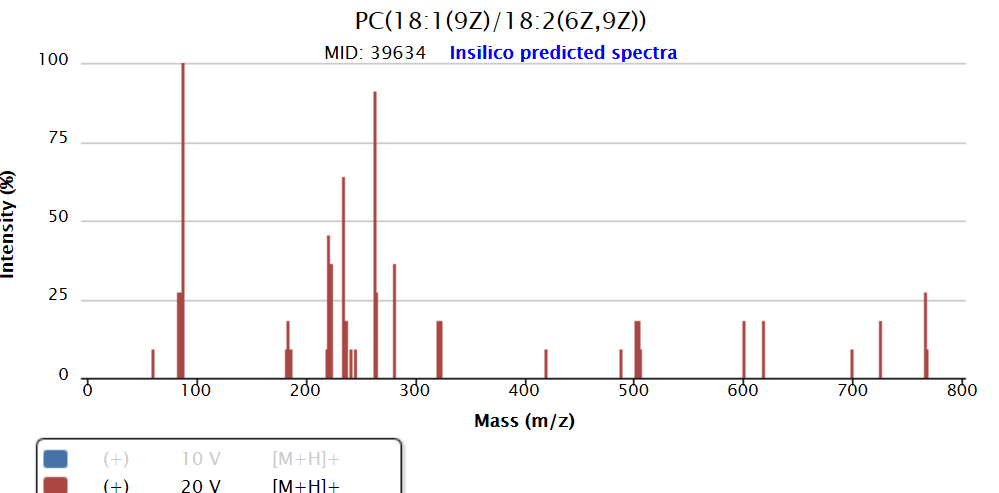 |
|  | 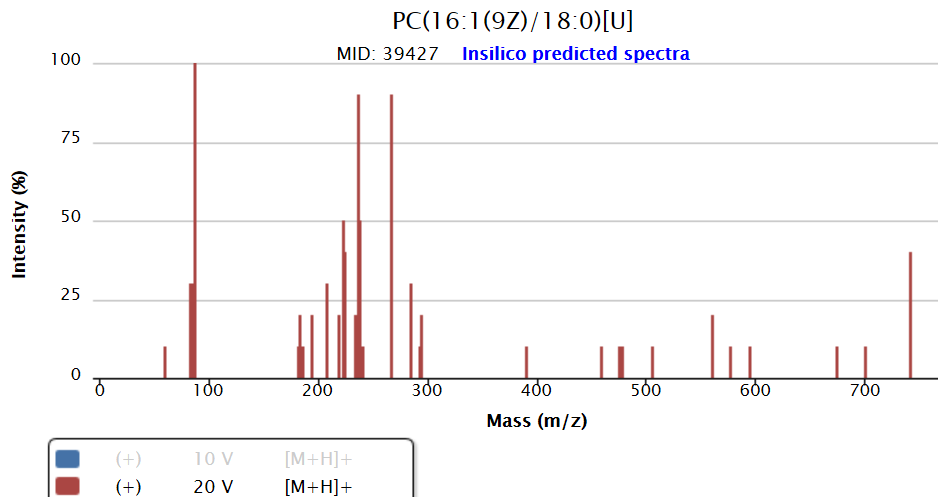 |
|  | 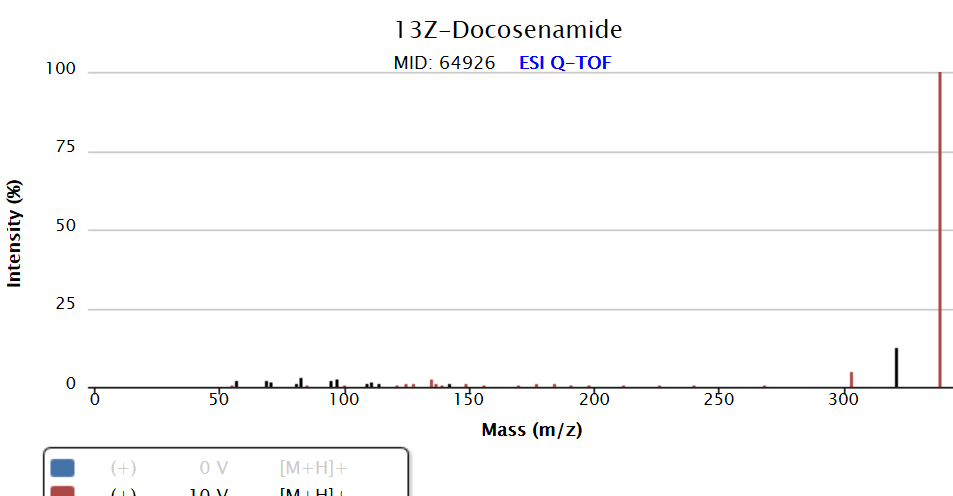 |
|  | 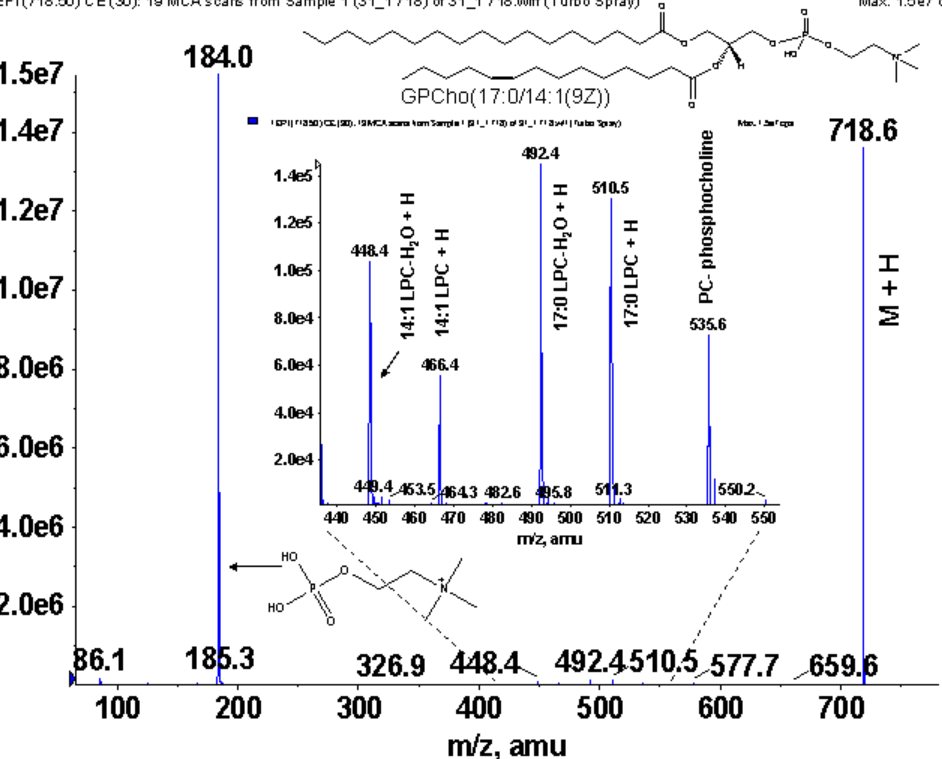 |
|  | 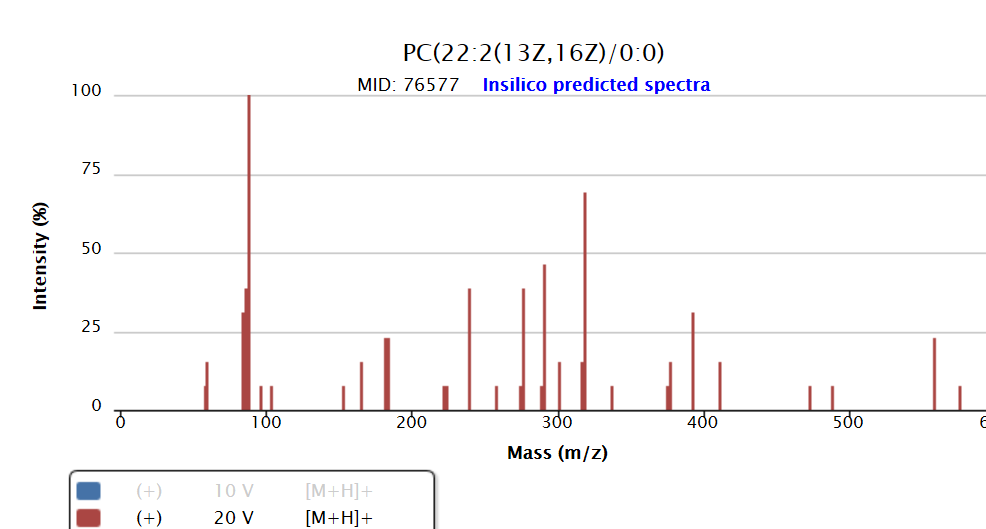 |
|  | 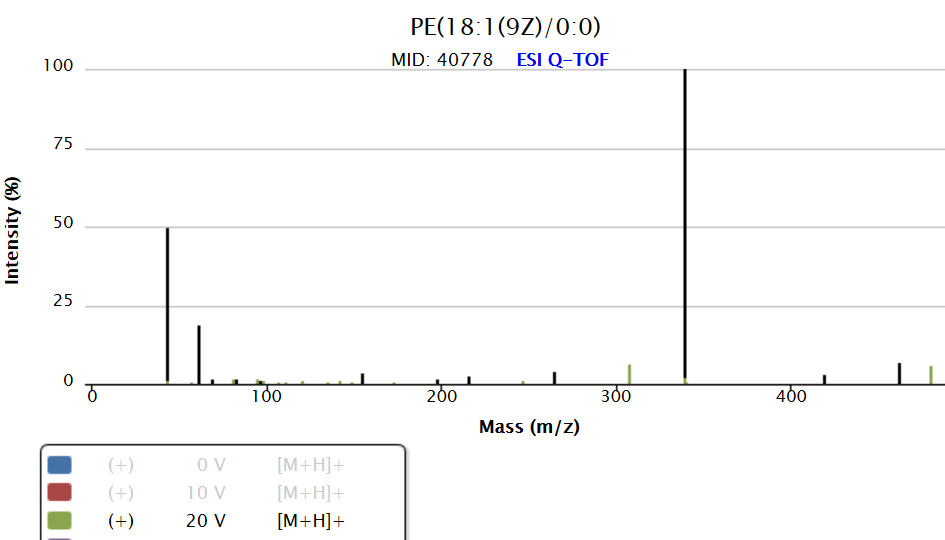 |
|  | 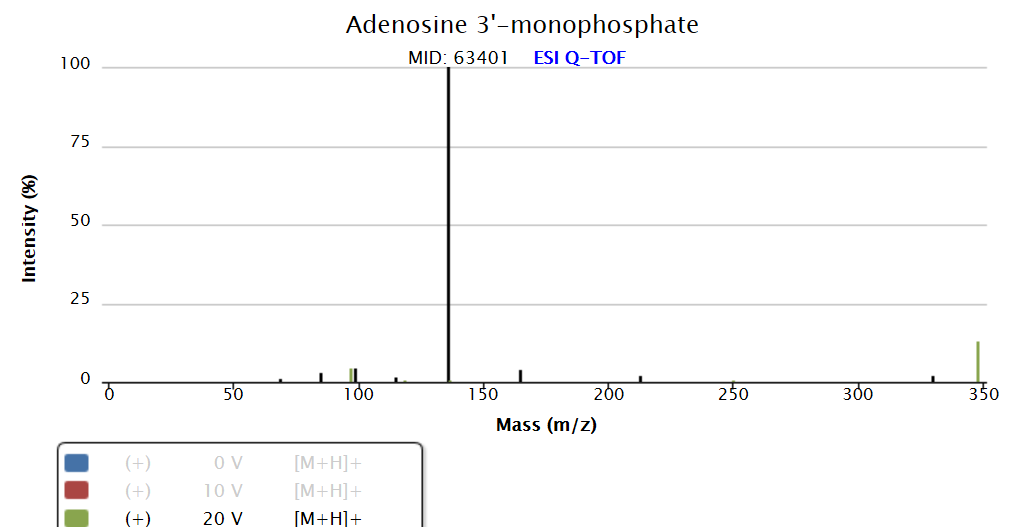 |

**Table S2. MS/MS spectrogram matched of negative ion mode**

| **Detected MS/MS** | **Reference MS/MS** |
| --- | --- |
|    | 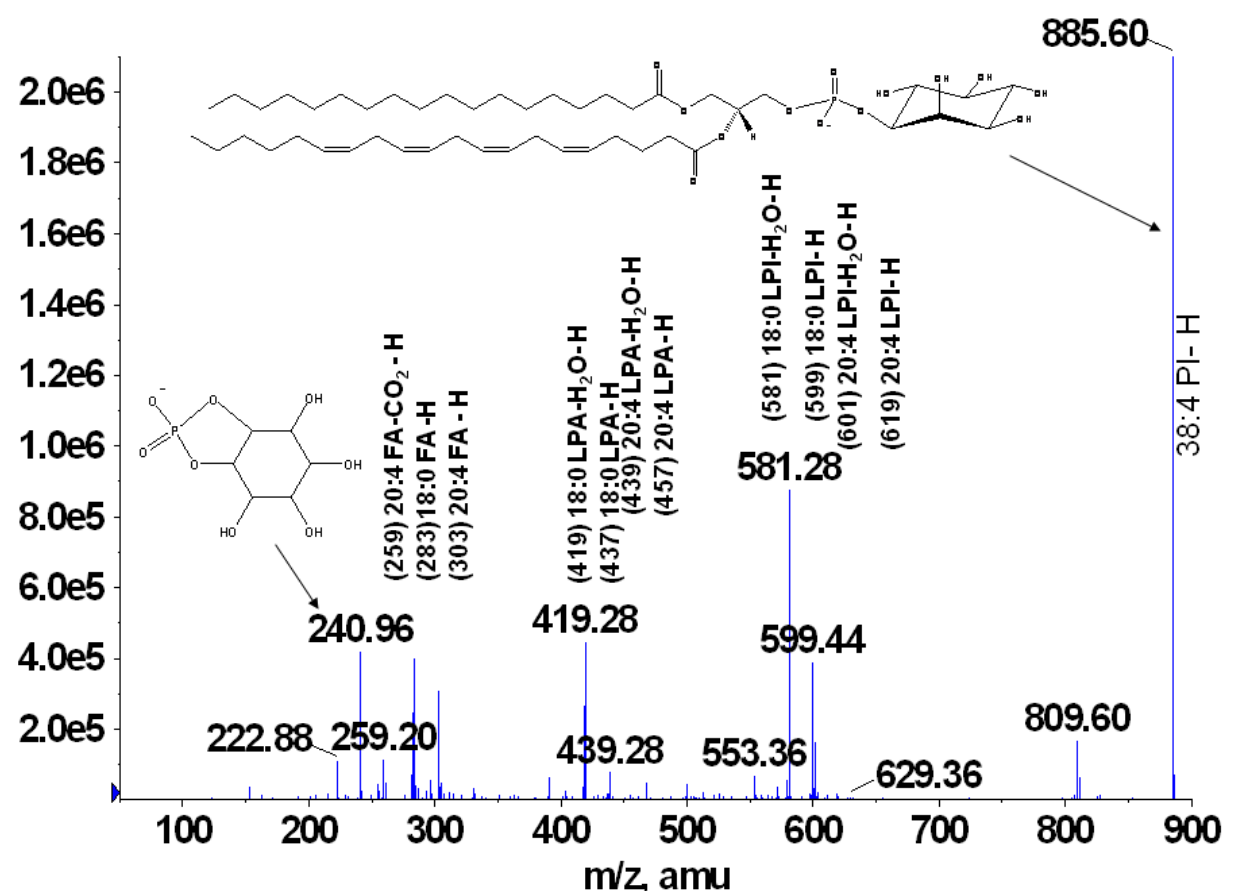 |
|    | 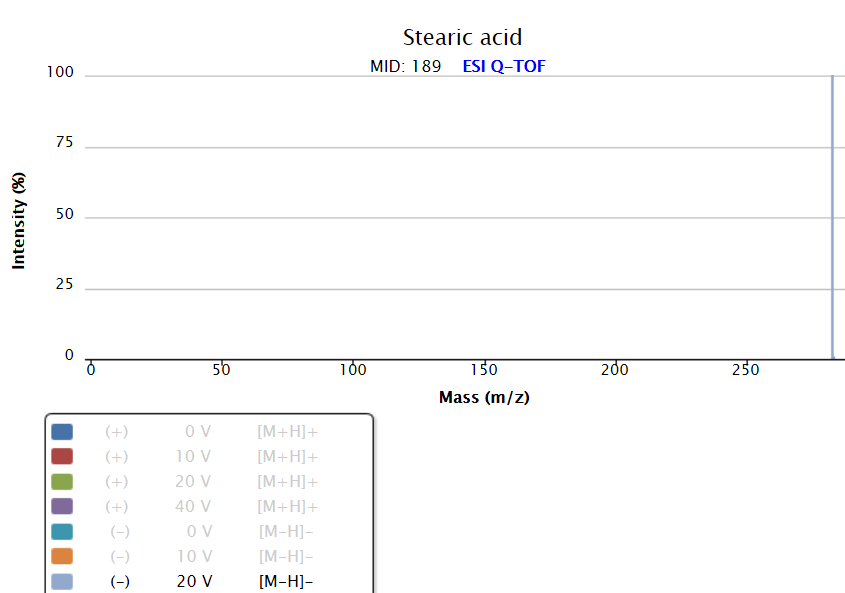  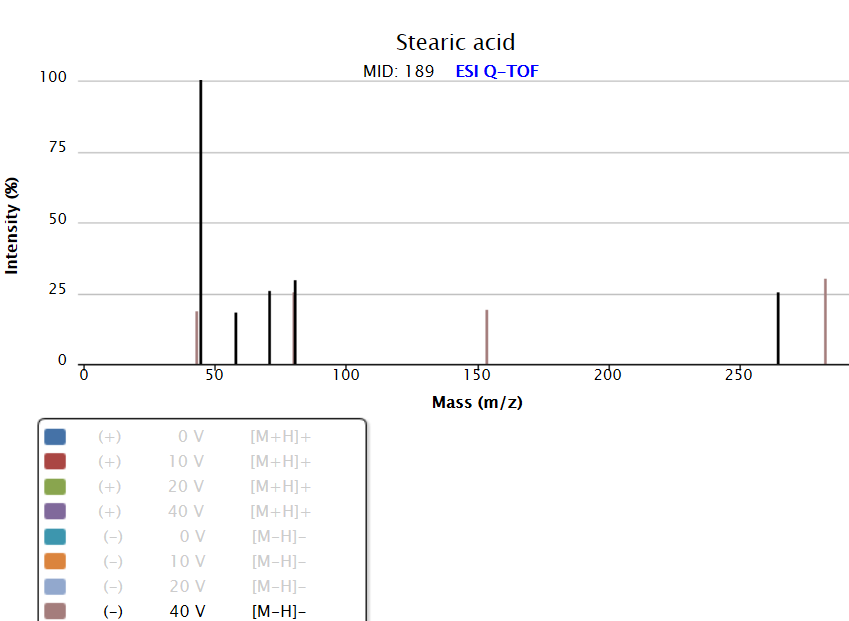 |
|  | 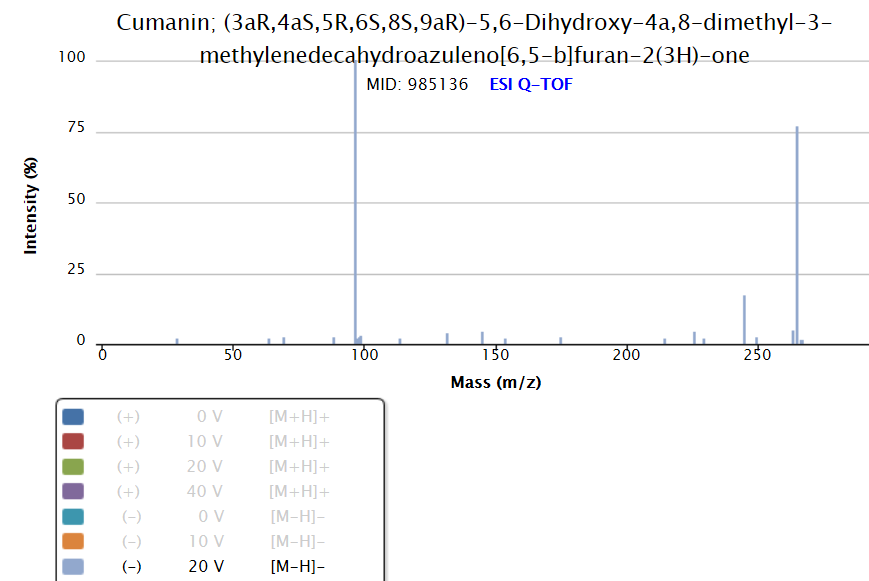 |
|  | 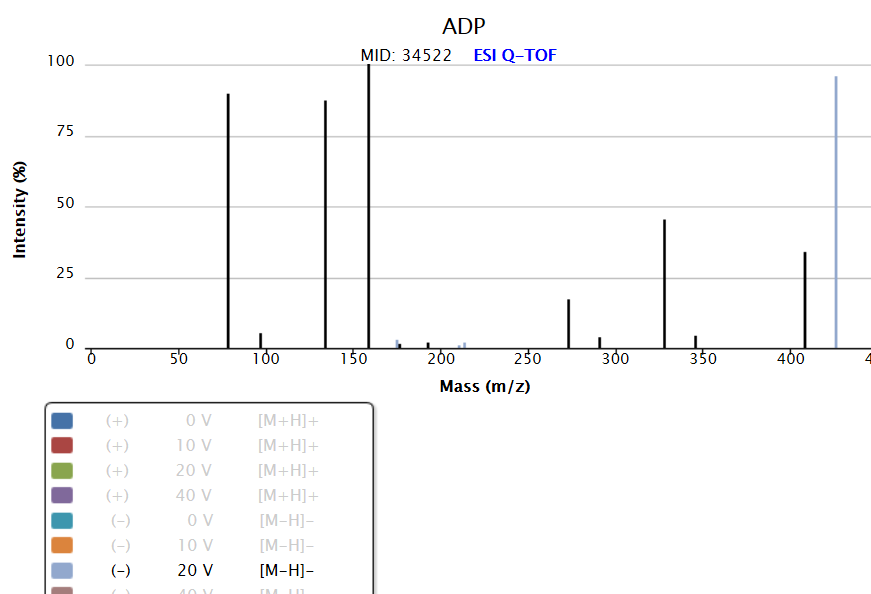 |
|    | 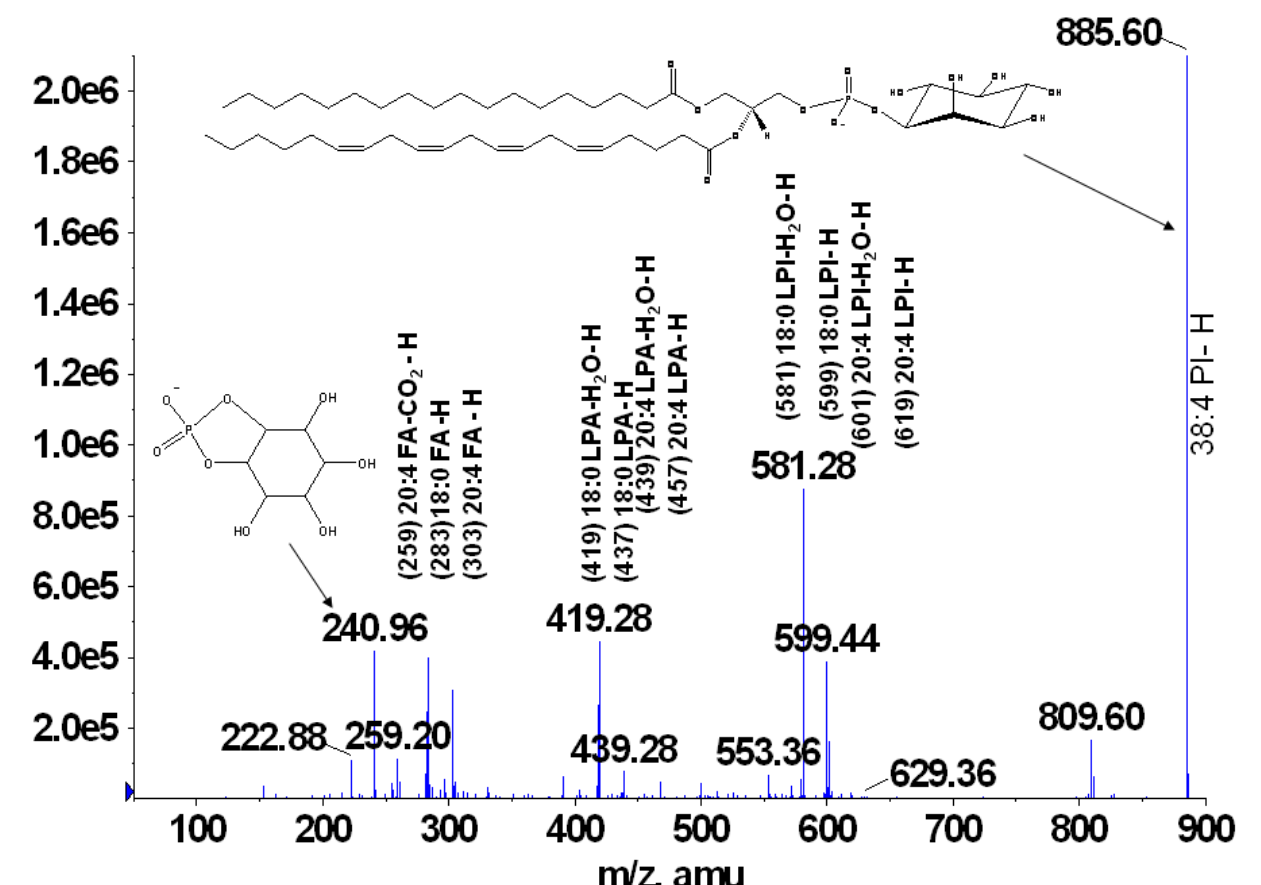 |
|  | 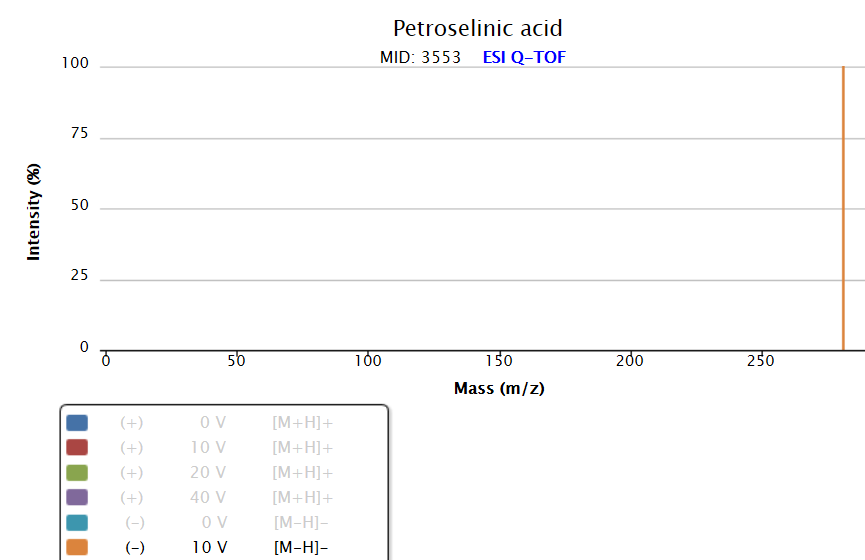  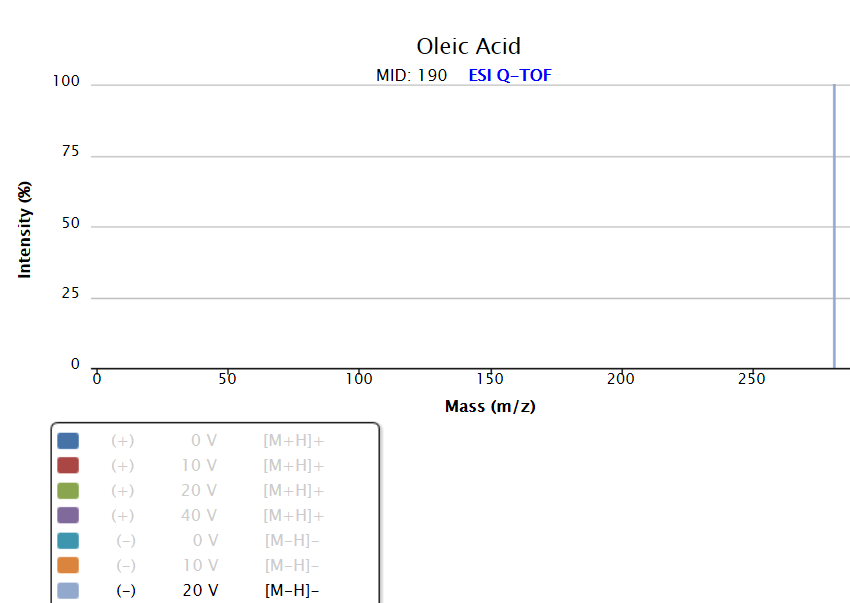 |
|  | 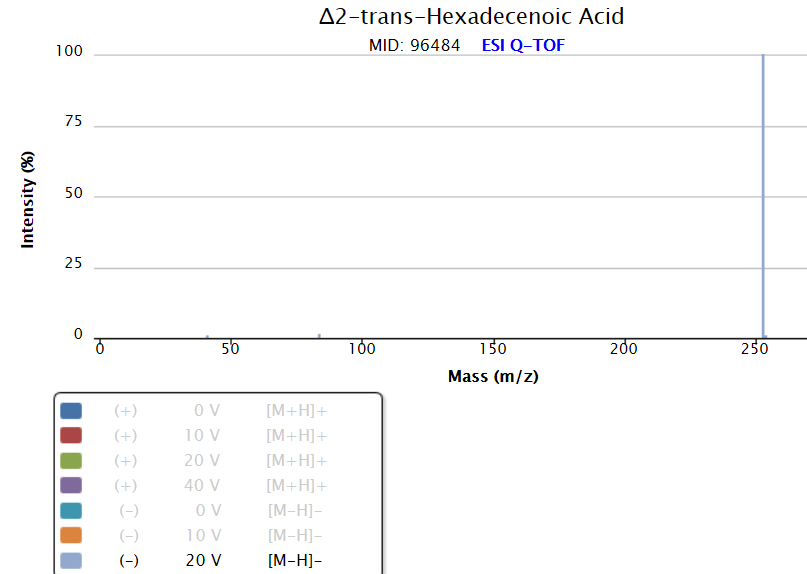 |
|  | 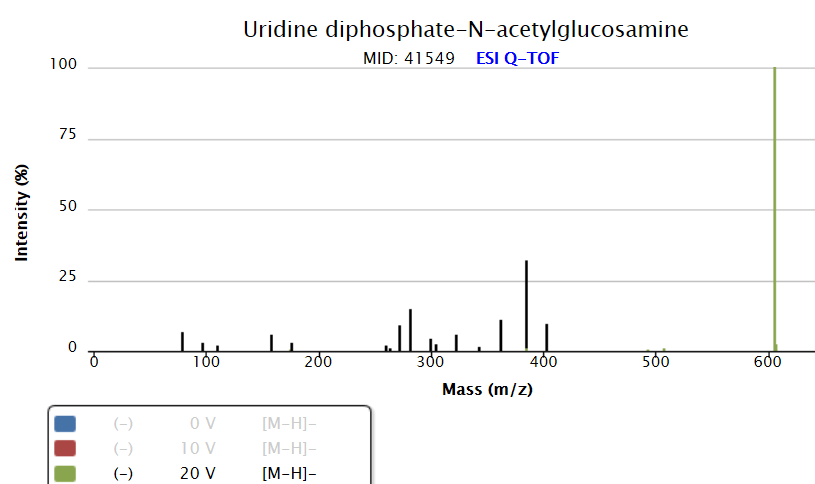 |
|  |  |
|    |  |
|  | 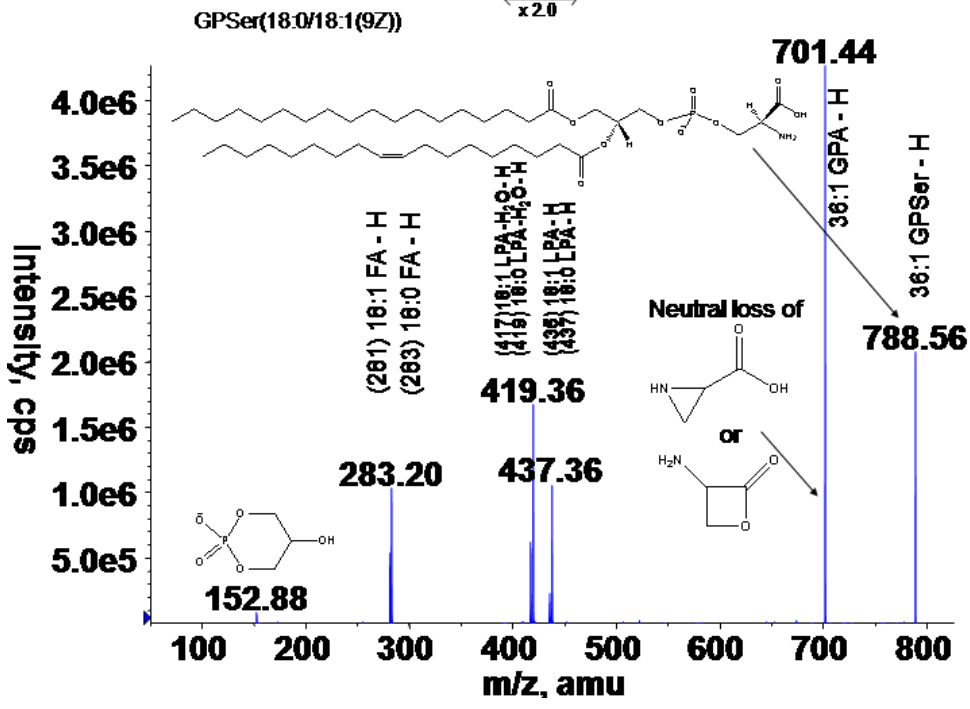 |
|  | 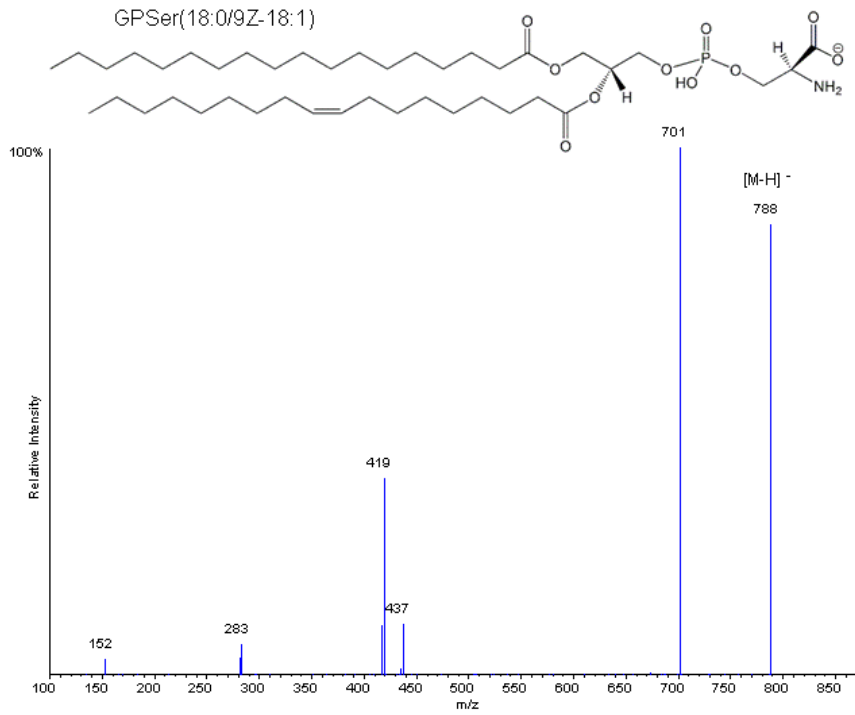 |
|    | 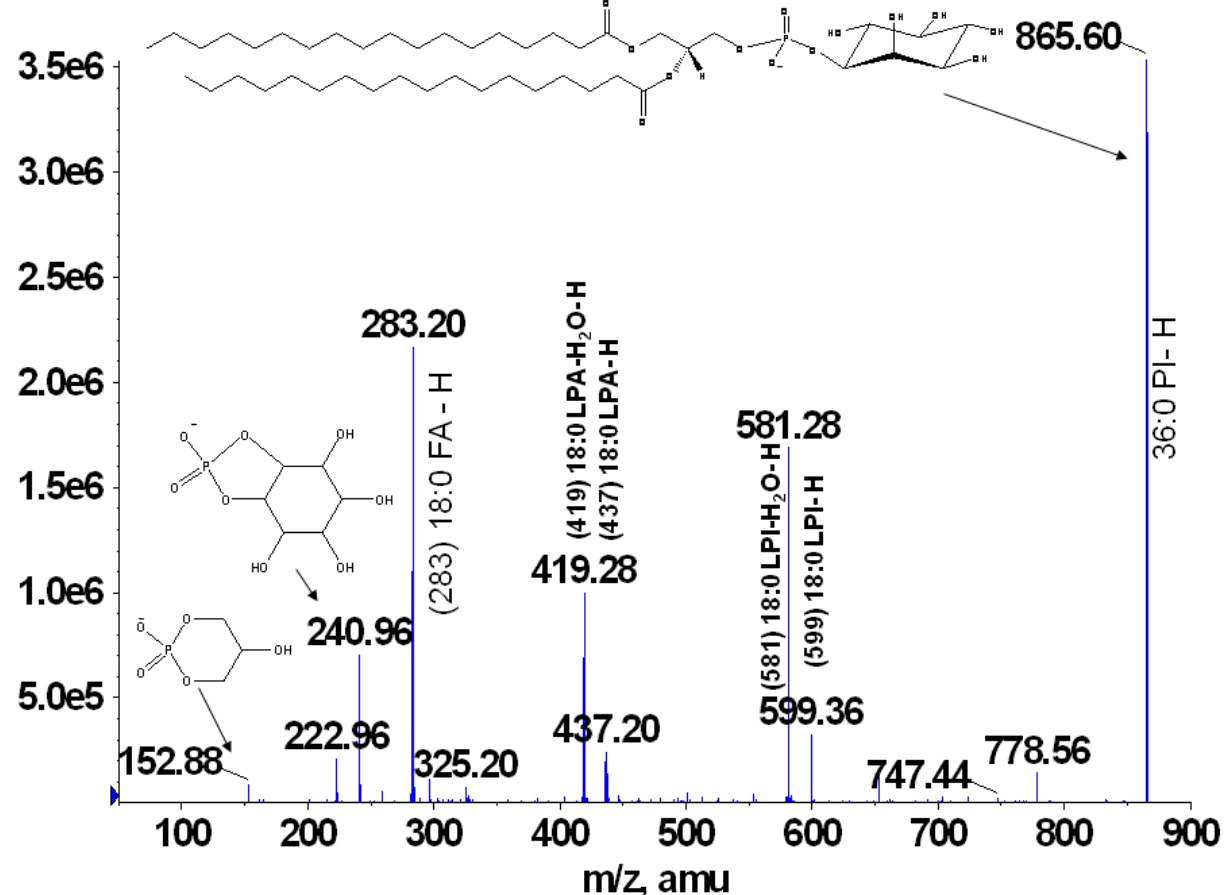 |
|  |  |
|  | 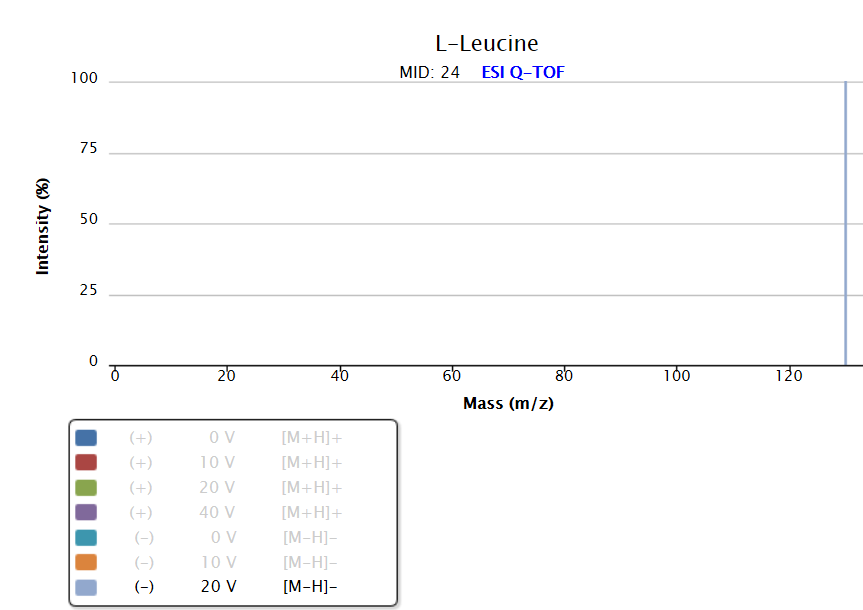 |
|  |  |
|    |  |
|    |  |
|  |  |
|  |  |
|  | 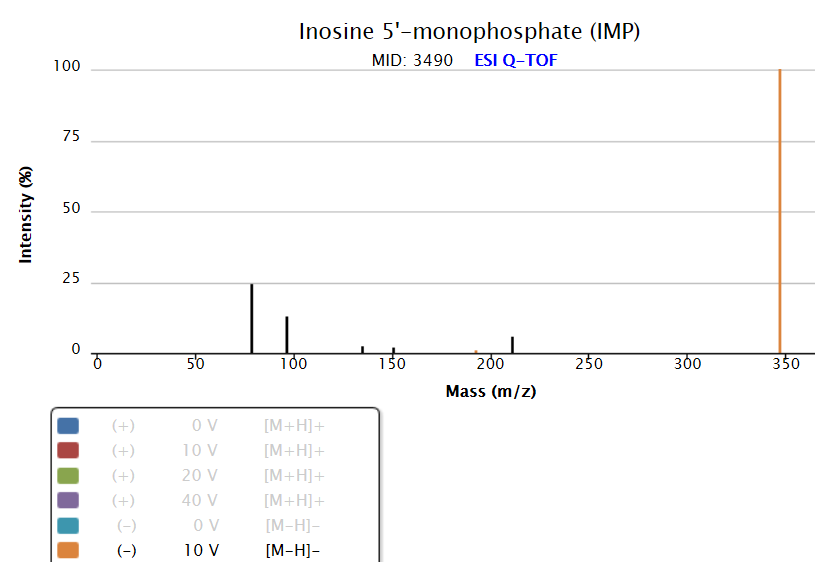 |
|  | 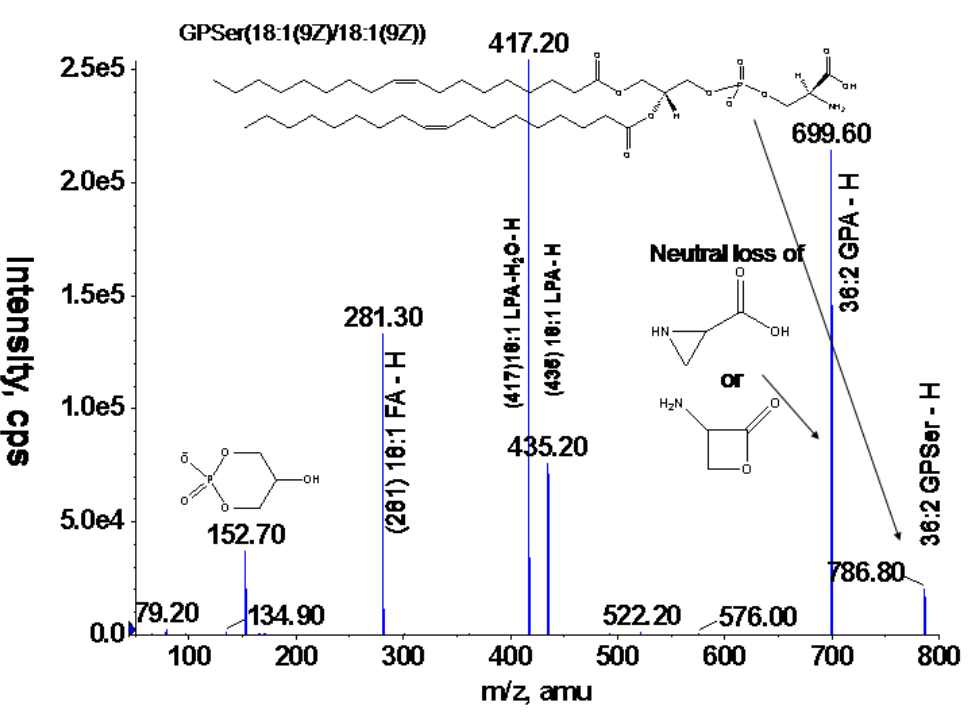 |
|    |  |
|  | 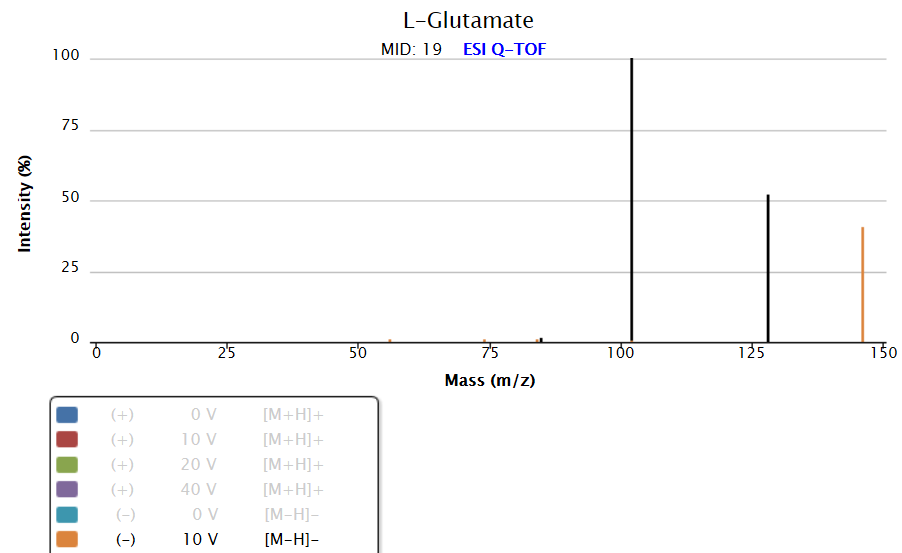 |
|    |  |
|  | 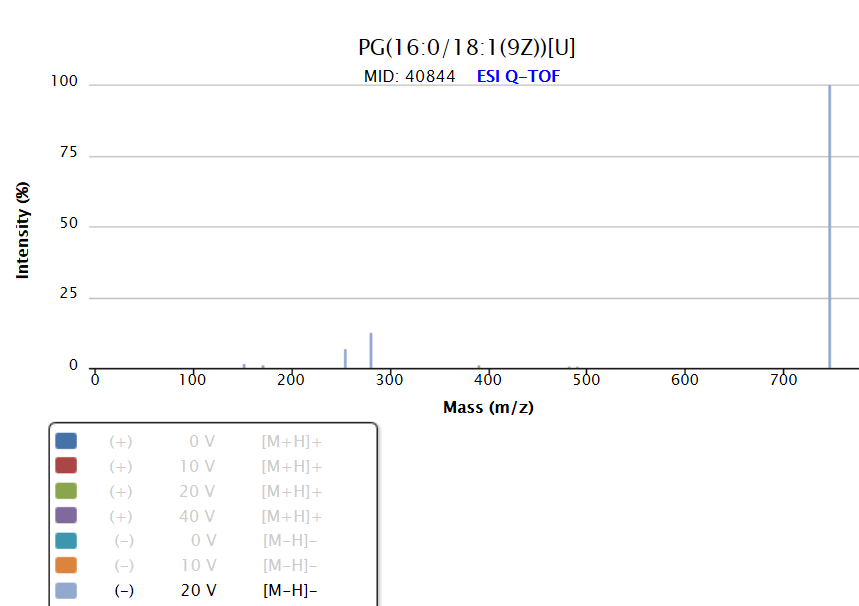 |
|  |  |
|  |  |
|  | 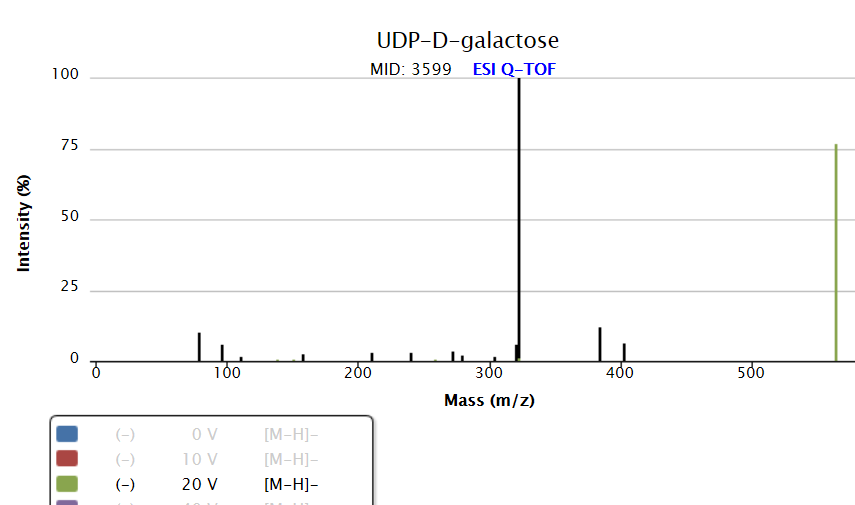 |
|    |  |
|  | 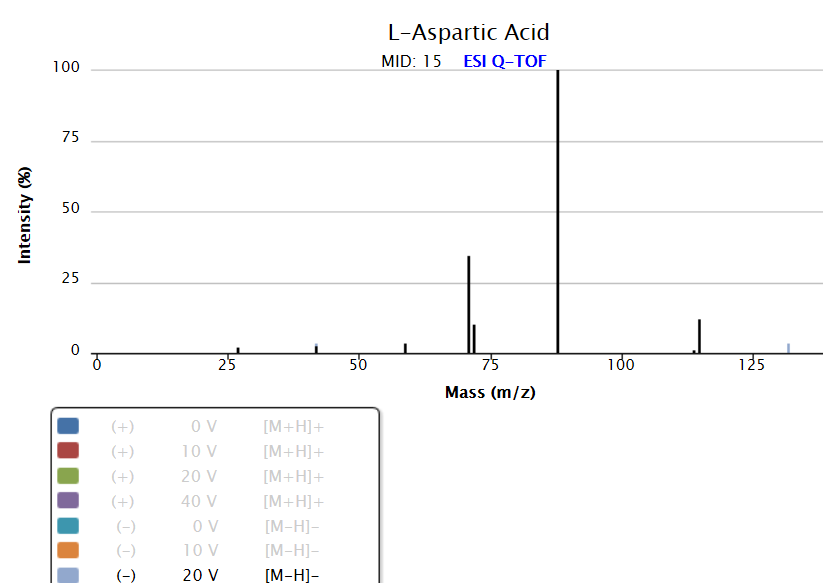 |
|    | 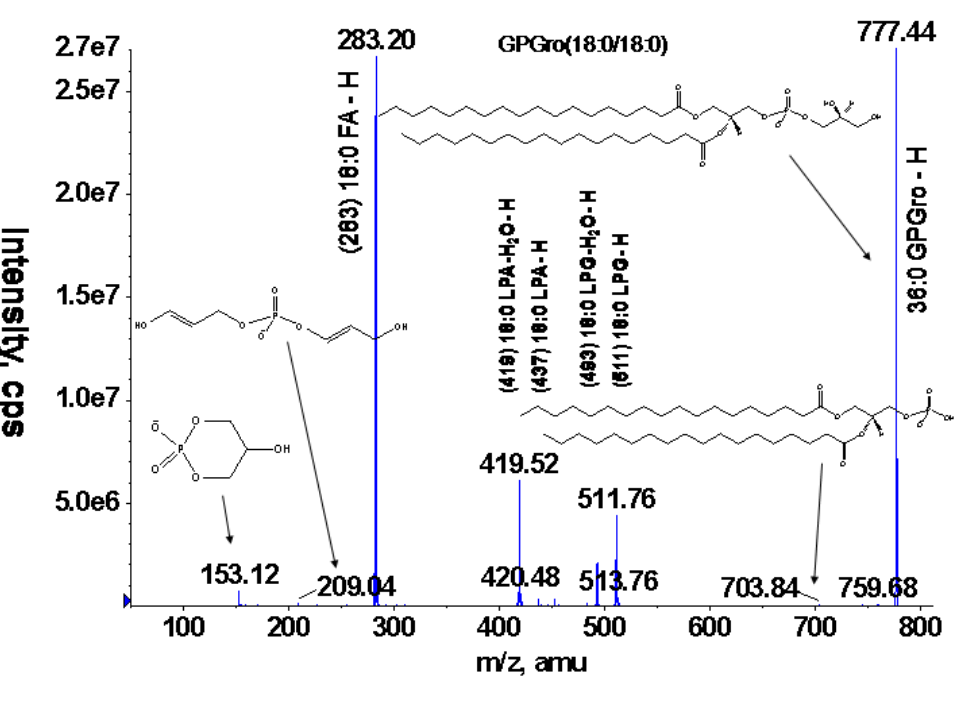 |
|  | 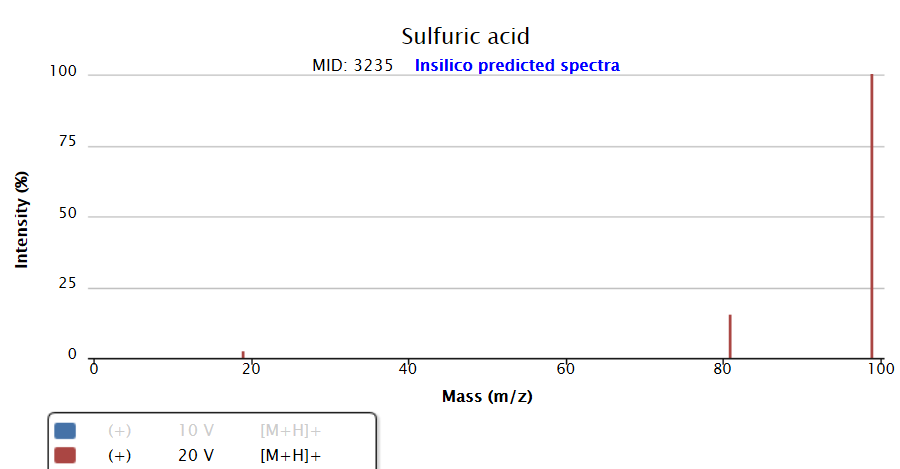 |
|    | 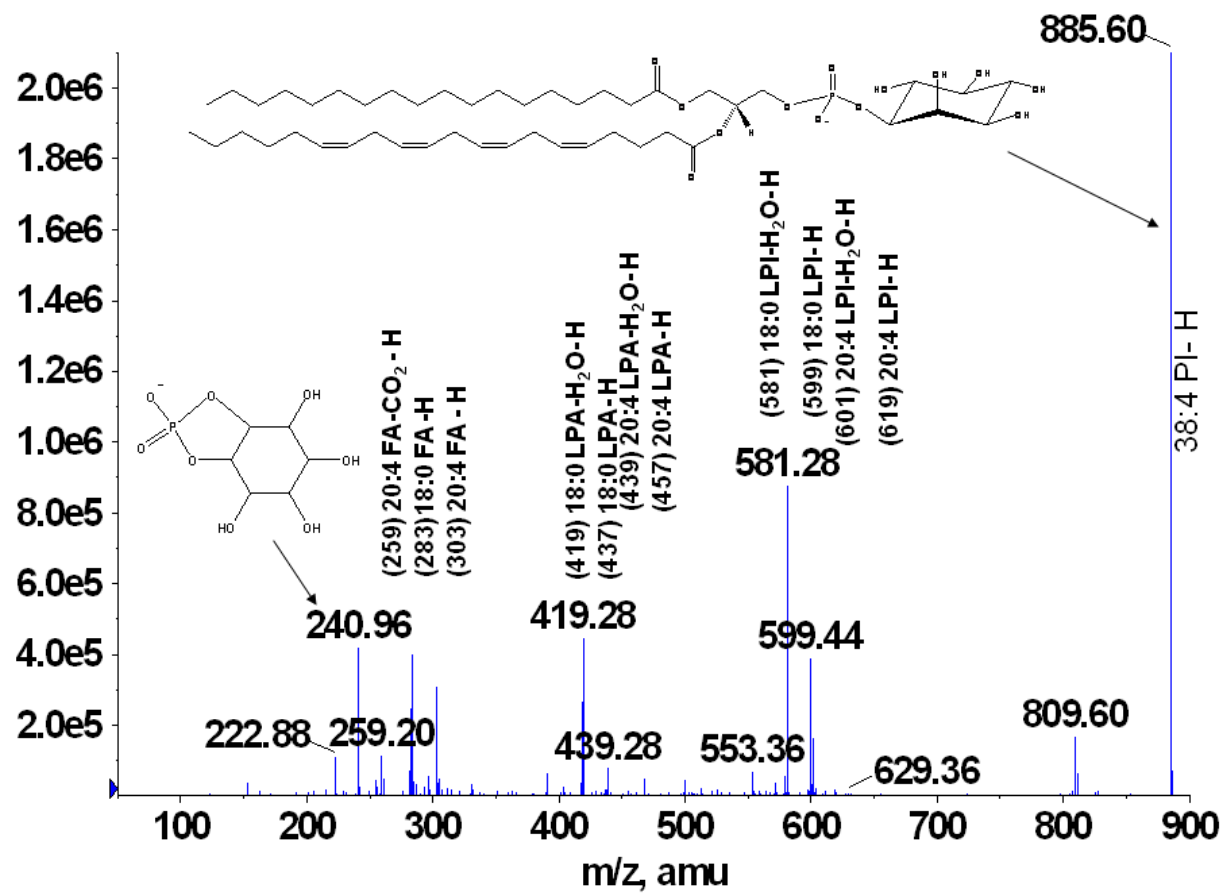 |
|    | 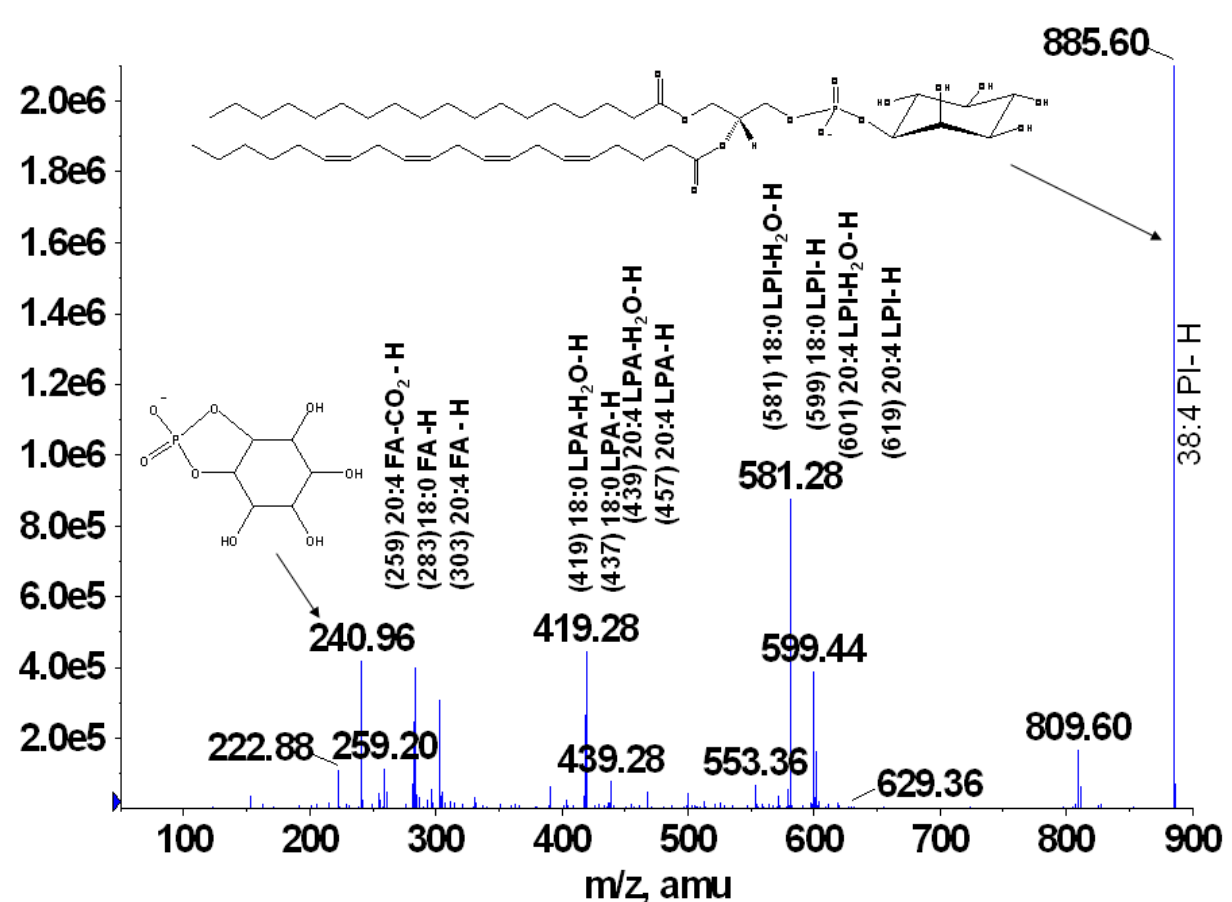 |
|    | 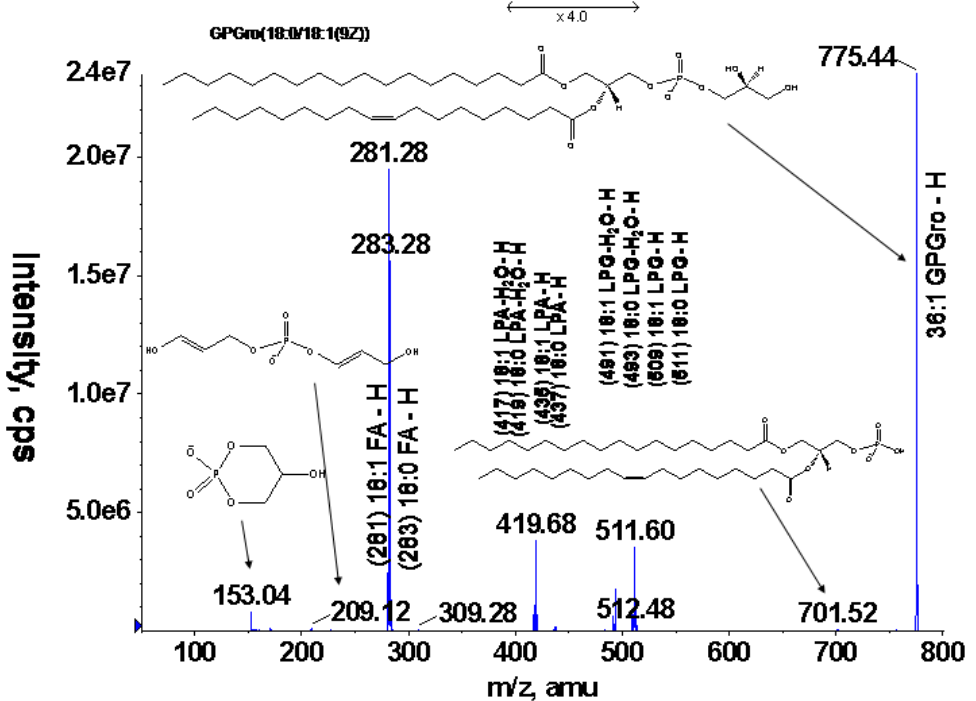 |
|    |  |
|  |  |
|  |  |
|    |  |
|  |  |
|    |  |
|  | 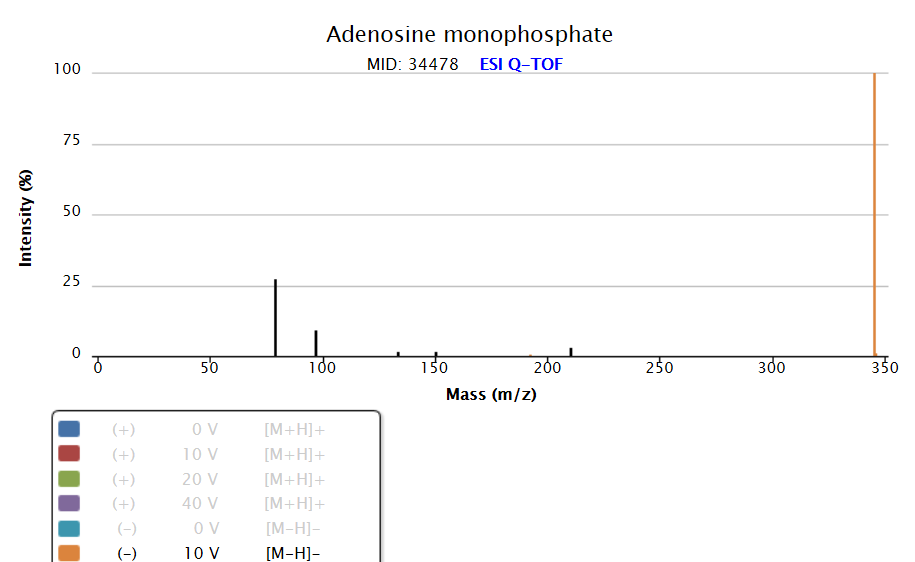 |
|  | 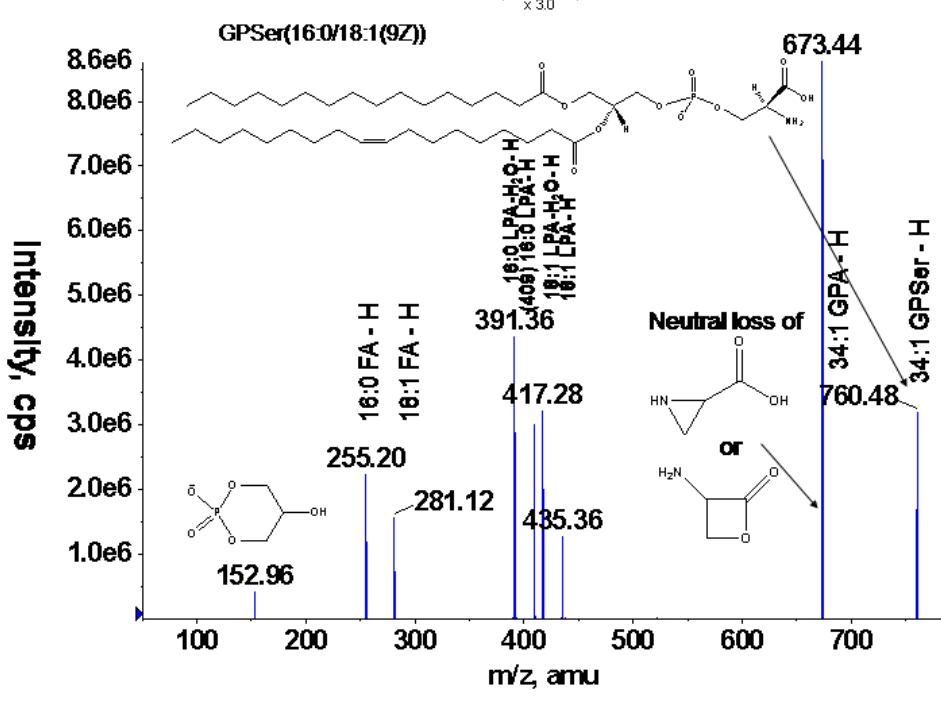 |
|  | 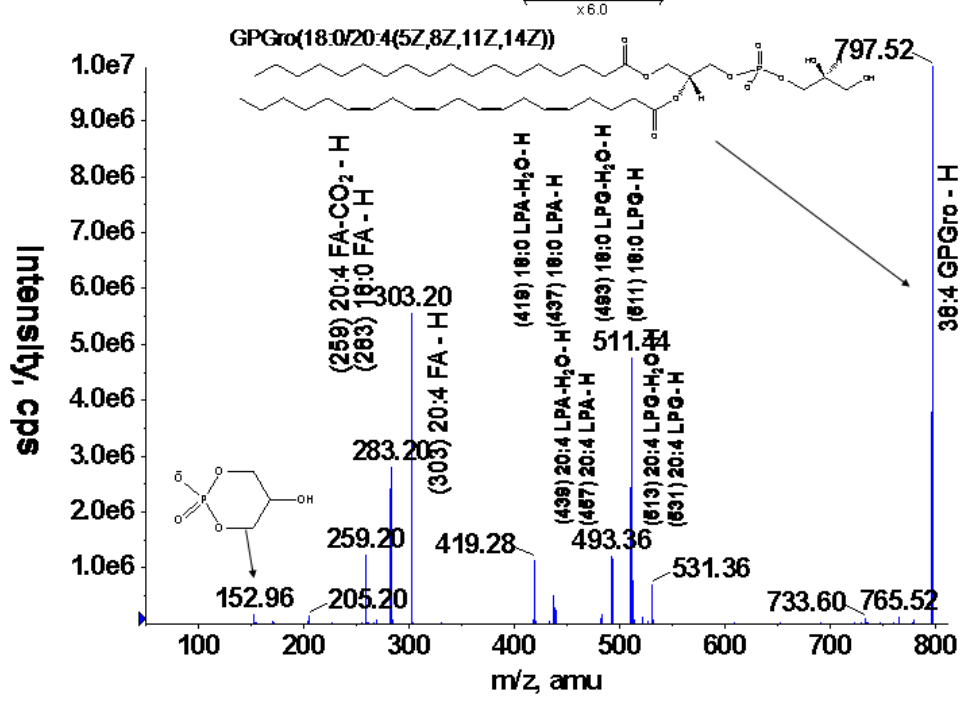 |
|    |  |
|   |  |
|  | 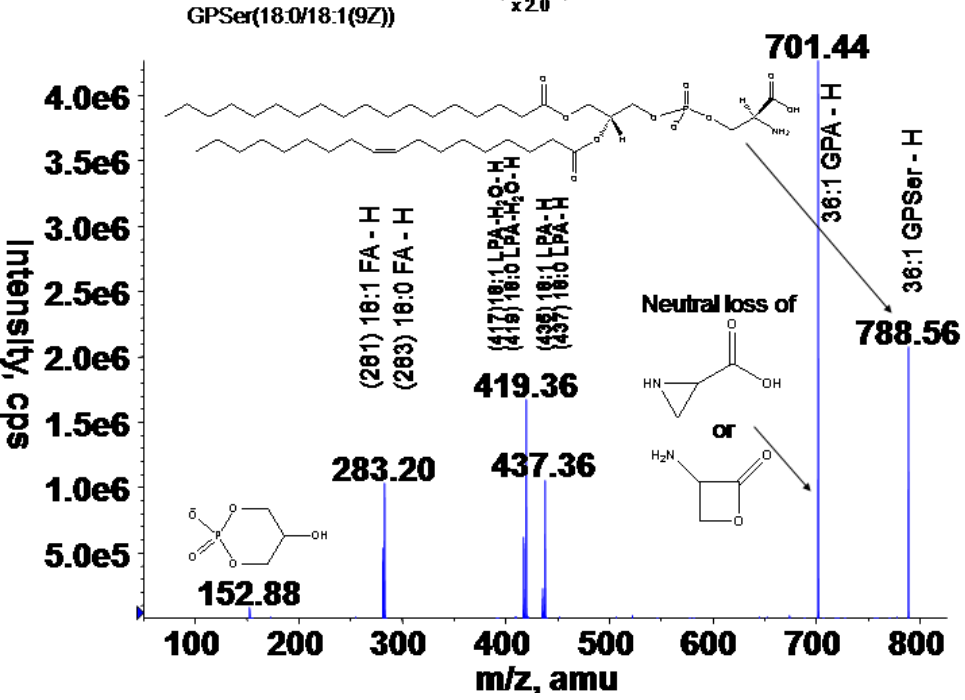 |
|  | 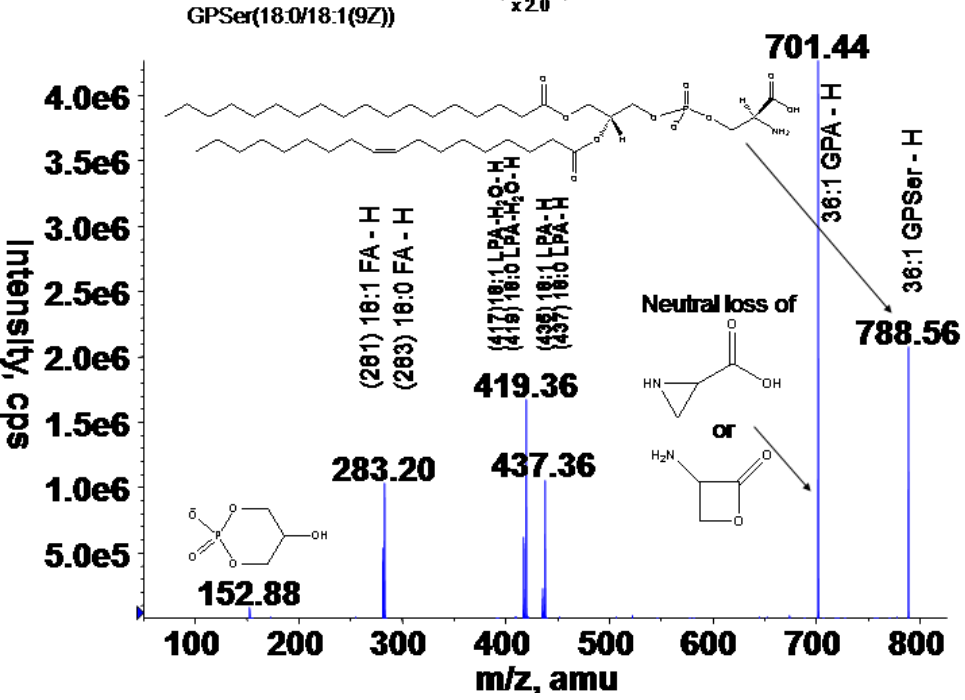 |
|  | 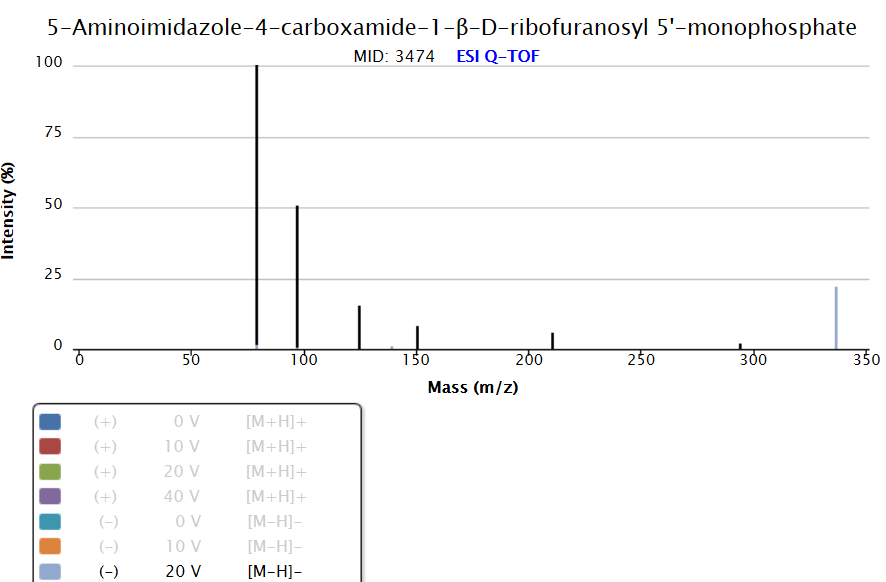 |
|  |  |
|  |  |
|  | 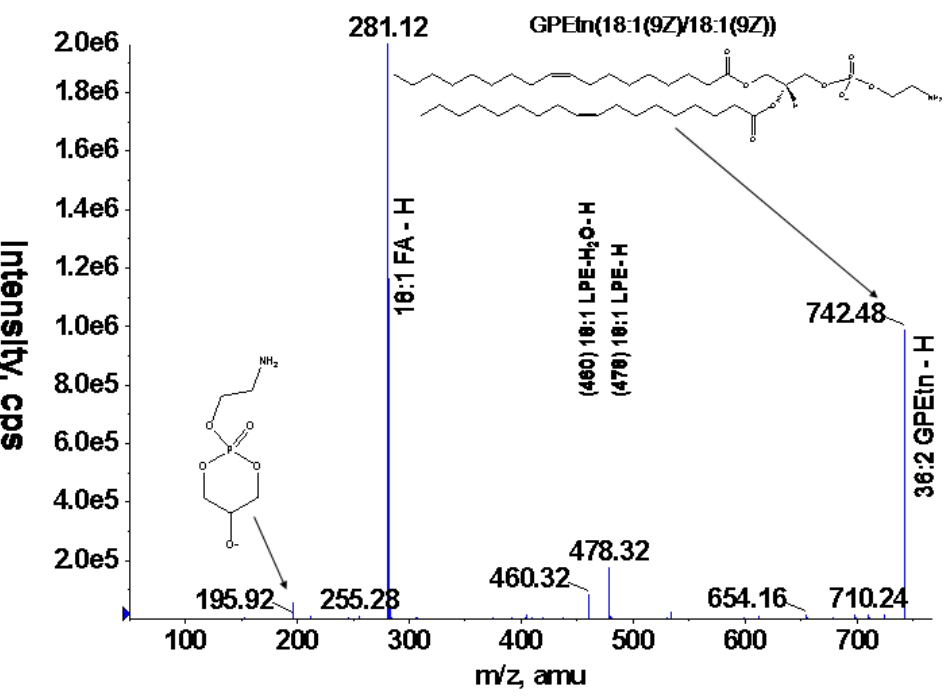 |
|  |  |
|  | 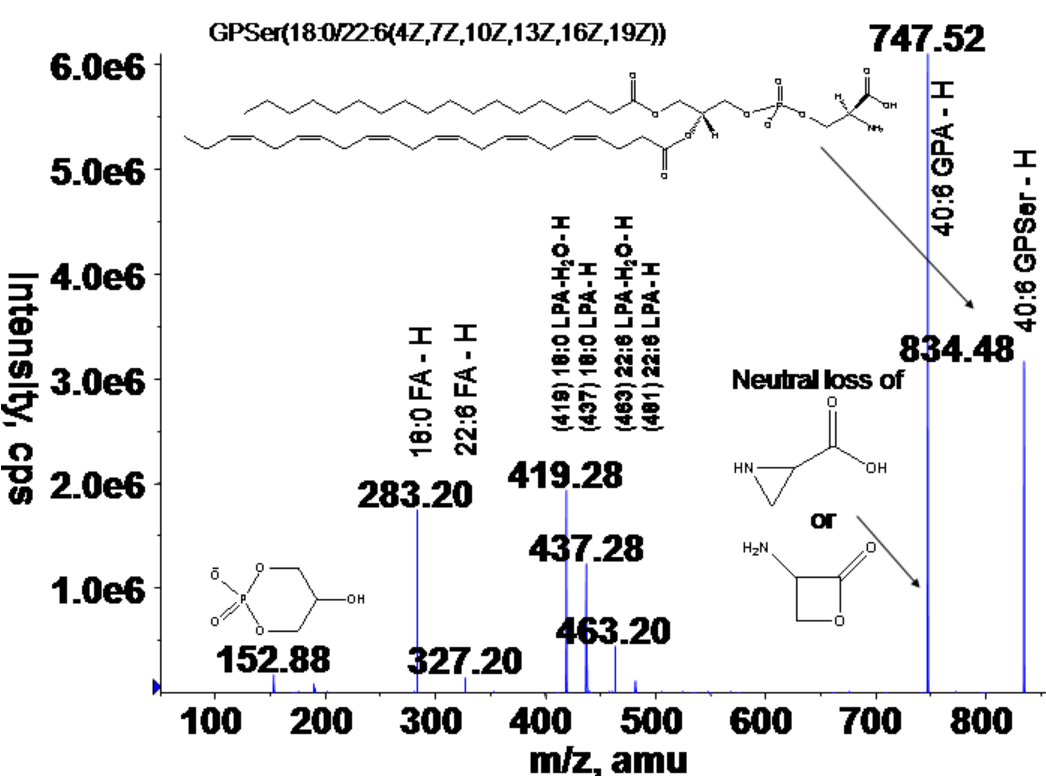 |
|  | 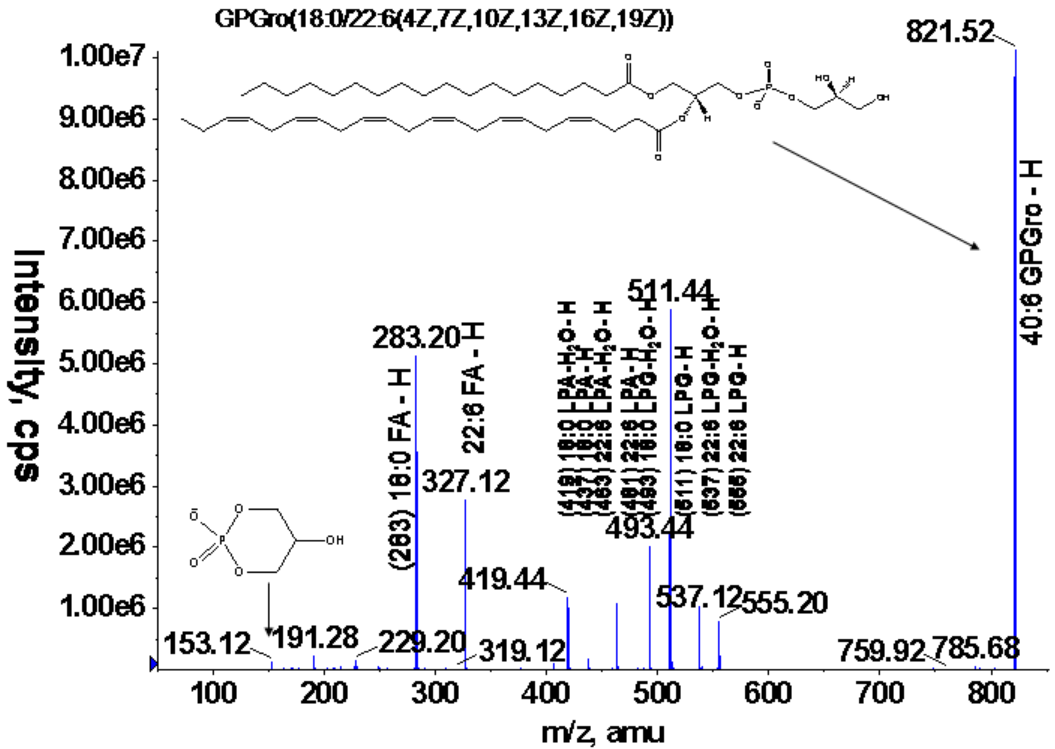 |
|  |  |
|  |  |
|  |  |
|  |  |
|  |  |
|  | 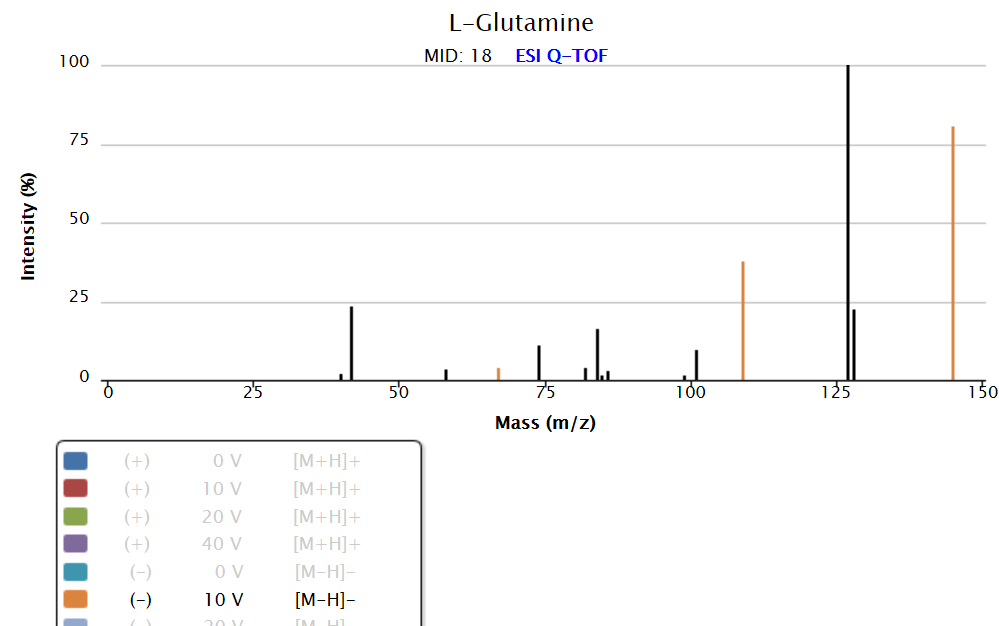 |
|  | 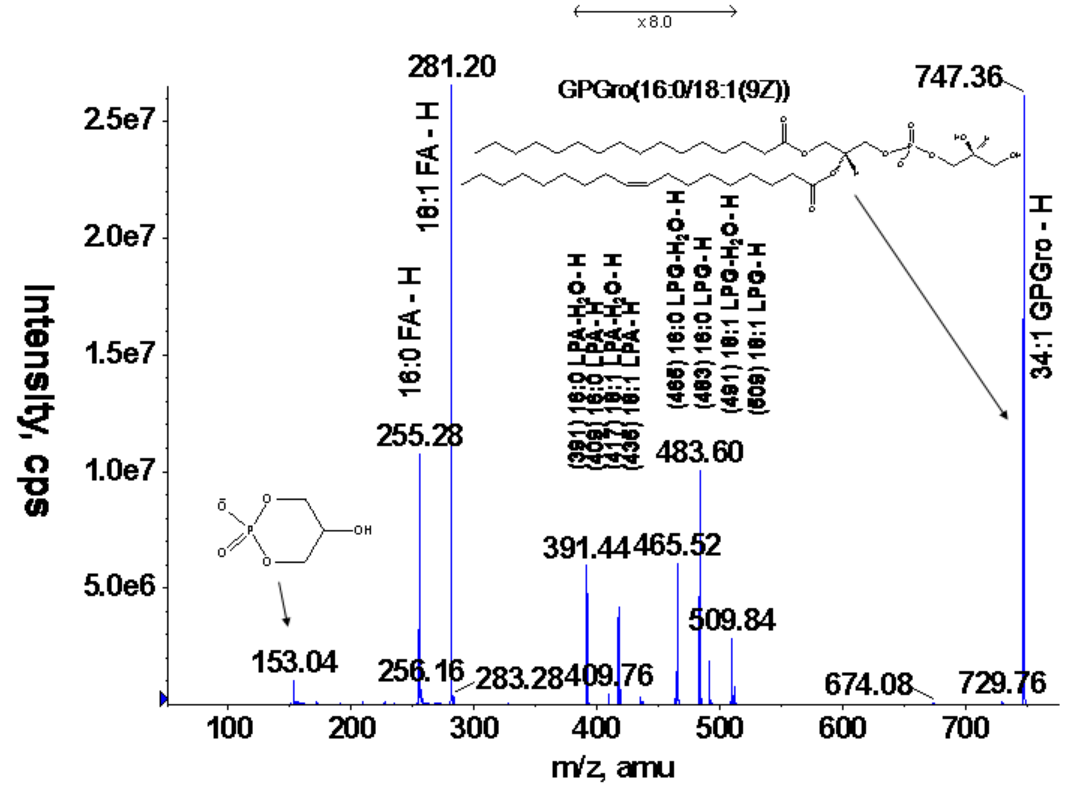 |
|  | 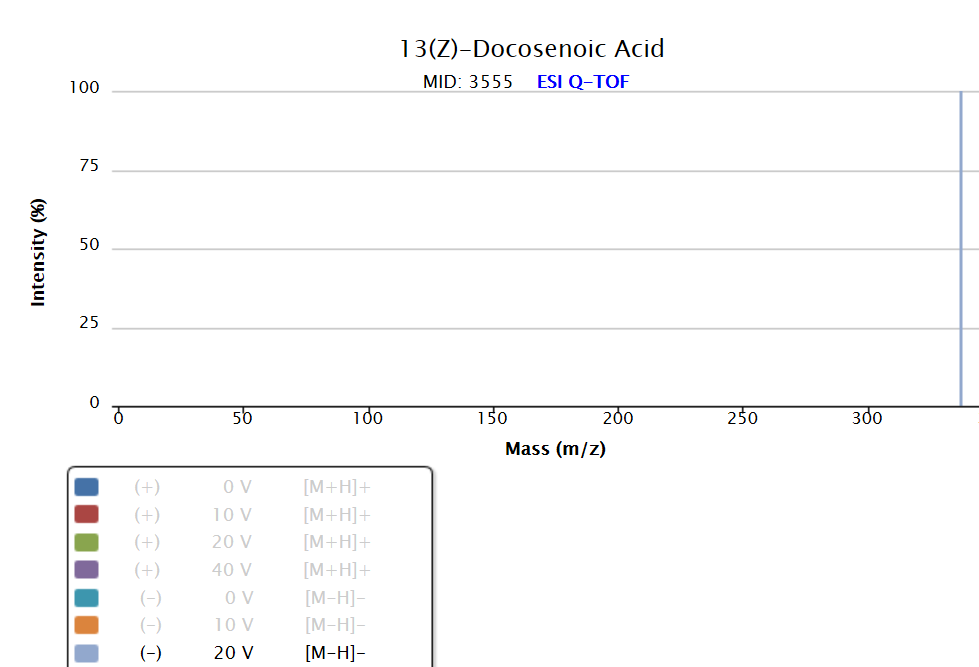 |
|  |  |
|  | 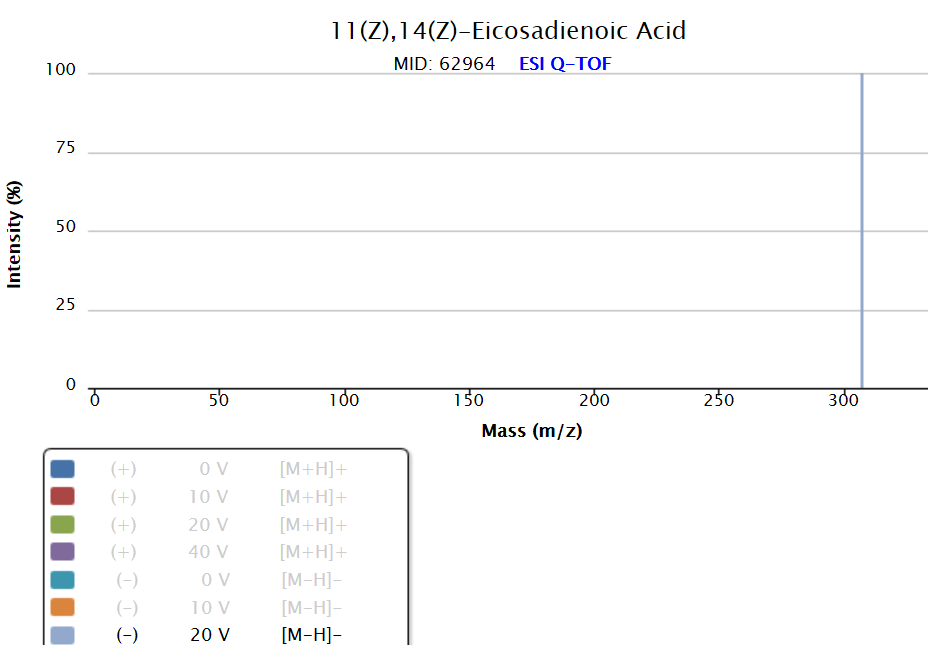 |
|  |  |
|  | 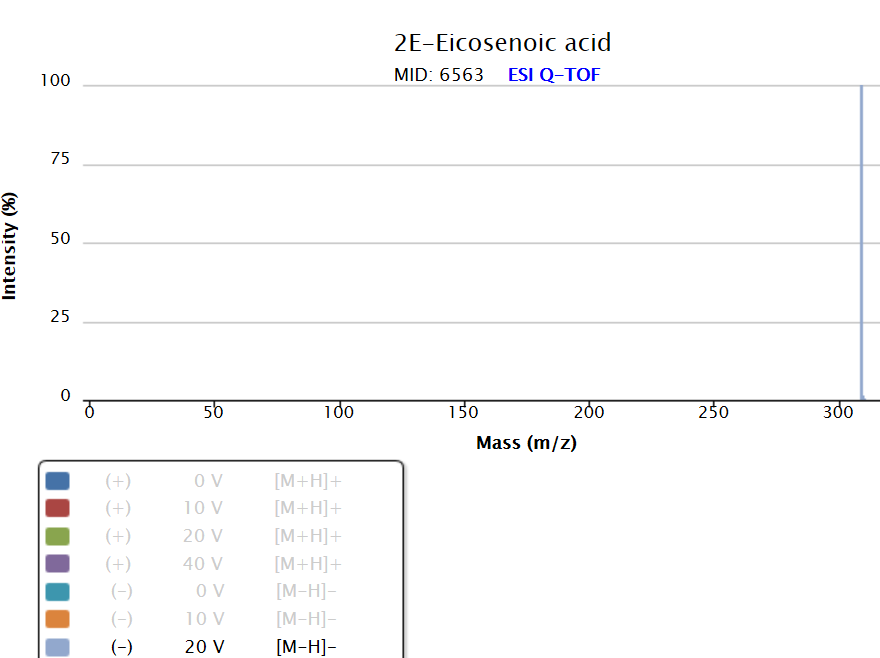 |
|  |  |
|  |  |
|  |  |
|  |  |
|  | 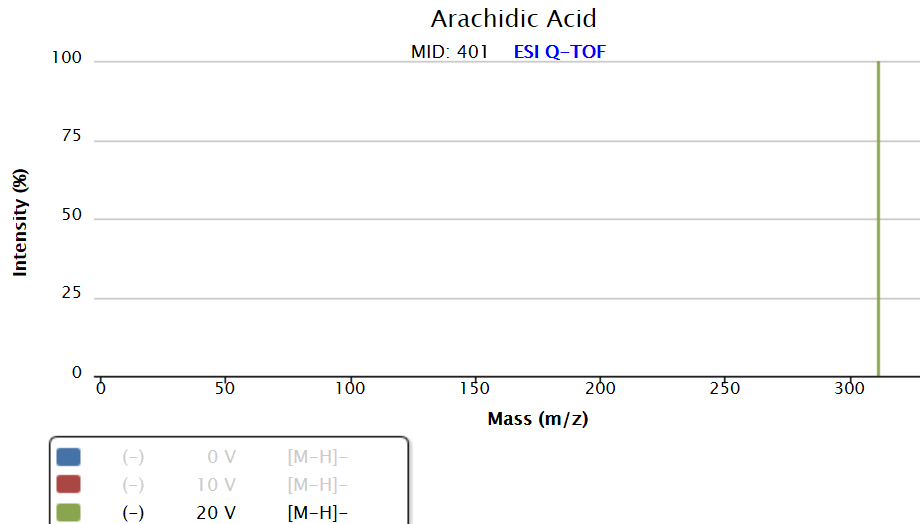  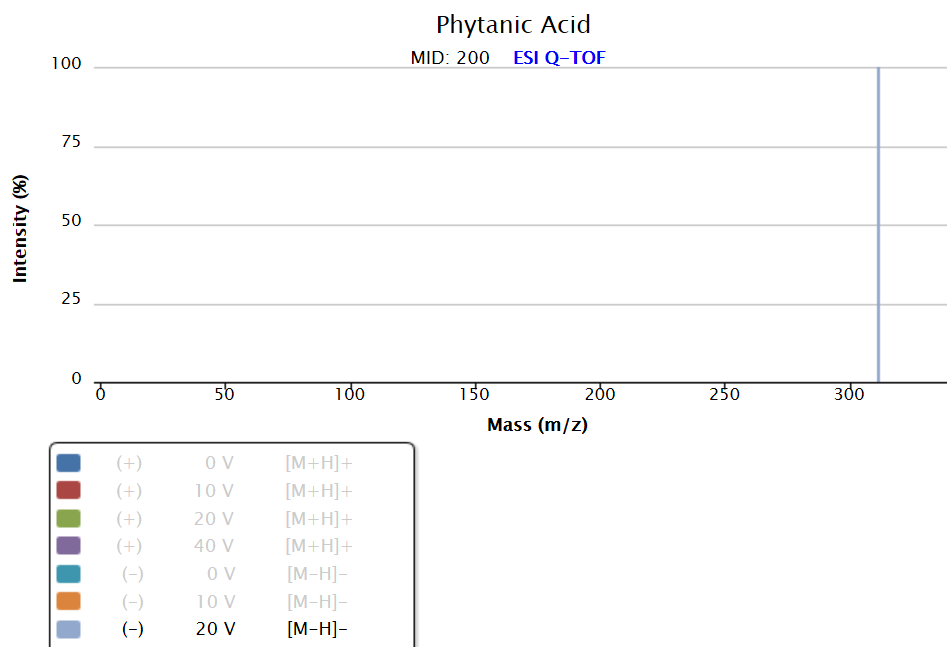 |
|  | 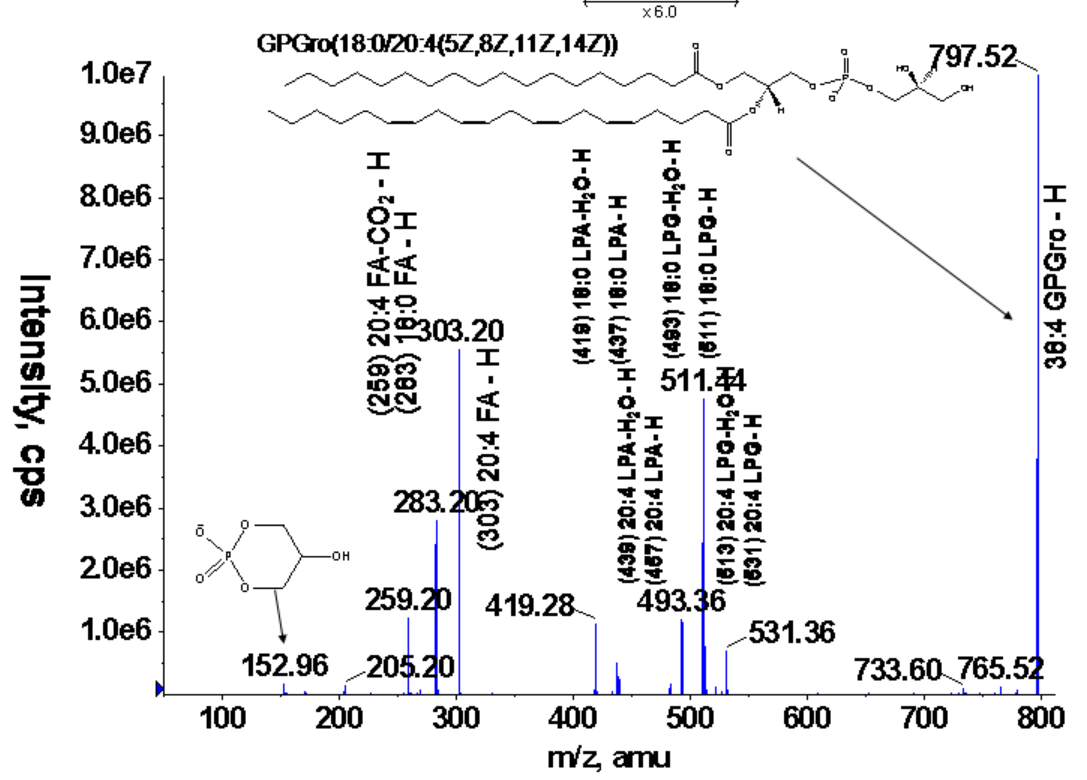 |
|  | 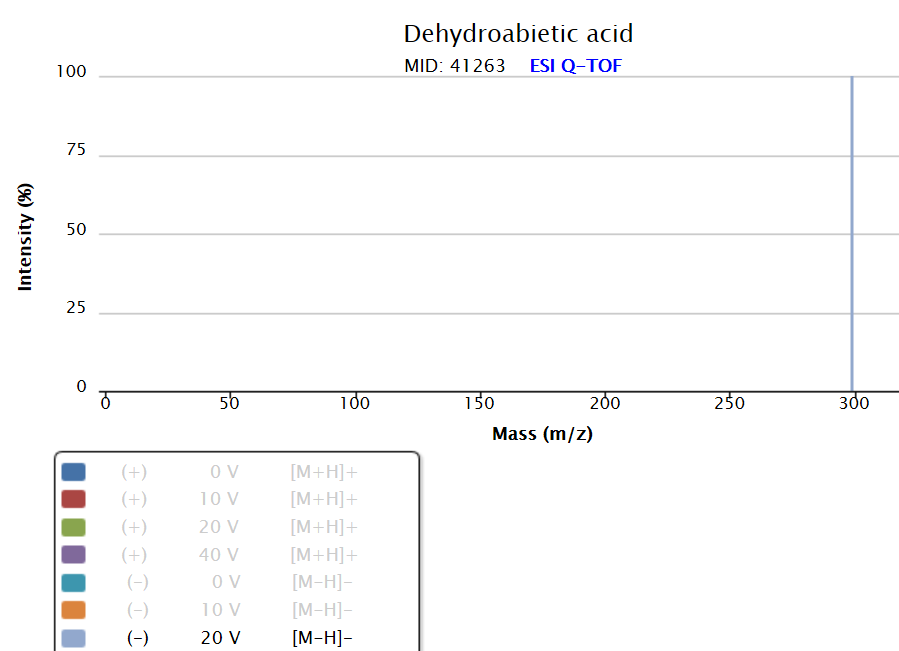 |
|  | 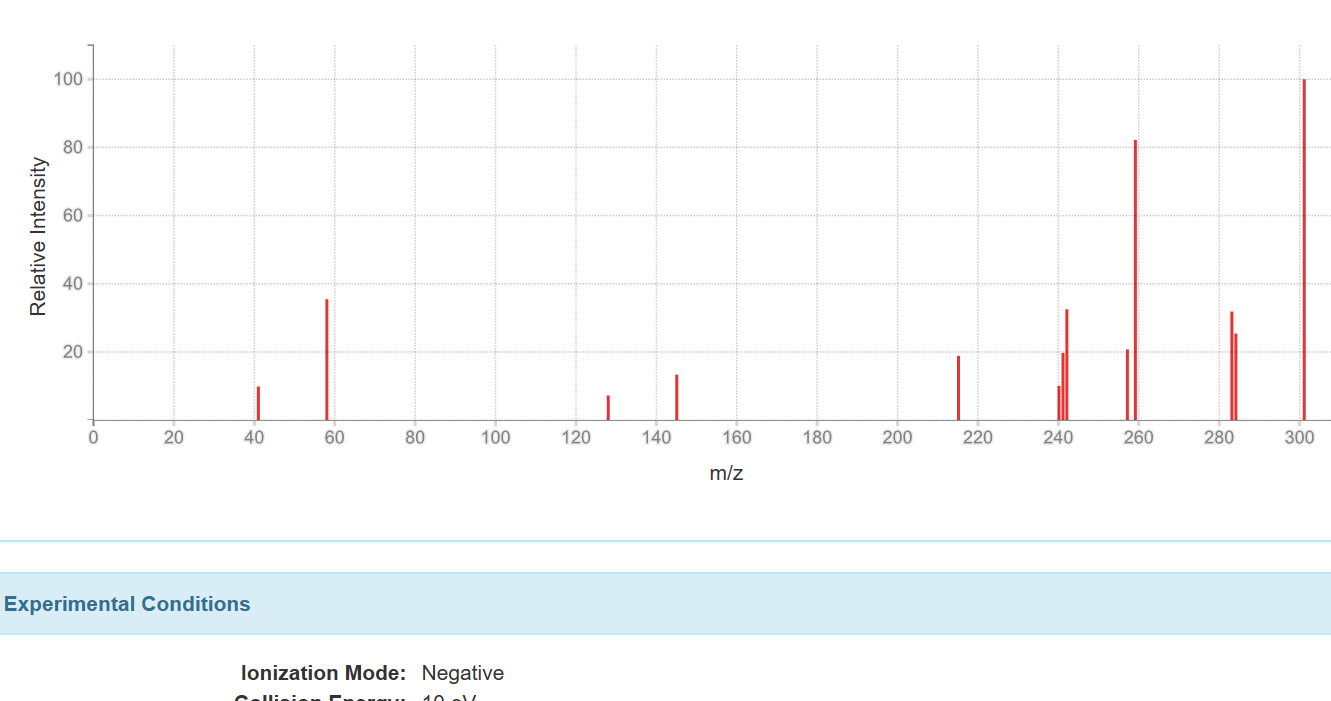 |
|  |  |
|  |  |
|  | 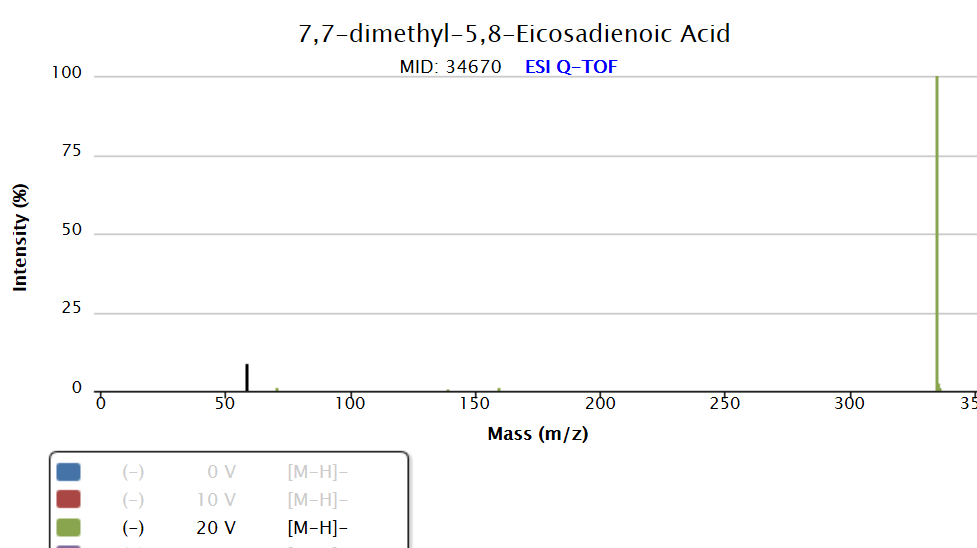 |
|  | 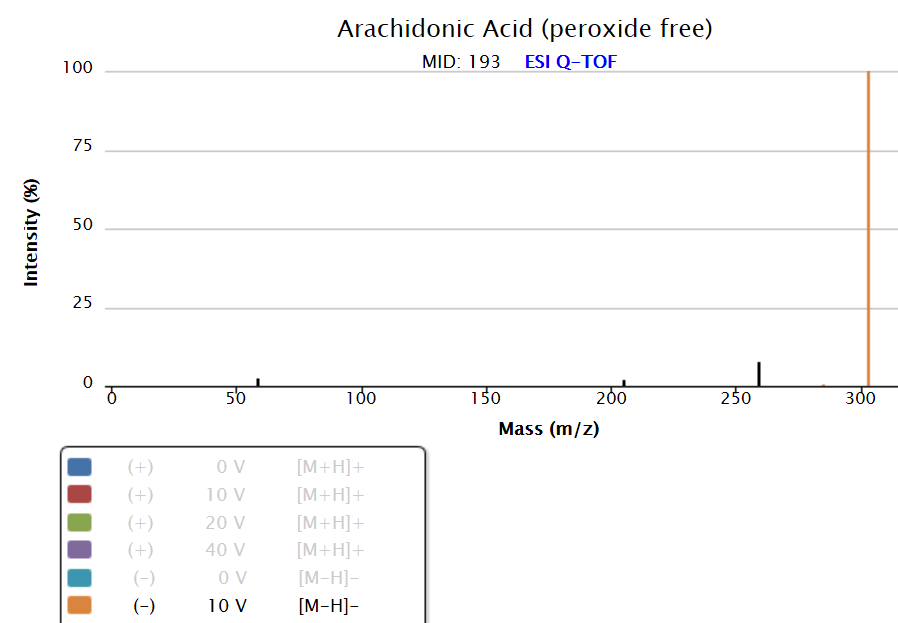 |
|  |  |
|  | 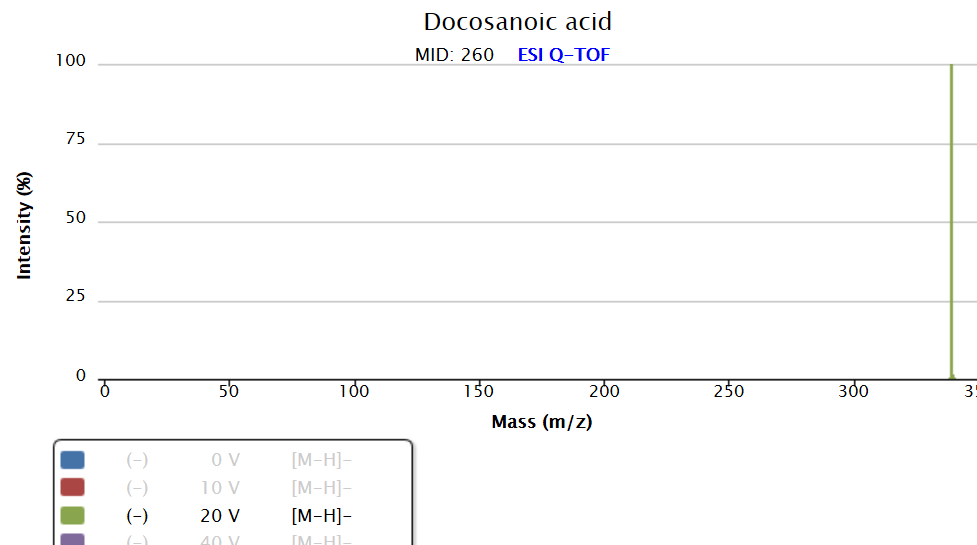 |
|  | 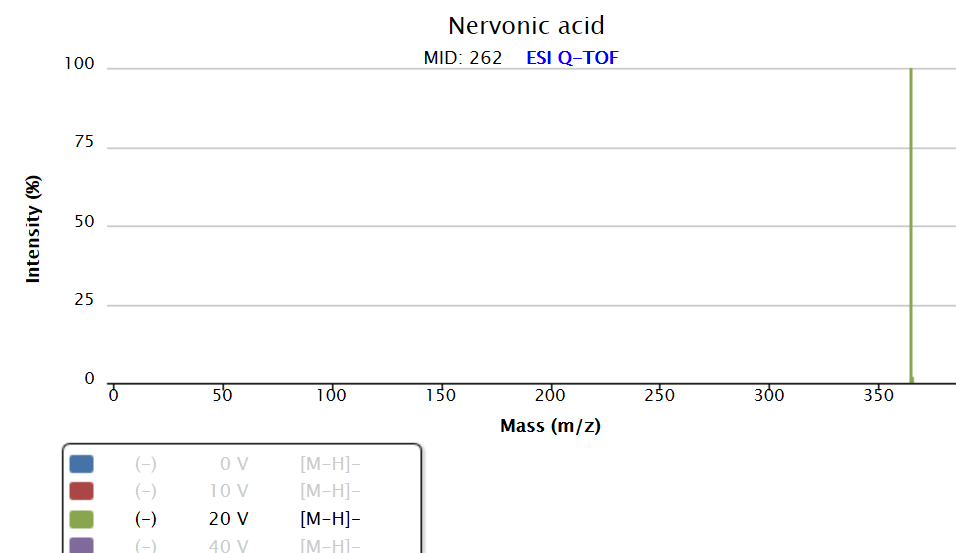 |
|  | 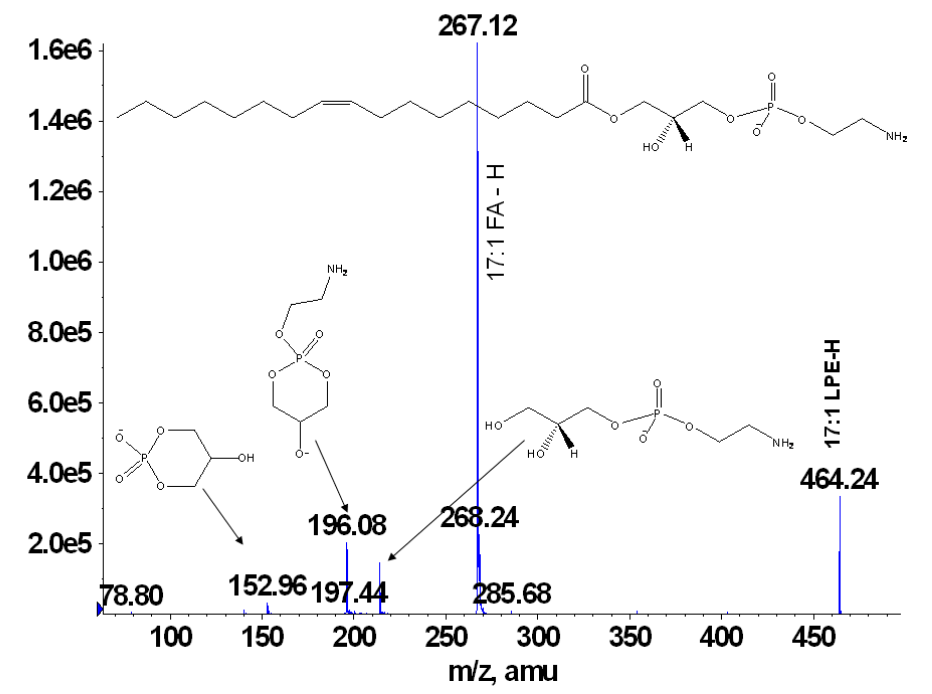 |
|  | 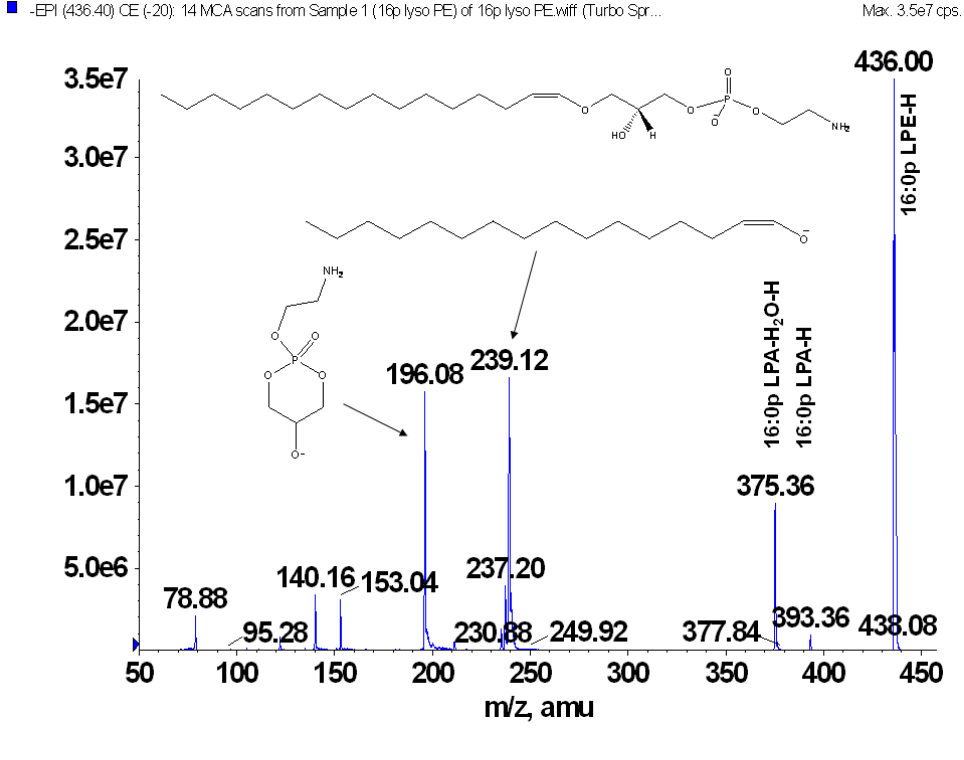 |
|  | 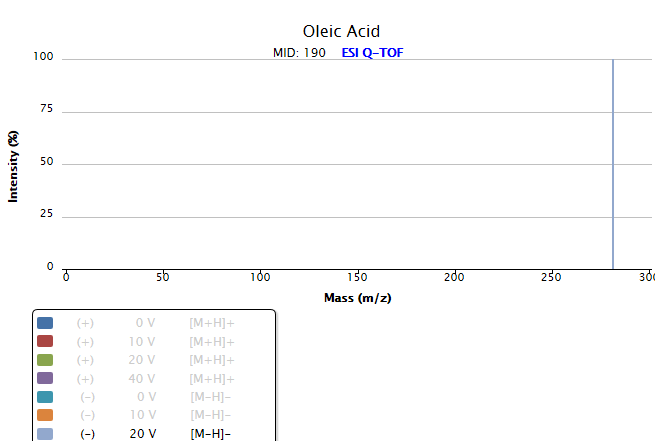 |
|  |  |
|  |  |
|  | 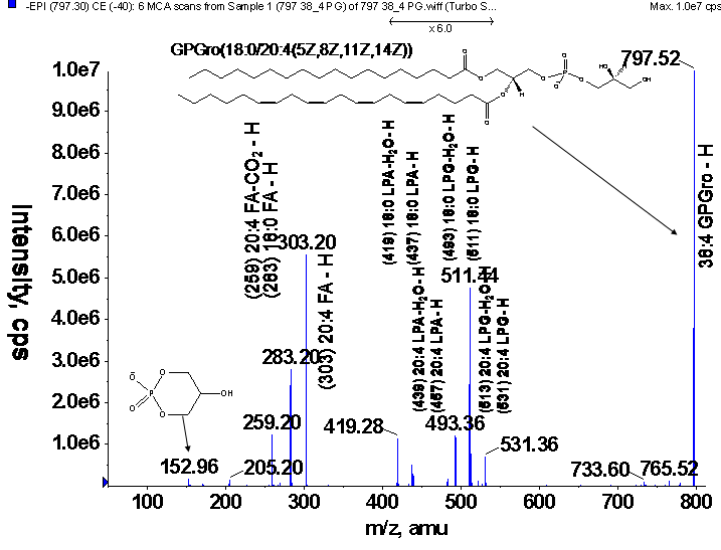 |
|  |  |
|  |  |
|  | 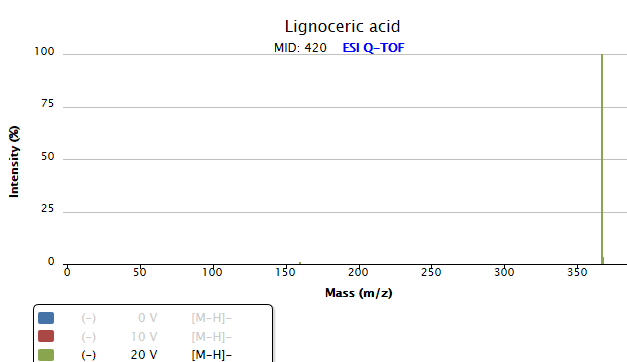 |
|  |  |
