# Supplementary figures and images for: A Novel Methoxybenzyl 5-Nitroacridone Derivative Effectively Triggers G1 Cell Cycle Arrest in Chronic Myelogenous Leukemia K562 Cells by Inhibiting CDK4/6-Mediated Phosphorylation of Rb
Source: Int J Mol Sci. 2020 Jul 18;21(14):5077. doi: 10.3390/ijms21145077 (PMC7403985; doi:10.3390/ijms21145077)

**
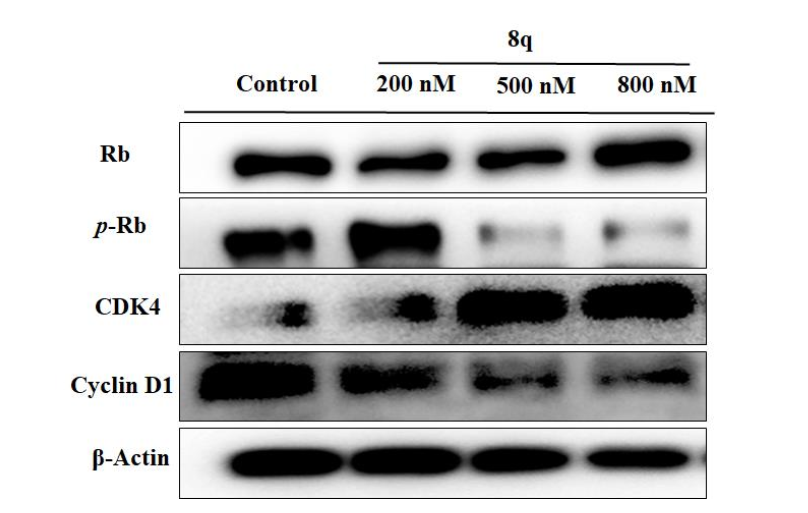
**

**
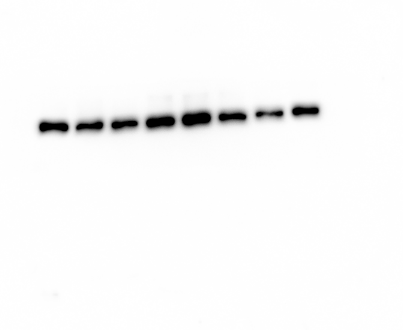

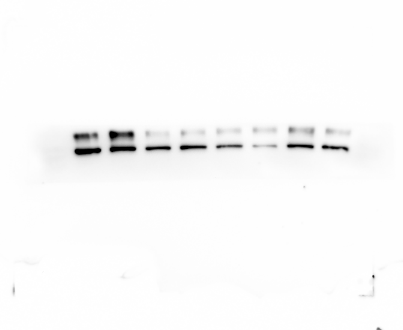
**

**Rb *p*-Rb**

**
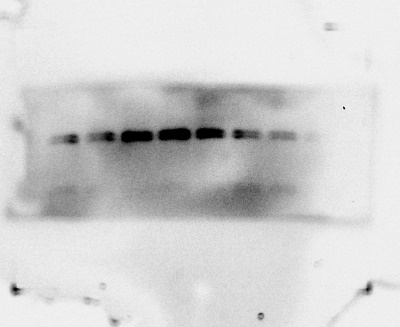
**
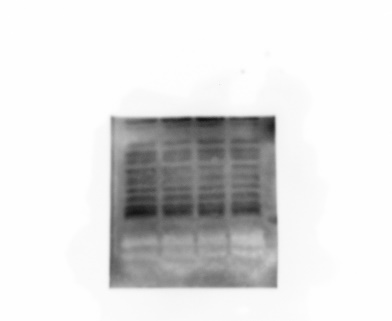


**CDK4 Cyclin D1**

**
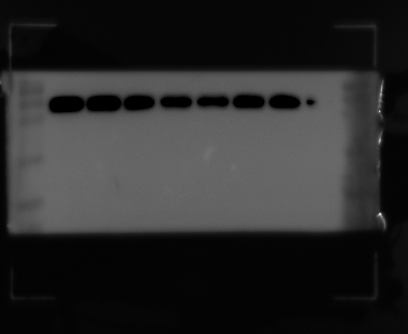
**

**β-Actin**

**
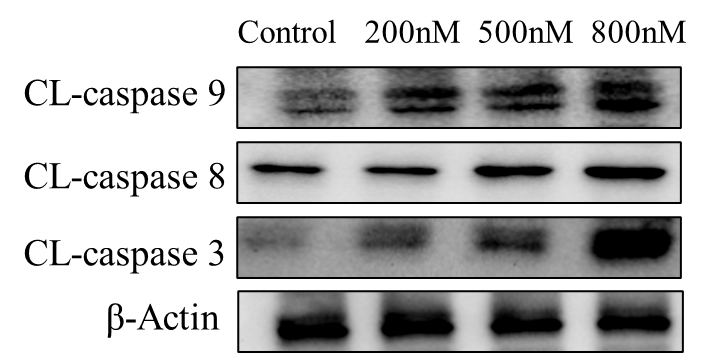
**


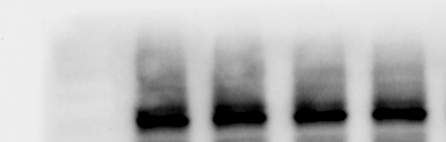
 **β-Actin**

**
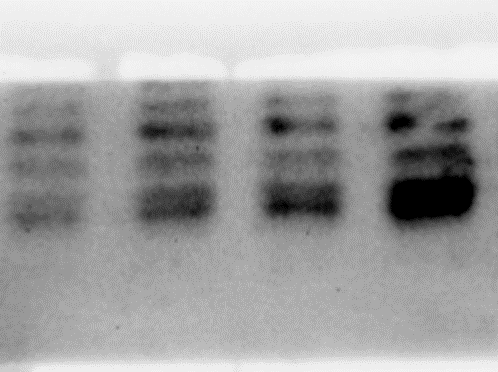
 CL-caspase 3**


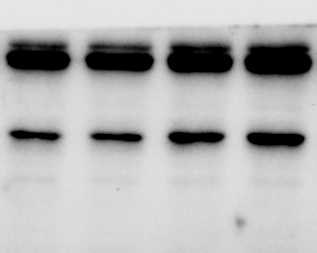
 **CL-caspase 8**

**
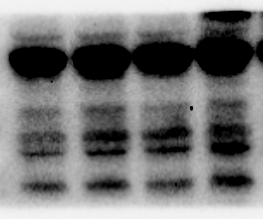
 CL-caspase 9**

**
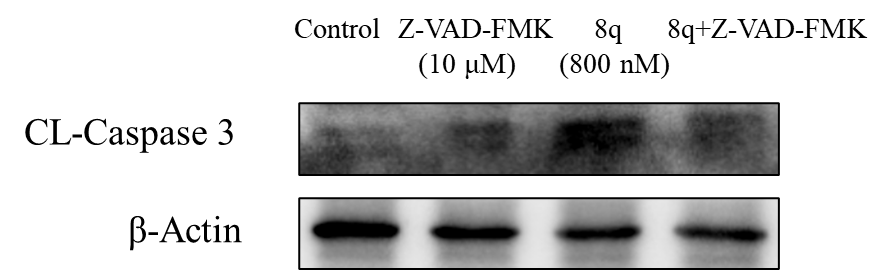
**

**
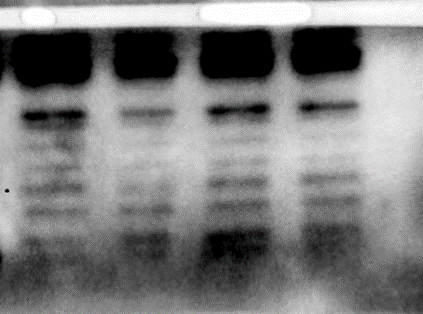
**

**CL-caspase 3**

**
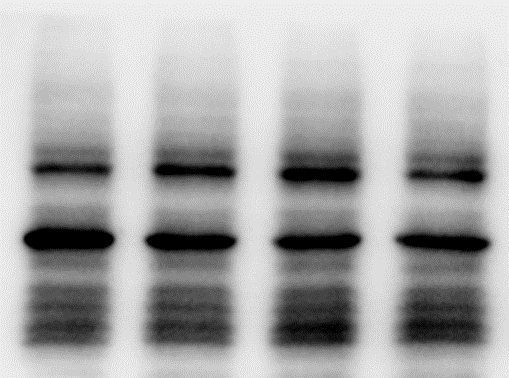
**

**β-Actin**

Supplement: Supplementary file 1 [file ijms-21-05077-s001.zip › Supplementary Files/Original images of western blots.docx]
